# Clinical epidemiology, genetic diversity, and drug susceptibility patterns by whole genome sequencing of *Mycobacterium tuberculosis* complex isolates in Gabon from 2012 to 2022

**DOI:** 10.1016/j.ijregi.2024.100501

**Published:** 2024-11-28

**Authors:** Bayode R. Adegbite, Viola Dreyer, Jabar B.P.A.A. Agbo, Rhett C. Mevyann, Guy A.R.I. Mfoumbi, Micheska E.D. Ndanga, Christopher M. Biyogho, Jean R. Edoa, Fabrice Beral M'Baidiguim, Andréa R.O. Obele Ndong, Abraham S. Alabi, Peter G. Kremsner, Ayola A. Adegnika, Stefan Niemann, Martin P. Grobusch

**Affiliations:** 1 Centre de Recherches Médicales de Lambaréné, Lambaréné, Gabon; 2 Center of Tropical Medicine and Travel Medicine, Department of Infectious Diseases, Amsterdam University Medical Centers, location Amsterdam, Amsterdam Infection & Immunity, Amsterdam Public Health, University of Amsterdam, Amsterdam, The Netherlands; 3 Institut für Tropenmedizin, Eberhard Karls Universität Tübingen and German Center for Infection Research (DZIF), Tübingen, Germany; 4 Forschungszentrum Borstel, Leibniz Lungenzentrum, Parkallee 1, Borstel, Germany; 5 German Center for Infection Research (DZIF), Partner Site Hamburg-Lübeck-Borstel-Riems, Borstel, Germany; 6 Department of Parasitology, Leiden University Medical Center, Leiden, The Netherlands; 7 Masanga Medical Research Unit, Masanga, Sierra Leone; 8 Institute of Infectious Diseases and Molecular Medicine, University of Cape Town, Cape Town, South Africa

**Keywords:** Gabon, *Mycobacterium africanum*, *Mycobacterium tuberculosis* complex (Mtbc), Whole genome sequencing, MDR-TB

## Abstract

**Objectives:**

This study aimed to investigate genotypic characteristics and drug resistance profiles of *Mycobacterium tuberculosis* complex (Mtbc) strains isolated from patients with suspected tuberculosis (TB) in Gabon.

**Methods:**

We performed whole genome sequencing of 430 Mtbc strains cultured between 2012 and 2022. Phylogenetic strain classification, genomic resistance prediction, and cluster analysis were also performed.

**Results:**

Strains from four major Mtbc lineages, L4 (n = 372; 65%), L5 (n = 46; 11%), L2 (n = 6; 1,4%), and L6 (n = 3; 0,7%), were observed. Interestingly, more than 10% of the strains were represented by *Mycobacterium africanum* strains (L5 and L6), with L4 strains being dominant. The subclassification of L4 strains showed that L4.6.2.2 (Cameroon, n = 116) was the most prevalent. The proportion of resistance (any resistance to first-line TB drugs) was 30%, with 12% (n = 52) being at least multidrug-resistant (MDR) and six showing additional fluoroquinolone resistance. The overall cluster rate was 64% in non-MDR and MDR Mtbc strains.

**Conclusion:**

Although most Mtbc infections are caused by L4 *M. tuberculosis* strains, *M. africanum* strains also contribute to TB epidemiology in Gabon. The MDR epidemic is mainly driven by one dominant L4 Haarlem outbreak clone that has been spreading for approximately a century in the country.

## Highlights

• Four major *Mycobacterium tuberculosis* complex lineages are identified L2, L4, L5, and L6 in Gabon.
• There is a notable presence of *Mycobacterium africanum* (L5 and L6).
• The L4.6.2.2 (Cameroon) strain was the most prevalent.
• Two significant multidrug-resistant *Mycobacterium tuberculosis* complex outbreaks belong to the Haarlem and Beijing sub-lineages.

## Introduction

Despite the aim of reducing the incidence of tuberculosis (TB) and deaths by 2035, TB remains a global threat to public health and among the deadliest infectious disease killers worldwide. According to the World Health Organization (WHO), a total of 10.6 million people worldwide fell ill with TB in 2022, killing 1.3 million people [1]. The emergence of *Mycobacterium tuberculosis* complex (Mtbc) strains resistant to first- and second-line treatment regimens has been an increasing challenge to global TB control in many countries [2]. In 2022, there were over 400,000 cases of rifampicin-resistant (RR), multidrug-resistant (MDR) (resistance to isoniazid and rifampicin), pre-extensively drug-resistant (pre–XDR-TB, MDR plus resistance to any fluoroquinolone [FQ]), and XDR-TB (pre-XDR plus additional resistance to at least one of the additional group A drugs, e.g. bedaquiline or linezolid) worldwide [3], linked with high treatment failure rates and increased mortality. However, data regarding the reasons for this development are missing in several high-incidence regions.

Molecular epidemiology is a highly valuable tool for improving surveillance and control of TB. High-resolution genotyping, e.g. based on whole genome sequencing (WGS), can be used for a variety of study questions, e.g. the distinction between reinfection and relapse [4], local and global phylogeny, resistance levels and evolution, or transmission analysis, including the definition of dominant clones in a particular area or even across country borders [5]. When the global phylogeny of Mtbc strains is considered, strains of nine different lineages are known to cause TB in humans [6]: Indo-Oceanic (lineage 1), East-Asian (lineage 2), East-African-Indian (lineage 3), Euro-American (lineage 4), West African 1 (lineage 5), West African 2 (lineage 6), Ethiopian (lineage 7), lineage 8, and lineage 9 [7]; lineage 8 and lineage 9 were reported most recently from the Central and Eastern African regions, respectively. Strains of lineage 4 (L4) are reported to be the most common worldwide [8]. Considering the pathobiology of strains of different Mtbc lineages, there are variations, e.g. in *in vitro* growth rate [9], pathogenicity *in vitro* models, and the ability to acquire and transmit drug resistance among Mtbc strains [10].

Gabon is a high-burden TB country with an estimated TB incidence of 509 cases per 100,000 people [11]. In 2022, a total of 12,000 TB cases were notified, of these 5% were MDR-TB [3]. A recent study over 8 years (2014-2021) on the trends of drug-resistant (DR) TB in Gabon reported that one-third of patients with drug-resistant TB were treatment-naïve [12]. Although this is pointing toward the primary transmission of drug-resistant TB in the country, no molecular epidemiological data from the government are available.

To address this question, we performed WGS of 430 Mtbc strains obtained from patients with TB living in Gabon between 2012 and 2022 to determine the most common Mtbc lineage-causing TB diseases in Gabon. We get first insights into the country's drug resistance distribution and transmission dynamics.

## Material and methods

### Study design and period

This was a historical cross-sectional study using data collected between 2012 and 2022 in Lambaréné, Gabon at the National Tuberculosis Reference Laboratory located at the Centre de Recherches Médicales de Lambaréné (CERMEL). Samples were derived from patients with confirmed TB who presented with TB symptoms at CERMEL, the Albert Schweitzer Hospital, and the Hôpital Georges Rawiri in Lambaréné, Gabon, and hospitals across the country who shipped the sample to the Lambaréné Tuberculosis Reference Laboratory.

### Study population and variables

Symptomatic patients consulting any health care facilities in the Moyen-Ogooué region in Gabon were referred to the CERMEL tuberculosis laboratory for bacteriological confirmation. Patients were invited to provide two sputum samples as recommended by the national TB guidelines [13]. Patients willing to participate in the study were invited to sign an informed consent form after providing information on the objective of the study. No exclusion criteria were applied. Demographic and clinical information were obtained for all study participants by a TB clinic nurse during the interview using a standardized questionnaire. Only sputum from patients with positive *Mycobacterium tuberculosis* smear microscopy, MTB RIF Xpert, or with clinical signs and radiology findings consistent with TB were stored for further sample processing.

### Sample processing and culturing

Samples for mycobacterial investigation were first analyzed by microscopy or Xpert MTB/RIF, according to national guidelines. The remaining portion was stored at −80°C and shipped to a supranational laboratory at the German National Reference Center for Mycobacteria in Borstel, Germany. Approximately 5 ml of sputum samples were homogenized by digestion for 1 minute at room temperature with 1 ml of N-acetyl L-cysteine (25 mg/ml) in phosphate buffer (pH 6.8) and vortexed with glass beads for 30 seconds. A 5-ml aliquot was decontaminated using 1% NaOH and centrifuged at 4000 × g for 15 minutes. To prepare the inoculum for cultures, the sediment was reconstituted in 2.5 ml phosphate buffer (pH 6.8). Sputum was cultured using Löwenstein-Jensen growth medium [14] and genomic DNA was extracted from sputum cultures using the standard cetyltrimethylammonium bromide–NaCl method.

### WGS and data analysis

Extracted genomic DNA of the Mtbc strains was processed using the Nextera XT library preparation kit, following the manufacturer's instructions and sequenced with one of Illumina's (San Diego, CA, USA) sequencers, MiSeq, HiSeq, or NextSeq 500. Strains were sequenced paired-end and a minimum average genome coverage of 50 × was aimed for. Raw read data were processed using MTBseq (v.1.10) [15] using standard parameters. Briefly, raw reads were mapped to the *M. tuberculosis* H37Rv reference sequence (GenBank ID: NC_000962.3) using the Burrows-Wheeler Aligner [16] and initial mapping was further refined using tools from the Genome Analysis Toolkit [17] and SAMtools [18]. Variants were called if supported by at least four reads in forward- and four reads in reverse orientation, and four reads calling the allele with at least a Phred score of ≥20 and a minimum allele frequency of 75%. Phylogenetic lineages (Mtbc lineages and known Beijing subgroups) were inferred from specific single nucleotide polymorphisms (SNPs) based on Merker *et al.* [19]. For resistance prediction, we used the MTBseq option to call low-frequency variants with the parameters set to –mincovf 1, –mincovr 1, –minphred20 1, and –minfreq 5. The annotation was mainly based on the WHO mutation catalog, with some additions from the MTBseq resistance catalog. A concatenated sequence alignment (PPE/PGRS and drug resistance–associated genes were excluded) was also calculated using the MTBseq step “TBjoin.”

We calculated a maximum likelihood tree using the concatenated sequence alignment and the IQtree software. We have used the automated ModelFinder option and ascertainment bias correction. We used an ultrafast bootstrap (UFBoot) approximation with 1000 replicates combined, with a further optimizing step to reduce the risk of overestimating the branch support. Phylogenetic trees were mid-point rooted using FigTree v1.4.4 and annotated using the online tool EvolView. Clustering was done using SNP differences of 5 to detect actual transmission events and 12 SNP differences to obtain a picture of former transmission events.

Mutations (small deletions and SNPs) in resistance-associated target regions were considered for molecular resistance prediction (Supplementary Table S1). Mutations in genes coding for the RNA polymerase subunits rpoA, rpoB (excluding resistance mediating mutations in the rifampicin resistance determining region and in codons 170, 400, and 491), and rpoC were reported as putative fitness compensating variants for RR strains as suggested previously.

### Data management and statistics

The data were collected and managed using REDCap (Research Electronic Data Capture) tools hosted at CERMEL. Data were analyzed using R Statistical software version 4.1.2 (R Foundation for Statistical Computing, Vienna, Austria). Means, SDs, and proportions were calculated for descriptive statistics.

## Results

### Study population

WGS was successfully performed for 430 Mtbc strains. Participant ages ranged from 8 to 78 years. The adolescent accounted for 5% (n = 19) of the study population (Table 1). The majority of patients fall into the age groups 19-30 and 31-40 years, with a proportion of 39% (n = 156) and 30% (n = 119), respectively. The patient population consisted of more men than women (n = 242, 56%). The smear grading was highly positive (+++ in 35% [151 of 430] and negative in 14% [60 of 430]) of cases. The proportion of HIV-TB coinfection was 16% (67 of 430) (Table 1).

**Table 1:** Population structure of *Mycobacterium tuberculosis* complex strains from Lambaréné and phylogenetic lineages according to the specific variants identified in the whole genome sequencing data.

Characteristics	430 (%)
**Age group (year)**	
8-18	19 (4.8%)
19-30	156 (39%)
31-40	119 (30%)
41-50	52 (13%)
51 and more	52 (13%)
Missing data	32
**Smear grading**	
negative	60 (14%)
+	69(16%)
++	98 (23%)
+++	151 (35%)
Missing data	52 (12%)
**Lineage**	
Lineage 2	6 (1%)
Lineage 4	372 (87%)
Lineage 5	46 (11%)
Lineage 6	3 (1%)
Unknown	3 (1%)
**Resistance profile**	
Drug sensitive tuberculosis	300 (70%)
Non-MDR	73 (17%
RR	3 (1%)
MDR	46 (11%)
Pre-XDR	6 (1%)
**SNP-Cluster D12**	
Clustered	330 (77%)
Not clustered	100 (23%)
**SNP-Cluster D5**	
Clustered	276 (64%)
Not clustered	154 (36%)

D, distance; MDR, multidrug-resistant; RR, rifampicin resistant; SNP, single nucleotide polymorphism; XDR, extensively drug resistant.

### Lineage distribution

**Figure 1:**
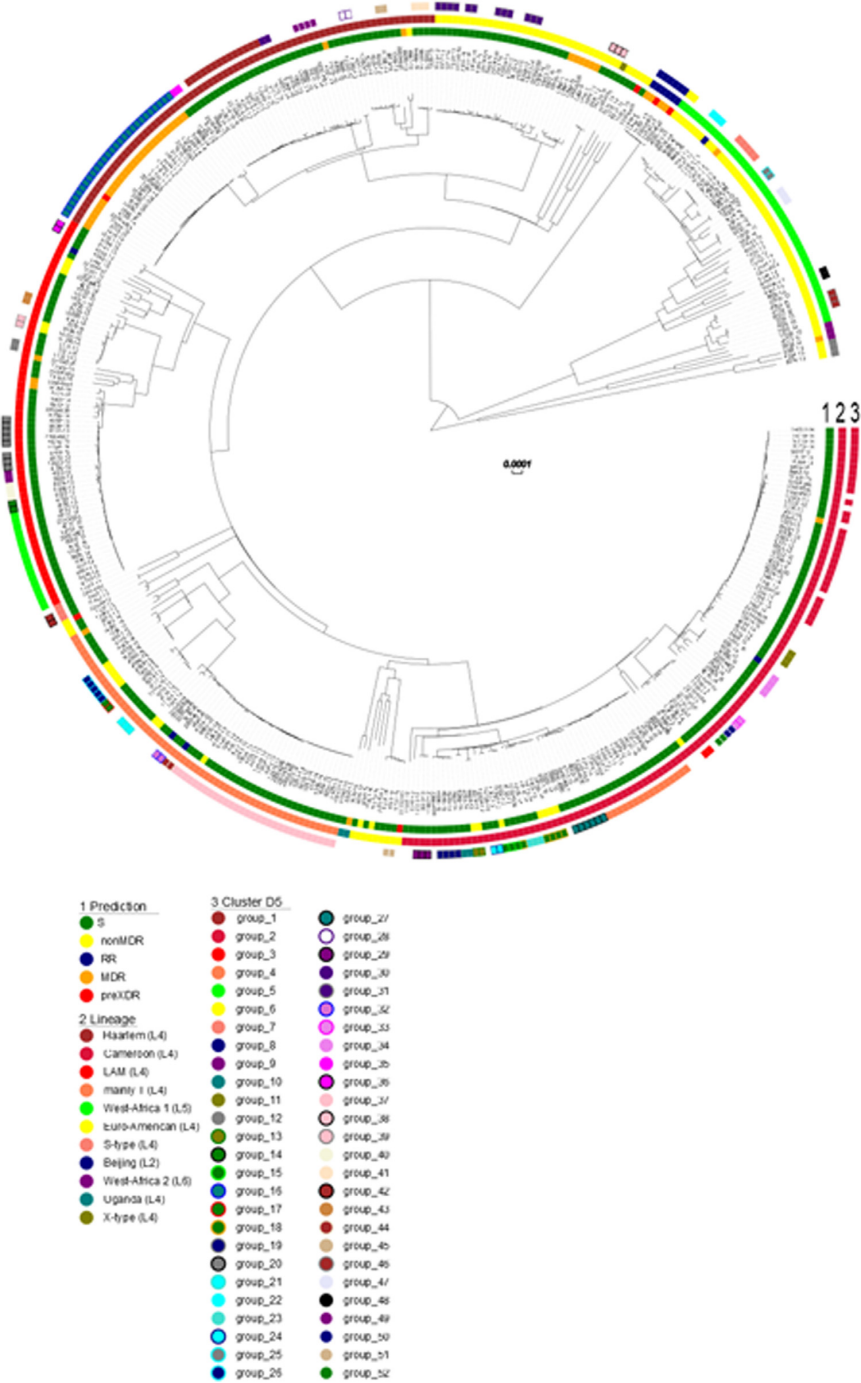
Maximum likelihood tree of 430 *Mycobacterium tuberculosis* complex strains, from Lambaréné Gabon collected from 2012 to 2022 at CERMEL, based on 15,225 variant sites, applying a generalized time-reversible substitution model. SNP, single nucleotide polymorphism. Legend: Inner to outer rim: 1: phylogenetic lineages; 2: genome-based grouping (pairwise distance <5 SNPs).

Based on WGS data, Mtbc strains were assigned to five previously defined phylogenetic lineages: L4 (n = 372; 86.5%), L5 (n = 46; 10.7%), L2 (n = 6; 1.4%), and L6 (n = 3; 0.7%) (Table 1, Supplement 1) based on lineage-specific SNP signatures. The majority of Mtbc strains belonged to lineage 4 (86.5%; 372 of 430) and were further subdivided into strains of Cameroon (27%; 116 of 430), LAM (15.8%; 68 of 430), Haarlem (17.2%; 74 of 430), T (13.5%; 58 of 430), Uganda I (0.5%; two of 430), X-type (0.2%; one of 430), S-type (0.7%; three of 430), and Euro-American not further defined (11.6%; 50 of 430) sublineages. In addition to strains of lineage 4, strains of lineage 2 (1.4%; six of 430) and *M. africanum* lineages L5 (10.7%; 46 of 430) and L6 (0.7%; three of 430) were found. Three strains (0.9%; three of 430) could not be assigned to a specific phylogenetic lineage and remained as *M. africanum, M. caprae, M. microti*, or *M. pinipedii* not further defined based on Homolka *et al.* [20]. A maximum likelihood phylogeny of the 430 Mtbc strains calculated using a concatenated list of 15,225 SNPs (Figure 1) fully confirmed the phylogenetic classification of the strains investigated.

### Resistance profile

The genotypic drug resistance profile was extracted from the WGS data. In total, 69.8% (300 of 430) of the strains showed no resistance mediating mutation and were classified as fully susceptible. Based on the WHO definitions of DR-TB types, 57 (13.3%) patients had at least RR-TB, of which 46 were further classified as MDR-TB (10.7%) and six as pre–XDR-TB (1.4% of all; 11% of the RR strains) due to additional FQ resistance. In addition, 17% (73 of 430) showed any resistance mediating mutation but were not RR/MDR and were classified as non–MDR-TB (Supplementary Table S2). Two strains had mutations in Rv0678, a gene that plays a role in bedaquiline/CFZ resistance. One had the known resistance-conferring mutation Rv0678 138_ins_g, and the other had the mutation Rv0678 A36T, which is not yet known to confer resistance and was detected in a strain isolated in 2015. No mutations conferring resistance to other group A drugs such as linezolid were observed.

### Cluster analysis

Clustering of strains based on a threshold of five SNPs revealed that 64.2% (276 of 430) were in the transmission networks. The close relationship of the strains in a cluster was confirmed in the Maximum Likelihood Tree (MLT) phylogeny (Figure 1). Of the 276 clustered strains, 75% (207/276) were genotypically susceptible; however, the majority of RR/MDR/pre-XDR strains (34 of 55, 62%) were also grouped in transmission chains (Figure 1).

Clusters were found in strains of all lineages, e.g. 91 of 116 Cameroon strains (78%), 56 of the 74 Haarlem strains (76%), 40 of 68 LAM strains (59%), and 44 of 58 mainly T strains (76%) are clustered, indicating ongoing recent transmission of strains of these lineages (Supplementary Table S2). We also found that in 20 of 46 West African 1 strain (44%) are clustered, whereas none of the three West African 2 strains were found in clusters. The cluster sizes ranged from two to a maximum of five strains (Supplementary Table S2).

The cluster size ranged from two to 30, with a median of three. The five largest d5 clusters comprised 28% (104 of 430, cluster sizes group_2 n = 30, group_37 n = 30, group_16 n = 27, group_5 n = 17, and group_4 n = 16 strains) of all strains in the collection. Notably, the largest clusters are formed by L4 strains (group_2 and group_4: Cameroon/L4.6.2.2, group_37: mainly T/L4.8, group_16: Haarlem/L4.1.2.1, group_5: LAM/L4.3.4.2). Particularly, the two largest clusters with 30 strains each were formed by group_2 strains belonging to lineage 4.6.2.2/Cameroon sub-lineage and were all fully susceptible, and group_37 strains belonging to lineage 4.8/mainly T sub-lineage, except one with a *katG* mutation, were also all fully susceptible (Figure 1, Supplementary Table 1).

**Figure 2:**
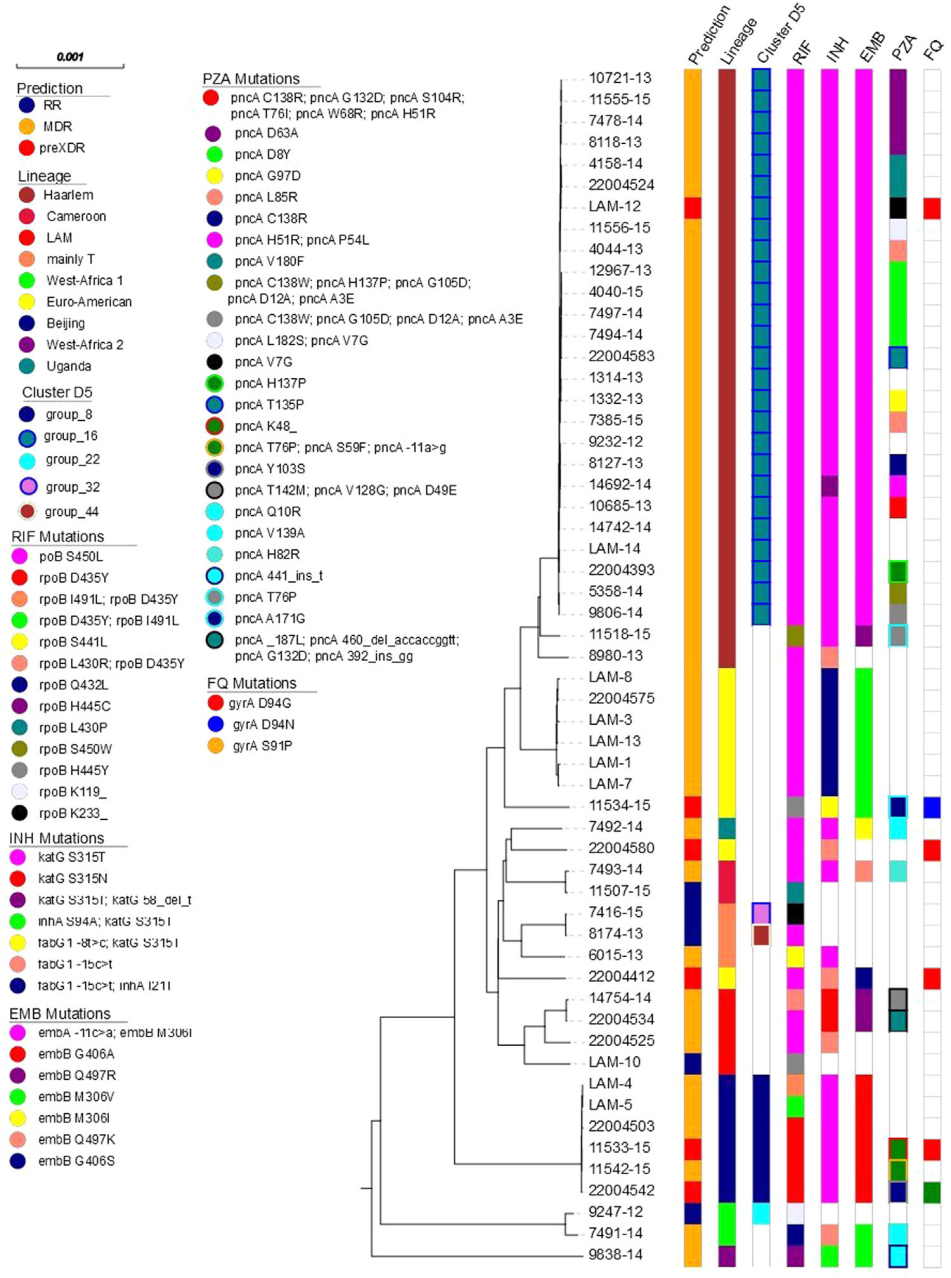
Maximum likelihood tree of 56 rifampicin-resistant *Mycobacterium tuberculosis* complex strains based on a concatenated SNP alignment with 3163 informative sites. D, distance; FQ, fluoroquinolones; EMB, ethambutol; INH, isoniazid; MDR, multi-drug resistant; PZA, pyrazinamide; RIF, rifampicin; RR, rifampicin resistant; XDR, Extensively drug-resistant. Legend: Next to the tree, different categories are color coded: 1. prediction, 2. phylogenetic lineage, 3. genome-based grouping (pairwise distance <5 SNPs), 4. rifampicin resistance-conferring mutations, 5. isoniazid resistance-conferring mutations, 6. ethambutol resistance-conferring mutations, 7. pyrazinamide resistance-conferring mutations, and 8. fluoroquinolone resistance-conferring mutations. Group_16 forms the largest cluster and is very clonal by sharing mainly the same resistance-conferring mutations for RIF, INH, and EMB, whereas there is a variety in PZA mutations and only one strain with a FQ resistance-conferring mutation.

Although the clusters formed by susceptible strains are formed by a large diversity of clusters with strains from different lineages (Supplementary Table 2), the clusters of MDR/pre-XDR strains are more clonal and mainly formed by two outbreak strains, namely, cluster group_16 (26 MDR and one pre-XDR strain) and group_8 (four MDR and two pre-XDR strains). This shows the presence of a dominant MDR/pre-XDR strain, d5 group_16, that comprised 27 strains classified as lineage 4.1.2.1 (Haarlem sub-lineage) and representing 57% of all MDR strains (26 of 46) and 87% (26 of 30) of all MDR-TB strains in potential transmission networks (Figure 2). The clonality of the strains of the largest MDR/pre-XDR cluster can be seen in the MLT calculated for all RR/MDR/pre-XDR Mtbc strains (Figure 2).

The strains of this cluster are characterized by isoniazid resistance-conferring mutation *katG* S315T, RIF resistance-conferring mutation *rpoB* S450L, EMB resistance-conferring mutations *embA* -11c>a, *embB* M306I, and ETH/PTH resistance-conferring mutation *ethA* A341V. In addition, all strains of this cluster have compensatory mutations *rpoC* G594E and *rpoC* P1040Q. The distribution of pyrazinamide mutations varied; four strains were susceptible, whereas the others had nearly all unique resistance mutations (Figure 2, Supplementary Table 2). The most prominent mutation was *pncA* D63A, with four strains having this mutation. Between 2012 and 2018, at least one strain of this cluster was collected in each year. One pre-XDR strain was obtained in 2018 and had an additional FQ resistance-conferring mutation (gyrA D94G).

## Discussion

Using a molecular epidemiological approach based on WGS of clinical Mtbc strains, we gathered the first data on the population structure and transmission dynamics of Mtbc strains in Gabon over a study period of 10 years. Our findings demonstrated a predominance of L4 (87%) strains, with significant diversity at the sub-lineage level. Overall, 57 Mtbc strains showed any RR and 46 were MDR, of which 11% had an additional FQ resistance and were classified as pre-XDR. The cluster data obtained based on a strict SNP threshold indicate a high rate of transmission for susceptible and resistant Mtbc strains. Lineage 4 strains form large transmission networks spanning the whole study period. Particularly alarming is the detection of an MDR/pre-XDR outbreak strain, representing 27 of the 52 MDR/pre-XDR strains (52%) in the study population, which was detected over the whole study period.

Our results show a predominance of L4 (87%) Mtbc strains in the study population, with a significant population diversity with strains belonging to more than six sub-lineages. This is consistent with other studies using WGS for Mtbc strain characterization in Africa [21]. A high proportion (87%) of L4 strain types observed in our cohort have indeed been reported in many low- and middle-income countries [22], confirming the capacity of L4 strains to efficiently spread in African settings. The dispersal of L4 strains in different African countries may reflect historical migratory events during the colonization that potentially explain the large dispersion of this lineage and reflect its adaptation to different human populations [23]. The dominance of strains belonging to the Cameroon sub-lineage is in line with data from other West African countries, such as the neighboring country Cameroon [24]. Our data add to the general hypothesis that strains of several L4 sub-lineages may indeed be classified as generalists, which can spread in different geographical regions due to their capacity to spread in different host populations.

Interestingly, *M. africanum* strains (L5 and L6) still accounted for more than 10% of the Mtbc strains in Gabon. This is higher than what has been reported in the neighboring country (Cameroon) [25]. Quite a number of the Gabonese population is of West African origin and most travel very often from Gabon to visit their home country. This could explain the proportion of *M. africanum* strains in our cohort. To date, we found a large proportion (44%) of *M. africanum* West African 1 (L5) strains belonging to a cluster; however, the clusters were small, with the largest cluster containing five strains. This indicates the introduction of several different strains into the country and against one clone spreading in the area. There is a need for further research on the TB transmission dynamics to further investigate if the *M. africanum* strain is local or imported.

The low proportion of L2 Mtbc strains supports previous reports from some African countries [26]. The strains of L2 are frequently observed in Asia, and one possible explanation for the low proportion observed in Gabon and other African countries is that it has recently been imported and the dispersion has been limited. Nevertheless, monitoring changes in the prevalence of strains of particular lineages, such as L2 strains, in geographical regions, for example, using molecular surveillance, is important because it has been reported to be associated with high virulence, rapid spread, and drug resistance [19].

A total of 13.2% of the strains sequenced were RR. This is more than the previous report in Lambaréné [27]. This proportion of RR-TB strains is similar to what was reported in southern Ethiopia [28]. However, even more worrisome, we found that 11% of the RR Mtbc strains already had an additional FQ resistance and were classified as pre-XDR. This is an alarm signal, although the prevalence of FQ resistance among RR Mtbc strains is still below 23% among MDR strains from Mozambique, which was recently reported [29].

Particularly alarming is detecting an MDR/pre-XDR outbreak strain, representing 27 of the 52 MDR/pre-XDR strains (52%). This indicates that the ongoing transmission of one MDR/pre-XDR is the main driver of the MDR-TB epidemic in the country. Similar findings have been reported from South Africa or Mozambique, where a high rate of clustering, and the presence of dominant longitudinally spreading clones are characteristics of the MDR/pre-XDR/XDR epidemiology [26,29]. This indicates that the genetic background, including the compensatory mutations found, provides this MDR strain a high capacity to spread, in line with reports from other settings such as Africa or Eastern Europe [29].

High cluster rates have also been found in susceptible strains, mainly linked with L4 strains and several outbreak clones, spanning a decade. As such, ongoing recent transmission is an important driver of the TB epidemic in the country for susceptible and resistant TB. Reasons for this are manifold and range from delays in case detection and proper diagnostics to initiation of effective regimens.

### Limitations

It is important to note that there was a discontinuation in data collection in 2018 due to logistic and sample processing before the shipment to Bostel. This led to a selection bias, and the data collected are not representative of the full study period. Due to the infrastructure, we were not able to perform the phenotypical drug susceptibility test and, therefore, we could not ensure the concordance of molecular resistance and phenotypic resistance.

## Conclusion

To the best of our knowledge, in this study, we provide, for the first time, using WGS, the genetic diversity, drug resistance profiles, and transmission dynamics of Mtbc strains circulating in Gabon. We found that the TB epidemic in the country is driven by a high transmission mainly of strains from L4, still with a high diversity of sub-lineages. Levels of pre-XDR among RR strains reached an alarming level. Overall, the drug-resistant TB epidemic in the country is mainly driven by one dominant MDR L4 Haarlem strain. The data indicate the urgent need for effective public health intervention to stop the ongoing transmission, especially of MDR Mtbc strains. Considering the limitations of our study, establishing prospective molecular surveillance at the national level in the country is key to monitoring transmission dynamics across the country and resistance evolution with a focus on new group A MDR-TB treatment regimens, such as bedaquiline.

## Declarations of competing interest

The authors have no competing interest to declare.

## Appendix Supplementary materials

Supplementary Table S1. Mutations (small deletions and SNPs) in resistance-associated target regions. Image, application 1

Supplementary Table S2. Cluster by lineage. Image, application 2
