# Supplementary material for: Clinical epidemiology, genetic diversity, and drug susceptibility patterns by whole genome sequencing of Mycobacterium tuberculosis complex isolates in Gabon from 2012 to 2022
Source: IJID Reg. 2024 Nov 28;14:100501. doi: 10.1016/j.ijregi.2024.100501 (PMC11718294; doi:10.1016/j.ijregi.2024.100501)
Supplement: Supplementary file 1 — Supplementary Table S1. Mutations (small deletions and SNPs) in resistance-associated target regions. [file mmc1.pdf]

genome	eference_allel	nutated_allel	TYPE	AFFECTS	gene_number	coding_strand	gene	UTATION_annotati	PRED_drug	EVIDENCE	comment	modified	curator	gene_mutation	PRED_WHO	WHO_tier	nrIleOAsn_AlaAsnA	WHO nomenclature
3987171	AC	A	INDEL	PROM	Rv3547	forward	ddn	A111fs	DLM-R	WHO2023		14.12.2023	VD	ddn_A111fs	2) Assoc w R - Interim	2	Ala111fs	ddn_c.Ala111fs
3987163	TG	T	INDEL	PROM	Rv3547	forward	ddn	D108fs	DLM-R	WHO2023		14.12.2023	VD	ddn_D108fs	2) Assoc w R - Interim	2	Asp108fs	ddn_c.Asp108fs
3986953	TG	T	INDEL	PROM	Rv3547	forward	ddn	G38fs	DLM-R	WHO2023		14.12.2023	VD	ddn_G38fs	2) Assoc w R - Interim	2	Gly38fs	ddn_c.Gly38fs
3987149	CA	C	INDEL	PROM	Rv3547	forward	ddn	K104fs	DLM-R	WHO2023		14.12.2023	VD	ddn_K104fs	2) Assoc w R - Interim	2	Lys104fs	ddn_c.Lys104fs
3987028	A	AC	INDEL	PROM	Rv3547	forward	ddn	L64fs	DLM-R	WHO2023		14.12.2023	VD	ddn_L64fs	2) Assoc w R - Interim	2	Leu64fs	ddn_c.Leu64fs
3987109	AC	A	INDEL	PROM	Rv3547	forward	ddn	L90fs	DLM-R	WHO2023		14.12.2023	VD	ddn_L90fs	2) Assoc w R - Interim	2	Leu90fs	ddn_c.Leu90fs
3986737	TAATACCTGG/	G	INDEL	PROM	Rv3547	forward	ddn	LoF	DLM-R	WHO2023		14.12.2023	VD	ddn_LoF	2) Assoc w R - Interim	2	LoF	ddn_c.LoF
3986925	AT	A	INDEL	PROM	Rv3547	forward	ddn	M28fs	DLM-R	WHO2023		14.12.2023	VD	ddn_M28fs	2) Assoc w R - Interim	2	Met28fs	ddn_c.Met28fs
3986849	G	GAAATC	INDEL	PROM	Rv3547	forward	ddn	P5fs	DLM-R	WHO2023		14.12.2023	VD	ddn_P5fs	2) Assoc w R - Interim	2	Pro5fs	ddn_c.Pro5fs
3986932	GC	G	INDEL	PROM	Rv3547	forward	ddn	R31fs	DLM-R	WHO2023		14.12.2023	VD	ddn_R31fs	2) Assoc w R - Interim	2	Arg31fs	ddn_c.Arg31fs
3986902	G	A	SNP	PROM	Rv3547	forward	ddn	W20_	DLM-R	WHO2023		14.12.2023	VD	ddn_W20_	2) Assoc w R - Interim	2	Trp20*	ddn_c.Trp20*
2715344	G	A	SNP	PROM	Rv2416c	reverse	eis	-12c>t	KAN-R	WHO2018	low MIC increase (WHO)	14.12.2023	VD	eis_-12c>t	1) Assoc w R	#NV	-12c>t	eis_c.-12c>t
2715346	G	A	SNP	PROM	Rv2416c	reverse	eis	-14c>t	KAN-R,AMI-R, CAP-R	WHO2018	low AMI MIC increase, moderate KAN MIC increase (WHO)	14.12.2023	VD	eis_-14c>t	1) Assoc w R	#NV	-14c>t	eis_c.-14c>t
2715339	CG	C	INDEL	CDS	Rv2416c	reverse	eis	-8delC	KAN-R	WHO2023		14.12.2023	VD	eis_-8delC	2) Assoc w R - Interim	2	-8delC	eis_p.-8delC
4326163	GAAGTGCTTT(	A	INDEL	CDS	Rv3854c	reverse	ethA	A128fs	ETH-R	WHO2023		14.12.2023	VD	ethA_A128fs	2) Assoc w R - Interim	2	Ala128fs	ethA_p.Ala128fs
4326528	CTGTACACGG(	T	INDEL	CDS	Rv3854c	reverse	ethA	A185fs	ETH-R	WHO2023		14.12.2023	VD	ethA_A185fs	2) Assoc w R - Interim	2	Ala185fs	ethA_p.Ala185fs
4326914	GC	G	INDEL	CDS	Rv3854c	reverse	ethA	A187fs	ETH-R	WHO2023		14.12.2023	VD	ethA_A187fs	2) Assoc w R - Interim	2	Ala187fs	ethA_p.Ala187fs
4327091	.TCGCCTGCCG	G	INDEL	CDS	Rv3854c	reverse	ethA	A20fs	ETH-R	WHO2023		14.12.2023	VD	ethA_A20fs	2) Assoc w R - Interim	2	Ala20fs	ethA_p.Ala20fs
4326765	C	CG	INDEL	CDS	Rv3854c	reverse	ethA	A237fs	ETH-R	WHO2023		14.12.2023	VD	ethA_A237fs	2) Assoc w R - Interim	2	Ala237fs	ethA_p.Ala237fs
4326734	G	GCCTGGCCC	INDEL	CDS	Rv3854c	reverse	ethA	A247fs	ETH-R	WHO2023		14.12.2023	VD	ethA_A247fs	2) Assoc w R - Interim	2	Ala247fs	ethA_p.Ala247fs
4326721	CTGTACACGG	G	INDEL	CDS	Rv3854c	reverse	ethA	A248fs	ETH-R	WHO2023		14.12.2023	VD	ethA_A248fs	2) Assoc w R - Interim	2	Ala248fs	ethA_p.Ala248fs
4326419	GC	G	INDEL	CDS	Rv3854c	reverse	ethA	A352fs	ETH-R	WHO2023		14.12.2023	VD	ethA_A352fs	2) Assoc w R - Interim	2	Ala352fs	ethA_p.Ala352fs
4326075	TCGTCCATGT/	G	INDEL	CDS	Rv3854c	reverse	ethA	A354fs	ETH-R	WHO2023		14.12.2023	VD	ethA_A354fs	2) Assoc w R - Interim	2	Ala354fs	ethA_p.Ala354fs
4326290	G	GC	INDEL	CDS	Rv3854c	reverse	ethA	A395fs	ETH-R	WHO2023		14.12.2023	VD	ethA_A395fs	2) Assoc w R - Interim	2	Ala395fs	ethA_p.Ala395fs
4326155	CTGGATCAGG(	A	INDEL	CDS	Rv3854c	reverse	ethA	A90fs	ETH-R	WHO2023		14.12.2023	VD	ethA_A90fs	2) Assoc w R - Interim	2	Ala90fs	ethA_p.Ala90fs
4326304	CATGCGCCGT(	G	INDEL	CDS	Rv3854c	reverse	ethA	C131_	ETH-R	WHO2023		14.12.2023	VD	ethA_C131_	2) Assoc w R - Interim	2	Cys131*	ethA_p.Cys131*
4326304	CATGCGCCGT(	G	INDEL	CDS	Rv3854c	reverse	ethA	C131fs	ETH-R	WHO2023		14.12.2023	VD	ethA_C131fs	2) Assoc w R - Interim	2	Cys131fs	ethA_p.Cys131fs
4326715	GC	G	INDEL	CDS	Rv3854c	reverse	ethA	C253fs	ETH-R	WHO2023		14.12.2023	VD	ethA_C253fs	2) Assoc w R - Interim	2	Cys253fs	ethA_p.Cys253fs
4326810	GTGCCGTGGC	C	INDEL	CDS	Rv3854c	reverse	ethA	C27fs	ETH-R	WHO2023		14.12.2023	VD	ethA_C27fs	2) Assoc w R - Interim	2	Cys27fs	ethA_p.Cys27fs
4326119	AAGCATCATG	C	INDEL	CDS	Rv3854c	reverse	ethA	C294fs	ETH-R	WHO2023		14.12.2023	VD	ethA_C294fs	2) Assoc w R - Interim	2	Cys294fs	ethA_p.Cys294fs
4326265	AC	GT	SNP	CDS	Rv3854c	reverse	ethA	C403Y	ETH-R	WHO2023		14.12.2023	VD	ethA_C403Y	2) Assoc w R - Interim	2	Cys403Tyr	ethA_p.Cys403Tyr
4326347	GGGTAGCTGG(	C	INDEL	CDS	Rv3854c	reverse	ethA	D170fs	ETH-R	WHO2023		14.12.2023	VD	ethA_D170fs	2) Assoc w R - Interim	2	Asp170fs	ethA_p.Asp170fs
4326883	CGAGT	C	INDEL	CDS	Rv3854c	reverse	ethA	D196fs	ETH-R	WHO2023		14.12.2023	VD	ethA_D196fs	2) Assoc w R - Interim	2	Asp196fs	ethA_p.Asp196fs
4326815	CCG	C	INDEL	CDS	Rv3854c	reverse	ethA	D219fs	ETH-R	WHO2023		14.12.2023	VD	ethA_D219fs	2) Assoc w R - Interim	2	Asp219fs	ethA_p.Asp219fs
4326604	GTC	TT	INDEL	CDS	Rv3854c	reverse	ethA	D290fs	ETH-R	WHO2023		14.12.2023	VD	ethA_D290fs	2) Assoc w R - Interim	2	Asp290fs	ethA_p.Asp290fs
4326529	G	GGCGGCGTTG	INDEL	CDS	Rv3854c	reverse	ethA	D315fs	ETH-R	WHO2023		14.12.2023	VD	ethA_D315fs	2) Assoc w R - Interim	2	Asp315fs	ethA_p.Asp315fs
4326388	GT	G	INDEL	CDS	Rv3854c	reverse	ethA	D362fs	ETH-R	WHO2023		14.12.2023	VD	ethA_D362fs	2) Assoc w R - Interim	2	Asp362fs	ethA_p.Asp362fs
4326286	GT	G	INDEL	CDS	Rv3854c	reverse	ethA	D396fs	ETH-R	WHO2023		14.12.2023	VD	ethA_D396fs	2) Assoc w R - Interim	2	Asp396fs	ethA_p.Asp396fs
4326243	CG	C	INDEL	CDS	Rv3854c	reverse	ethA	D410fs	ETH-R	WHO2023		14.12.2023	VD	ethA_D410fs	2) Assoc w R - Interim	2	Asp410fs	ethA_p.Asp410fs
4326230	T	TC	INDEL	CDS	Rv3854c	reverse	ethA	D415fs	ETH-R	WHO2023		14.12.2023	VD	ethA_D415fs	2) Assoc w R - Interim	2	Asp415fs	ethA_p.Asp415fs
4326078	GGATGT	G	INDEL	CDS	Rv3854c	reverse	ethA	D464fs	ETH-R	WHO2023		14.12.2023	VD	ethA_D464fs	2) Assoc w R - Interim	2	Asp464fs	ethA_p.Asp464fs
4327318	GATAACGGAA(	T	INDEL	CDS	Rv3854c	reverse	ethA	D46fs	ETH-R	WHO2023		14.12.2023	VD	ethA_D46fs	2) Assoc w R - Interim	2	Asp46fs	ethA_p.Asp46fs
4327242	CG	C	INDEL	CDS	Rv3854c	reverse	ethA	D77fs	ETH-R	WHO2023		14.12.2023	VD	ethA_D77fs	2) Assoc w R - Interim	2	Asp77fs	ethA_p.Asp77fs
4327189	G	GGCGAT	INDEL	CDS	Rv3854c	reverse	ethA	D95fs	ETH-R	WHO2023		14.12.2023	VD	ethA_D95fs	2) Assoc w R - Interim	2	Asp95fs	ethA_p.Asp95fs
4327078	TTC	CTA	SNP	CDS	Rv3854c	reverse	ethA	E132_	ETH-R	WHO2023		14.12.2023	VD	ethA_E132_	2) Assoc w R - Interim	2	Glu132*	ethA_p.Glu132*
4327009	CTC	TCA	SNP	CDS	Rv3854c	reverse	ethA	E155_	ETH-R	WHO2023		14.12.2023	VD	ethA_E155_	2) Assoc w R - Interim	2	Glu155*	ethA_p.Glu155*
4326969	CG	C	INDEL	CDS	Rv3854c	reverse	ethA	E169fs	ETH-R	WHO2023		14.12.2023	VD	ethA_E169fs	2) Assoc w R - Interim	2	Glu169fs	ethA_p.Glu169fs
4326801	GCTTC	G	INDEL	CDS	Rv3854c	reverse	ethA	E223fs	ETH-R	WHO2023		14.12.2023	VD	ethA_E223fs	2) Assoc w R - Interim	2	Glu223fs	ethA_p.Glu223fs
4326268	TGATGATGTC/	G	INDEL	CDS	Rv3854c	reverse	ethA	E274fs	ETH-R	WHO2023		14.12.2023	VD	ethA_E274fs	2) Assoc w R - Interim	2	Glu274fs	ethA_p.Glu274fs
4326515	TGGTGTCGGT(	A	INDEL	CDS	Rv3854c	reverse	ethA	E311fs	ETH-R	WHO2023		14.12.2023	VD	ethA_E311fs	2) Assoc w R - Interim	2	Glu311fs	ethA_p.Glu311fs
4326114	GTGTAGCCAA	G	INDEL	CDS	Rv3854c	reverse	ethA	E318fs	ETH-R	WHO2023		14.12.2023	VD	ethA_E318fs	2) Assoc w R - Interim	2	Glu318fs	ethA_p.Glu318fs
4326478	TTC	CTA	SNP	CDS	Rv3854c	reverse	ethA	E332_	ETH-R	WHO2023		14.12.2023	VD	ethA_E332_	2) Assoc w R - Interim	2	Glu332*	ethA_p.Glu332*
4326358	ATCGTGACGT(	C	INDEL	CDS	Rv3854c	reverse	ethA	E36fs	ETH-R	WHO2023		14.12.2023	VD	ethA_E36fs	2) Assoc w R - Interim	2	Glu36fs	ethA_p.Glu36fs
4326850	GTGCCGTGGCT	T	INDEL	CDS	Rv3854c	reverse	ethA	E39fs	ETH-R	WHO2023		14.12.2023	VD	ethA_E39fs	2) Assoc w R - Interim	2	Glu39fs	ethA_p.Glu39fs
4327465	CT	C	INDEL	CDS	Rv3854c	reverse	ethA	E3fs	ETH-R	WHO2023		14.12.2023	VD	ethA_E3fs	2) Assoc w R - Interim	2	Glu3fs	ethA_p.Glu3fs
4326265	ACAGACAAACT	A	INDEL	CDS	Rv3854c	reverse	ethA	E400fs	ETH-R	WHO2023		14.12.2023	VD	ethA_E400fs	2) Assoc w R - Interim	2	Glu400fs	ethA_p.Glu400fs
4326193	TTC	CTA	SNP	CDS	Rv3854c	reverse	ethA	E427_	ETH-R	WHO2023		14.12.2023	VD	ethA_E427_	2) Assoc w R - Interim	2	Glu427*	ethA_p.Glu427*
4326189	GCT	G	INDEL	CDS	Rv3854c	reverse	ethA	E428fs	ETH-R	WHO2023		14.12.2023	VD	ethA_E428fs	2) Assoc w R - Interim	2	Glu428fs	ethA_p.Glu428fs
4326140	T	TC	INDEL	CDS	Rv3854c	reverse	ethA	E445fs	ETH-R	WHO2023		14.12.2023	VD	ethA_E445fs	2) Assoc w R - Interim	2	Glu445fs	ethA_p.Glu445fs
4327206	GTGCTCTTGA(	C	INDEL	CDS	Rv3854c	reverse	ethA	E83fs	ETH-R	WHO2023		14.12.2023	VD	ethA_E83fs	2) Assoc w R - Interim	2	Glu83fs	ethA_p.Glu83fs
4327174	GA	G	INDEL	CDS	Rv3854c	reverse	ethA	F100fs	ETH-R	WHO2023		14.12.2023	VD	ethA_F100fs	2) Assoc w R - Interim	2	Phe100fs	ethA_p.Phe100fs
4326803	TGACCGCCGT	T	INDEL	CDS	Rv3854c	reverse	ethA	F151fs	ETH-R	WHO2023		14.12.2023	VD	ethA_F151fs	2) Assoc w R - Interim	2	Phe151fs	ethA_p.Phe151fs
4326224	CCAAAAAGCT(	A	INDEL	CDS	Rv3854c	reverse	ethA	F282fs	ETH-R	WHO2023		14.12.2023	VD	ethA_F282fs	2) Assoc w R - Interim	2	Phe282fs	ethA_p.Phe282fs
4326041	AAAACCGTTG	A	INDEL	CDS	Rv3854c	reverse	ethA	F349fs	ETH-R	WHO2023		14.12.2023	VD	ethA_F349fs	2) Assoc w R - Interim	2	Phe349fs	ethA_p.Phe349fs
4326230	TCA	T	INDEL	CDS	Rv3854c	reverse	ethA	F414fs	ETH-R	WHO2023		14.12.2023	VD	ethA_F414fs	2) Assoc w R - Interim	2	Phe414fs	ethA_p.Phe414fs
4326180	TGA	T	INDEL	CDS	Rv3854c	reverse	ethA	F431fs	ETH-R	WHO2023		14.12.2023	VD	ethA_F431fs	2) Assoc w R - Interim	2	Phe431fs	ethA_p.Phe431fs
4326172	GA	G	INDEL	CDS	Rv3854c	reverse	ethA	F434fs	ETH-R	WHO2023		14.12.2023	VD	ethA_F434fs	2) Assoc w R - Interim	2	Phe434fs	ethA_p.Phe434fs
4326705	ATCGGCCCGA	G	INDEL	CDS	Rv3854c	reverse	ethA	F64fs	ETH-R	WHO2023		14.12.2023	VD	ethA_F64fs	2) Assoc w R - Interim	2	Phe64fs	ethA_p.Phe64fs
4327441	GC	G	INDEL	CDS	Rv3854c	reverse	ethA	G11fs	ETH-R	WHO2023		14.12.2023	VD	ethA_G11fs	2) Assoc w R - Interim	2	Gly11fs	ethA_p.Gly11fs
4326650	C	CT	INDEL	CDS	Rv3854c	reverse	ethA	G275fs	ETH-R	WHO2023		14.12.2023	VD	ethA_G275fs	2) Assoc w R - Interim	2	Gly275fs	ethA_p.Gly275fs
4326300	GATGATGTCA(	T	INDEL	CDS	Rv3854c	reverse	ethA	G283fs	ETH-R	WHO2023		14.12.2023	VD	ethA_G283fs	2) Assoc w R - Interim	2	Gly283fs	ethA_p.Gly283fs
4326577	GC	G	INDEL	CDS	Rv3854c	reverse	ethA	G299fs	ETH-R	WHO2023		14.12.2023	VD	ethA_G299fs	2) Assoc w R - Interim	2	Gly299fs	ethA_p.Gly299fs
4326424	AC	A	INDEL	CDS	Rv3854c	reverse	ethA	G350fs	ETH-R	WHO2023		14.12.2023	VD	ethA_G350fs	2) Assoc w R - Interim	2	Gly350fs	ethA_p.Gly350fs
4326361	GC	G	INDEL	CDS	Rv3854c	reverse	ethA	G371fs	ETH-R	WHO2023		14.12.2023	VD	ethA_G371fs	2) Assoc w R - Interim	2	Gly371fs	ethA_p.Gly371fs
4326321	C	CA	INDEL	CDS	Rv3854c	reverse	ethA	G385fs	ETH-R	WHO2023		14.12.2023	VD	ethA_G385fs	2) Assoc w R - Interim	2	Gly385fs	ethA_p.Gly385fs
4326203	GAGCC	G	INDEL	CDS	Rv3854c	reverse	ethA	G423fs	ETH-R	WHO2023		14.12.2023	VD	ethA_G423fs	2) Assoc w R - Interim	2	Gly423fs	ethA_p.Gly423fs
4327345	GCCGC	G	INDEL	CDS	Rv3854c	reverse	ethA	G42fs	ETH-R	WHO2023		14.12.2023	VD	ethA_G42fs	2) Assoc w R - Interim	2	Gly42fs	ethA_p.Gly42fs
4326166	T	TG	INDEL	CDS	Rv3854c	reverse	ethA	G437fs	ETH-R	WHO2023		14.12.2023	VD	ethA_G437fs	2) Assoc w R - Interim	2	Gly437fs	ethA_p.Gly437fs
4326621	CGGATGGATG/	G	INDEL	CDS	Rv3854c	reverse	ethA	G43fs	ETH-R	WHO2023		14.12.2023	VD	ethA_G43fs	2) Assoc w R - Interim	2	Gly43fs	ethA_p.Gly43fs
4326034	GAACCGCAGA(	G	INDEL	CDS	Rv3854c	reverse	ethA	G477fs	ETH-R	WHO2023		14.12.2023	VD	ethA_G477fs	2) Assoc w R - Interim	2	Gly477fs	ethA_p.Gly477fs
4326850	GCGAATCTCGG	T	INDEL	CDS	Rv3854c	reverse	ethA	G93fs	ETH-R	WHO2023		14.12.2023	VD	ethA_G93fs	2) Assoc w R - Interim	2	Gly93fs	ethA_p.Gly93fs
4326801	GGATGGATGAT	G	INDEL	CDS	Rv3854c	reverse	ethA	H101fs	ETH-R	WHO2023		14.12.2023	VD	ethA_H101fs	2) Assoc w R - Interim	2	His101fs	ethA_p.His101fs
4326675	CGCCGGCACG/	G	INDEL	CDS	Rv3854c	reverse	ethA	H119fs	ETH-R	WHO2023		14.12.2023	VD	ethA_H119fs	2) Assoc w R - Interim	2	His119fs	ethA_p.His119fs
4326942	TCGAGGTCCTC	T	INDEL	CDS	Rv3854c	reverse	ethA	H163fs	ETH-R	WHO2023		14.12.2023	VD	ethA_H163fs	2) Assoc w R - Interim	2	His163fs	ethA_p.His163fs

4327408	G	GT	INDEL	CDS	Rv3854c	reverse	ethA	H22fs	ETH-R	WHO2023	14.12.2023 VD	ethA_H22fs	2) Assoc w R - Interim	2	His22fs	ethA_p.His22fs
4325972	TCCAGGAGG	T	INDEL	CDS	Rv3854c	reverse	ethA	H281fs	ETH-R	WHO2023	14.12.2023 VD	ethA_H281fs	2) Assoc w R - Interim	2	His281fs	ethA_p.His281fs
4326620	T	TGC	INDEL	CDS	Rv3854c	reverse	ethA	H285fs	ETH-R	WHO2023	14.12.2023 VD	ethA_H285fs	2) Assoc w R - Interim	2	His285fs	ethA_p.His285fs
4326456	CGAGGTCCTC	T	INDEL	CDS	Rv3854c	reverse	ethA	H4fs	ETH-R	WHO2023	14.12.2023 VD	ethA_H4fs	2) Assoc w R - Interim	2	His4fs	ethA_p.His4fs
4327157	TTGTGGTGGA	C	INDEL	CDS	Rv3854c	reverse	ethA	H97fs	ETH-R	WHO2023	14.12.2023 VD	ethA_H97fs	2) Assoc w R - Interim	2	His97fs	ethA_p.His97fs
4326186	AAGCACAATC	G	INDEL	CDS	Rv3854c	reverse	ethA	I161fs	ETH-R	WHO2023	14.12.2023 VD	ethA_I161fs	2) Assoc w R - Interim	2	Ile161fs	ethA_p.Ile161fs
4326941	A	ACC	INDEL	CDS	Rv3854c	reverse	ethA	I178fs	ETH-R	WHO2023	14.12.2023 VD	ethA_I178fs	2) Assoc w R - Interim	2	Ile178fs	ethA_p.Ile178fs
4326189	GGCCCGGAA	G	INDEL	CDS	Rv3854c	reverse	ethA	I181fs	ETH-R	WHO2023	14.12.2023 VD	ethA_I181fs	2) Assoc w R - Interim	2	Ile181fs	ethA_p.Ile181fs
4326375	AGCACAATCG	T	INDEL	CDS	Rv3854c	reverse	ethA	I221fs	ETH-R	WHO2023	14.12.2023 VD	ethA_I221fs	2) Assoc w R - Interim	2	Ile221fs	ethA_p.Ile221fs
4325999	CCATGTAATTC	T	INDEL	CDS	Rv3854c	reverse	ethA	I325fs	ETH-R	WHO2023	14.12.2023 VD	ethA_I325fs	2) Assoc w R - Interim	2	Ile325fs	ethA_p.Ile325fs
4326021	TTGTCGTCCA	C	INDEL	CDS	Rv3854c	reverse	ethA	I337fs	ETH-R	WHO2023	14.12.2023 VD	ethA_I337fs	2) Assoc w R - Interim	2	Ile337fs	ethA_p.Ile337fs
4326064	ACAAGCGACA	G	INDEL	CDS	Rv3854c	reverse	ethA	I339fs	ETH-R	WHO2023	14.12.2023 VD	ethA_I339fs	2) Assoc w R - Interim	2	Ile339fs	ethA_p.Ile339fs
4326280	CCGTACCGCG	C	INDEL	CDS	Rv3854c	reverse	ethA	I81fs	ETH-R	WHO2023	14.12.2023 VD	ethA_I81fs	2) Assoc w R - Interim	2	Ile81fs	ethA_p.Ile81fs
4327105	GACCAATCGG	G	INDEL	CDS	Rv3854c	reverse	ethA	I94fs	ETH-R	WHO2023	14.12.2023 VD	ethA_I94fs	2) Assoc w R - Interim	2	Ile94fs	ethA_p.Ile94fs
4326939	CGATTCCATAC	C	INDEL	CDS	Rv3854c	reverse	ethA	I9fs	ETH-R	WHO2023	14.12.2023 VD	ethA_I9fs	2) Assoc w R - Interim	2	Ile9fs	ethA_p.Ile9fs
4327142	AATCGGCACT	G	INDEL	CDS	Rv3854c	reverse	ethA	K103_	ETH-R	WHO2023	14.12.2023 VD	ethA_K103_	2) Assoc w R - Interim	2	Lys103*	ethA_p.Lys103*
4327142	AATCGGCACT	G	INDEL	CDS	Rv3854c	reverse	ethA	K103fs	ETH-R	WHO2023	14.12.2023 VD	ethA_K103fs	2) Assoc w R - Interim	2	Lys103fs	ethA_p.Lys103fs
4326531	CCGTGGCCAC	C	INDEL	CDS	Rv3854c	reverse	ethA	K200fs	ETH-R	WHO2023	14.12.2023 VD	ethA_K200fs	2) Assoc w R - Interim	2	Lys200fs	ethA_p.Lys200fs
4326802	C	CCAGG	INDEL	CDS	Rv3854c	reverse	ethA	K224fs	ETH-R	WHO2023	14.12.2023 VD	ethA_K224fs	2) Assoc w R - Interim	2	Lys224fs	ethA_p.Lys224fs
4326751	CTT	TCA	SNP	CDS	Rv3854c	reverse	ethA	K241_	ETH-R	WHO2023	14.12.2023 VD	ethA_K241_	2) Assoc w R - Interim	2	Lys241*	ethA_p.Lys241*
4326751	CT	C	INDEL	CDS	Rv3854c	reverse	ethA	K241fs	ETH-R	WHO2023	14.12.2023 VD	ethA_K241fs	2) Assoc w R - Interim	2	Lys241fs	ethA_p.Lys241fs
4326228	AAGCTGCAGG	T	INDEL	CDS	Rv3854c	reverse	ethA	K280fs	ETH-R	WHO2023	14.12.2023 VD	ethA_K280fs	2) Assoc w R - Interim	2	Lys280fs	ethA_p.Lys280fs
4326145	GATGCCGGAA	C	INDEL	CDS	Rv3854c	reverse	ethA	K309fs	ETH-R	WHO2023	14.12.2023 VD	ethA_K309fs	2) Assoc w R - Interim	2	Lys309fs	ethA_p.Lys309fs
4327384	CT	C	INDEL	CDS	Rv3854c	reverse	ethA	K30fs	ETH-R	WHO2023	14.12.2023 VD	ethA_K30fs	2) Assoc w R - Interim	2	Lys30fs	ethA_p.Lys30fs
4326364	C	GAACAAATCC	INDEL	CDS	Rv3854c	reverse	ethA	K370fs	ETH-R	WHO2023	14.12.2023 VD	ethA_K370fs	2) Assoc w R - Interim	2	Lys370fs	ethA_p.Lys370fs
4327361	CGCTTT	C	INDEL	CDS	Rv3854c	reverse	ethA	K37fs	ETH-R	WHO2023	14.12.2023 VD	ethA_K37fs	1) Assoc w R	1	Lys37fs	ethA_p.Lys37fs
4326292	CT	GG	SNP	CDS	Rv3854c	reverse	ethA	K394_	ETH-R	WHO2023	14.12.2023 VD	ethA_K394_	2) Assoc w R - Interim	2	Lys394*	ethA_p.Lys394*
4326130	CT	C	INDEL	CDS	Rv3854c	reverse	ethA	K448fs	ETH-R	WHO2023	14.12.2023 VD	ethA_K448fs	2) Assoc w R - Interim	2	Lys448fs	ethA_p.Lys448fs
4326030	T	TG	INDEL	CDS	Rv3854c	reverse	ethA	K482fs	ETH-R	WHO2023	14.12.2023 VD	ethA_K482fs	2) Assoc w R - Interim	2	Lys482fs	ethA_p.Lys482fs
4327216	CTT	TTA	SNP	CDS	Rv3854c	reverse	ethA	K86_	ETH-R	WHO2023	14.12.2023 VD	ethA_K86_	2) Assoc w R - Interim	2	Lys86*	ethA_p.Lys86*
4326610	GCGCTGCAGC	G	INDEL	CDS	Rv3854c	reverse	ethA	L126fs	ETH-R	WHO2023	14.12.2023 VD	ethA_L126fs	2) Assoc w R - Interim	2	Leu126fs	ethA_p.Leu126fs
4326602	GGTGGGTGAG	T	INDEL	CDS	Rv3854c	reverse	ethA	L129fs	ETH-R	WHO2023	14.12.2023 VD	ethA_L129fs	2) Assoc w R - Interim	2	Leu129fs	ethA_p.Leu129fs
4326586	CAGCACGTTCT	C	INDEL	CDS	Rv3854c	reverse	ethA	L190fs	ETH-R	WHO2023	14.12.2023 VD	ethA_L190fs	2) Assoc w R - Interim	2	Leu190fs	ethA_p.Leu190fs
4326478	AACATCTTCCG	T	INDEL	CDS	Rv3854c	reverse	ethA	L194fs	ETH-R	WHO2023	14.12.2023 VD	ethA_L194fs	2) Assoc w R - Interim	2	Leu194fs	ethA_p.Leu194fs
4326799	GA	G	INDEL	CDS	Rv3854c	reverse	ethA	L225fs	ETH-R	WHO2023	14.12.2023 VD	ethA_L225fs	2) Assoc w R - Interim	2	Leu225fs	ethA_p.Leu225fs
4326676	GCTCAG	CCAAGCCCAT	INDEL	CDS	Rv3854c	reverse	ethA	L265fs	ETH-R	WHO2023	14.12.2023 VD	ethA_L265fs	2) Assoc w R - Interim	2	Leu265fs	ethA_p.Leu265fs
4326595	CA	TT	SNP	CDS	Rv3854c	reverse	ethA	L293_	ETH-R	WHO2023	14.12.2023 VD	ethA_L293_	2) Assoc w R - Interim	2	Leu293*	ethA_p.Leu293*
4325987	GTCGGCCTTCA	C	INDEL	CDS	Rv3854c	reverse	ethA	L295fs	ETH-R	WHO2023	14.12.2023 VD	ethA_L295fs	2) Assoc w R - Interim	2	Leu295fs	ethA_p.Leu295fs
4326053	ACTCCGACAC	T	INDEL	CDS	Rv3854c	reverse	ethA	L327fs	ETH-R	WHO2023	14.12.2023 VD	ethA_L327fs	2) Assoc w R - Interim	2	Leu327fs	ethA_p.Leu327fs
4326440	TTCAA	T	INDEL	CDS	Rv3854c	reverse	ethA	L344fs	ETH-R	WHO2023	14.12.2023 VD	ethA_L344fs	2) Assoc w R - Interim	2	Leu344fs	ethA_p.Leu344fs
4327369	CA	TC	SNP	CDS	Rv3854c	reverse	ethA	L35R	ETH-R	WHO2023	14.12.2023 VD	ethA_L35R	2) Assoc w R - Interim	2	Leu35Arg	ethA_p.Leu35Arg
4326298	C	CGT	INDEL	CDS	Rv3854c	reverse	ethA	L393fs	ETH-R	WHO2023	14.12.2023 VD	ethA_L393fs	2) Assoc w R - Interim	2	Leu393fs	ethA_p.Leu393fs
4326250	GTAATTCAACA	G	INDEL	CDS	Rv3854c	reverse	ethA	L405fs	ETH-R	WHO2023	14.12.2023 VD	ethA_L405fs	2) Assoc w R - Interim	2	Leu405fs	ethA_p.Leu405fs
4326247	CATGTAATTCA	C	INDEL	CDS	Rv3854c	reverse	ethA	L406fs	ETH-R	WHO2023	14.12.2023 VD	ethA_L406fs	2) Assoc w R - Interim	2	Leu406fs	ethA_p.Leu406fs
4326154	CA	C	INDEL	CDS	Rv3854c	reverse	ethA	L440fs	ETH-R	WHO2023	14.12.2023 VD	ethA_L440fs	2) Assoc w R - Interim	2	Leu440fs	ethA_p.Leu440fs
4326041	A	AG	INDEL	CDS	Rv3854c	reverse	ethA	L478fs	ETH-R	WHO2023	14.12.2023 VD	ethA_L478fs	2) Assoc w R - Interim	2	Leu478fs	ethA_p.Leu478fs
4327332	AC	A	INDEL	CDS	Rv3854c	reverse	ethA	L47fs	ETH-R	WHO2023	14.12.2023 VD	ethA_L47fs	2) Assoc w R - Interim	2	Leu47fs	ethA_p.Leu47fs
4327209	CTCTTGACGTA	C	INDEL	CDS	Rv3854c	reverse	ethA	L82fs	ETH-R	WHO2023	14.12.2023 VD	ethA_L82fs	2) Assoc w R - Interim	2	Leu82fs	ethA_p.Leu82fs
4325970	GACAGACAAA	T	INDEL	CDS	Rv3854c	reverse	ethA	LoF	ETH-R	WHO2023	14.12.2023 VD	ethA_LoF	1) Assoc w R	1	LoF	ethA_p.LoF
4326344	CTGCAGCATCG	A	INDEL	CDS	Rv3854c	reverse	ethA	M1?	ETH-R	WHO2023	14.12.2023 VD	ethA_M1?	1) Assoc w R	1	Met1?	ethA_p.Met1?
4326775	CA	C	INDEL	CDS	Rv3854c	reverse	ethA	M233fs	ETH-R	WHO2023	14.12.2023 VD	ethA_M233fs	2) Assoc w R - Interim	2	Met233fs	ethA_p.Met233fs
4326352	TCAATGGTGTC	A	INDEL	CDS	Rv3854c	reverse	ethA	M260fs	ETH-R	WHO2023	14.12.2023 VD	ethA_M260fs	2) Assoc w R - Interim	2	Met260fs	ethA_p.Met260fs
4326361	G	GC	INDEL	CDS	Rv3854c	reverse	ethA	M372fs	ETH-R	WHO2023	14.12.2023 VD	ethA_M372fs	2) Assoc w R - Interim	2	Met372fs	ethA_p.Met372fs
4326179	AT	A	INDEL	CDS	Rv3854c	reverse	ethA	M432fs	ETH-R	WHO2023	14.12.2023 VD	ethA_M432fs	2) Assoc w R - Interim	2	Met432fs	ethA_p.Met432fs
4327277	AAACCTAGCGT	A	INDEL	CDS	Rv3854c	reverse	ethA	M59fs	ETH-R	WHO2023	14.12.2023 VD	ethA_M59fs	2) Assoc w R - Interim	2	Met59fs	ethA_p.Met59fs
4327132	GT	G	INDEL	CDS	Rv3854c	reverse	ethA	N114fs	ETH-R	WHO2023	14.12.2023 VD	ethA_N114fs	2) Assoc w R - Interim	2	Asn114fs	ethA_p.Asn114fs
4326798	T	TG	INDEL	CDS	Rv3854c	reverse	ethA	N226fs	ETH-R	WHO2023	14.12.2023 VD	ethA_N226fs	2) Assoc w R - Interim	2	Asn226fs	ethA_p.Asn226fs
4326245	GCCGGATTCC	T	INDEL	CDS	Rv3854c	reverse	ethA	N242fs	ETH-R	WHO2023	14.12.2023 VD	ethA_N242fs	2) Assoc w R - Interim	2	Asn242fs	ethA_p.Asn242fs
4326613	GT	G	INDEL	CDS	Rv3854c	reverse	ethA	N287fs	ETH-R	WHO2023	14.12.2023 VD	ethA_N287fs	2) Assoc w R - Interim	2	Asn287fs	ethA_p.Asn287fs
4326080	TAGGCCATGT	A	INDEL	CDS	Rv3854c	reverse	ethA	N298fs	ETH-R	WHO2023	14.12.2023 VD	ethA_N298fs	2) Assoc w R - Interim	2	Asn298fs	ethA_p.Asn298fs
4326439	GT	G	INDEL	CDS	Rv3854c	reverse	ethA	N345fs	ETH-R	WHO2023	14.12.2023 VD	ethA_N345fs	2) Assoc w R - Interim	2	Asn345fs	ethA_p.Asn345fs
4326337	GT	G	INDEL	CDS	Rv3854c	reverse	ethA	N379fs	ETH-R	WHO2023	14.12.2023 VD	ethA_N379fs	2) Assoc w R - Interim	2	Asn379fs	ethA_p.Asn379fs
4326309	CA	C	INDEL	CDS	Rv3854c	reverse	ethA	N388fs	ETH-R	WHO2023	14.12.2023 VD	ethA_N388fs	2) Assoc w R - Interim	2	Asn388fs	ethA_p.Asn388fs
4326091	TAGTTCTGATT	G	INDEL	CDS	Rv3854c	reverse	ethA	N458fs	ETH-R	WHO2023	14.12.2023 VD	ethA_N458fs	2) Assoc w R - Interim	2	Asn458fs	ethA_p.Asn458fs
4326489	GCAGCACGTT	A	INDEL	CDS	Rv3854c	reverse	ethA	P160fs	ETH-R	WHO2023	14.12.2023 VD	ethA_P160fs	2) Assoc w R - Interim	2	Pro160fs	ethA_p.Pro160fs
4326539	CCGCATGCGC	A	INDEL	CDS	Rv3854c	reverse	ethA	P209fs	ETH-R	WHO2023	14.12.2023 VD	ethA_P209fs	2) Assoc w R - Interim	2	Pro209fs	ethA_p.Pro209fs
4326786	G	GAAGCC	INDEL	CDS	Rv3854c	reverse	ethA	P230fs	ETH-R	WHO2023	14.12.2023 VD	ethA_P230fs	2) Assoc w R - Interim	2	Pro230fs	ethA_p.Pro230fs
4326703	TG	T	INDEL	CDS	Rv3854c	reverse	ethA	P257fs	ETH-R	WHO2023	14.12.2023 VD	ethA_P257fs	2) Assoc w R - Interim	2	Pro257fs	ethA_p.Pro257fs
4326622	CG	C	INDEL	CDS	Rv3854c	reverse	ethA	P284fs	ETH-R	WHO2023	14.12.2023 VD	ethA_P284fs	2) Assoc w R - Interim	2	Pro284fs	ethA_p.Pro284fs
4327389	TCG	T	INDEL	CDS	Rv3854c	reverse	ethA	P28fs	ETH-R	WHO2023	14.12.2023 VD	ethA_P28fs	2) Assoc w R - Interim	2	Pro28fs	ethA_p.Pro28fs
4326208	CG	C	INDEL	CDS	Rv3854c	reverse	ethA	P422fs	ETH-R	WHO2023	14.12.2023 VD	ethA_P422fs	2) Assoc w R - Interim	2	Pro422fs	ethA_p.Pro422fs
4326166	TG	T	INDEL	CDS	Rv3854c	reverse	ethA	P436fs	ETH-R	WHO2023	14.12.2023 VD	ethA_P436fs	2) Assoc w R - Interim	2	Pro436fs	ethA_p.Pro436fs
4326954	CTTTGGATGTG	C	INDEL	CDS	Rv3854c	reverse	ethA	P68fs	ETH-R	WHO2023	14.12.2023 VD	ethA_P68fs	2) Assoc w R - Interim	2	Pro68fs	ethA_p.Pro68fs
4327106	TGGCTTTG	T	INDEL	CDS	Rv3854c	reverse	ethA	Q121_	ETH-R	WHO2023	14.12.2023 VD	ethA_Q121_	2) Assoc w R - Interim	2	Gln121*	ethA_p.Gln121*
4327106	TGGCTTTG	T	INDEL	CDS	Rv3854c	reverse	ethA	Q121fs	ETH-R	WHO2023	14.12.2023 VD	ethA_Q121fs	2) Assoc w R - Interim	2	Gln121fs	ethA_p.Gln121fs
4326979	CT	C	INDEL	CDS	Rv3854c	reverse	ethA	Q165fs	ETH-R	WHO2023	14.12.2023 VD	ethA_Q165fs	2) Assoc w R - Interim	2	Gln165fs	ethA_p.Gln165fs
4326856	CT	GG	SNP	CDS	Rv3854c	reverse	ethA	Q206_	ETH-R	WHO2023	14.12.2023 VD	ethA_Q206_	2) Assoc w R - Interim	2	Gln206*	ethA_p.Gln206*
4326829	CTG	TCA	SNP	CDS	Rv3854c	reverse	ethA	Q215_	ETH-R	WHO2023	14.12.2023 VD	ethA_Q215_	2) Assoc w R - Interim	2	Gln215*	ethA_p.Gln215*
4326330	TCGGTCACCAC	A	INDEL	CDS	Rv3854c	reverse	ethA	Q24_	ETH-R	WHO2023	14.12.2023 VD	ethA_Q24_	2) Assoc w R - Interim	2	Gln24*	ethA_p.Gln24*
4326330	TCGGTCACCAC	A	INDEL	CDS	Rv3854c	reverse	ethA	Q246_	ETH-R	WHO2023	14.12.2023 VD	ethA_Q246_	2) Assoc w R - Interim	2	Gln246*	ethA_p.Gln246*
4326330	TCGGTCACCAC	A	INDEL	CDS	Rv3854c	reverse	ethA	Q246fs	ETH-R	WHO2023	14.12.2023 VD	ethA_Q246fs	2) Assoc w R - Interim	2	Gln246fs	ethA_p.Gln246fs
4326712	CT	TG	SNP	CDS	Rv3854c	reverse	ethA	Q254_	ETH-R	WHO2023	14.12.2023 VD	ethA_Q254_	2) Assoc w R - Interim	2	Gln254*	ethA_p.Gln254*
4326217	TTCAACCCCGT	G	INDEL	CDS	Rv3854c	reverse	ethA	Q269_	ETH-R	WHO2023	14.12.2023 VD	ethA_Q269_	2) Assoc w R - Interim	2	Gln269*	ethA_p.Gln269*
4326217	TTCAACCCCGT	G	INDEL	CDS	Rv3854c	reverse	ethA	Q269fs	ETH-R	WHO2023	14.12.2023 VD	ethA_Q269fs	2) Assoc w R - Interim	2	Gln269fs	ethA_p.Gln269fs
4326215	AGGTTCAACC	T	INDEL	CDS	Rv3854c	reverse	ethA	Q271fs	ETH-R	WHO2023	14.12.2023 VD	ethA_Q271fs	2) Assoc w R - Interim	2	Gln271fs	ethA_p.Gln271fs

4326601	CT	GG	SNP	CDS	Rv3854c	reverse	ethA	Q291_	ETH-R	WHO2023	14.12.2023 VD	ethA_Q291_	2) Assoc w R - Interim	2	Gln291*	ethA_p.Gln291*
4326433	CTG	TCA	SNP	CDS	Rv3854c	reverse	ethA	Q347_	ETH-R	WHO2023	14.12.2023 VD	ethA_Q347_	2) Assoc w R - Interim	2	Gln347*	ethA_p.Gln347*
4326397	TTG	CTA	SNP	CDS	Rv3854c	reverse	ethA	Q359_	ETH-R	WHO2023	14.12.2023 VD	ethA_Q359_	2) Assoc w R - Interim	2	Gln359*	ethA_p.Gln359*
4326394	TT	AC	SNP	CDS	Rv3854c	reverse	ethA	Q360_	ETH-R	WHO2023	14.12.2023 VD	ethA_Q360_	2) Assoc w R - Interim	2	Gln360*	ethA_p.Gln360*
4326127	CT	GG	SNP	CDS	Rv3854c	reverse	ethA	Q449_	ETH-R	WHO2023	14.12.2023 VD	ethA_Q449_	2) Assoc w R - Interim	2	Gln449*	ethA_p.Gln449*
4327254	CCT	C	INDEL	CDS	Rv3854c	reverse	ethA	Q73_	ETH-R	WHO2023	14.12.2023 VD	ethA_Q73_	2) Assoc w R - Interim	2	Gln73*	ethA_p.Gln73*
4327254	CCT	C	INDEL	CDS	Rv3854c	reverse	ethA	Q73fs	ETH-R	WHO2023	14.12.2023 VD	ethA_Q73fs	2) Assoc w R - Interim	2	Gln73fs	ethA_p.Gln73fs
4326852	AGC	A	INDEL	CDS	Rv3854c	reverse	ethA	R207fs	ETH-R	WHO2023	14.12.2023 VD	ethA_R207fs	2) Assoc w R - Interim	2	Arg207fs	ethA_p.Arg207fs
4326702	G	GT	INDEL	CDS	Rv3854c	reverse	ethA	R258fs	ETH-R	WHO2023	14.12.2023 VD	ethA_R258fs	2) Assoc w R - Interim	2	Arg258fs	ethA_p.Arg258fs
4326698	C	CGT	INDEL	CDS	Rv3854c	reverse	ethA	R259fs	ETH-R	WHO2023	14.12.2023 VD	ethA_R259fs	2) Assoc w R - Interim	2	Arg259fs	ethA_p.Arg259fs
4326637	TCG	CTA	SNP	CDS	Rv3854c	reverse	ethA	R279_	ETH-R	WHO2023	14.12.2023 VD	ethA_R279_	2) Assoc w R - Interim	2	Arg279*	ethA_p.Arg279*
4326598	TC	CT	SNP	CDS	Rv3854c	reverse	ethA	R292_	ETH-R	WHO2023	14.12.2023 VD	ethA_R292_	2) Assoc w R - Interim	2	Arg292*	ethA_p.Arg292*
4326211	TCG	CTA	SNP	CDS	Rv3854c	reverse	ethA	R421_	ETH-R	WHO2023	14.12.2023 VD	ethA_R421_	2) Assoc w R - Interim	2	Arg421*	ethA_p.Arg421*
4326212	C	CGCTCG	INDEL	CDS	Rv3854c	reverse	ethA	R421fs	ETH-R	WHO2023	14.12.2023 VD	ethA_R421fs	2) Assoc w R - Interim	2	Arg421fs	ethA_p.Arg421fs
4326118	AC	A	INDEL	CDS	Rv3854c	reverse	ethA	R452fs	ETH-R	WHO2023	14.12.2023 VD	ethA_R452fs	2) Assoc w R - Interim	2	Arg452fs	ethA_p.Arg452fs
4326086	CG	C	INDEL	CDS	Rv3854c	reverse	ethA	R463fs	ETH-R	WHO2023	14.12.2023 VD	ethA_R463fs	2) Assoc w R - Interim	2	Arg463fs	ethA_p.Arg463fs
4326018	ACCCTCGTCG	G	INDEL	CDS	Rv3854c	reverse	ethA	R466fs	ETH-R	WHO2023	14.12.2023 VD	ethA_R466fs	2) Assoc w R - Interim	2	Arg466fs	ethA_p.Arg466fs
4326066	GC	G	INDEL	CDS	Rv3854c	reverse	ethA	R470fs	ETH-R	WHO2023	14.12.2023 VD	ethA_R470fs	2) Assoc w R - Interim	2	Arg470fs	ethA_p.Arg470fs
4326038	C	CG	INDEL	CDS	Rv3854c	reverse	ethA	R479fs	ETH-R	WHO2023	14.12.2023 VD	ethA_R479fs	2) Assoc w R - Interim	2	Arg479fs	ethA_p.Arg479fs
4326556	CACGACGATG	A	INDEL	CDS	Rv3854c	reverse	ethA	R54fs	ETH-R	WHO2023	14.12.2023 VD	ethA_R54fs	2) Assoc w R - Interim	2	Arg54fs	ethA_p.Arg54fs
4327279	TC	AG	SNP	CDS	Rv3854c	reverse	ethA	R65_	ETH-R	WHO2023	14.12.2023 VD	ethA_R65_	2) Assoc w R - Interim	2	Arg65*	ethA_p.Arg65*
4327158	T	TG	INDEL	CDS	Rv3854c	reverse	ethA	S106fs	ETH-R	WHO2023	14.12.2023 VD	ethA_S106fs	2) Assoc w R - Interim	2	Ser106fs	ethA_p.Ser106fs
4327106	TGG	T	INDEL	CDS	Rv3854c	reverse	ethA	S122fs	ETH-R	WHO2023	14.12.2023 VD	ethA_S122fs	2) Assoc w R - Interim	2	Ser122fs	ethA_p.Ser122fs
4327060	G	GAACTTGGC	INDEL	CDS	Rv3854c	reverse	ethA	S138fs	ETH-R	WHO2023	14.12.2023 VD	ethA_S138fs	2) Assoc w R - Interim	2	Ser138fs	ethA_p.Ser138fs
4326436	CCGCCTGGCG	C	INDEL	CDS	Rv3854c	reverse	ethA	S148fs	ETH-R	WHO2023	14.12.2023 VD	ethA_S148fs	2) Assoc w R - Interim	2	Ser148fs	ethA_p.Ser148fs
4326964	TCGATTCCAT	G	INDEL	CDS	Rv3854c	reverse	ethA	S18fs	ETH-R	WHO2023	14.12.2023 VD	ethA_S18fs	2) Assoc w R - Interim	2	Ser18fs	ethA_p.Ser18fs
4326883	CG	TT	SNP	CDS	Rv3854c	reverse	ethA	S197_	ETH-R	WHO2023	14.12.2023 VD	ethA_S197_	2) Assoc w R - Interim	2	Ser197*	ethA_p.Ser197*
4326850	TGA	AGG	SNP	CDS	Rv3854c	reverse	ethA	S208_	ETH-R	WHO2023	14.12.2023 VD	ethA_S208_	2) Assoc w R - Interim	2	Ser208*	ethA_p.Ser208*
4326850	TGA	T	INDEL	CDS	Rv3854c	reverse	ethA	S208fs	ETH-R	WHO2023	14.12.2023 VD	ethA_S208fs	2) Assoc w R - Interim	2	Ser208fs	ethA_p.Ser208fs
4326436	GTGCTTTCGC	C	INDEL	CDS	Rv3854c	reverse	ethA	S214fs	ETH-R	WHO2023	14.12.2023 VD	ethA_S214fs	2) Assoc w R - Interim	2	Ser214fs	ethA_p.Ser214fs
4326721	G	GCT	INDEL	CDS	Rv3854c	reverse	ethA	S251fs	ETH-R	WHO2023	14.12.2023 VD	ethA_S251fs	2) Assoc w R - Interim	2	Ser251fs	ethA_p.Ser251fs
4326367	TTCAATGGTG	G	INDEL	CDS	Rv3854c	reverse	ethA	S266fs	ETH-R	WHO2023	14.12.2023 VD	ethA_S266fs	2) Assoc w R - Interim	2	Ser266fs	ethA_p.Ser266fs
4326765	AGAGGAATTC	C	INDEL	CDS	Rv3854c	reverse	ethA	S31fs	ETH-R	WHO2023	14.12.2023 VD	ethA_S31fs	2) Assoc w R - Interim	2	Ser31fs	ethA_p.Ser31fs
4326342	GATGCCGGA	G	INDEL	CDS	Rv3854c	reverse	ethA	S375fs	ETH-R	WHO2023	14.12.2023 VD	ethA_S375fs	2) Assoc w R - Interim	2	Ser375fs	ethA_p.Ser375fs
4326305	GA	G	INDEL	CDS	Rv3854c	reverse	ethA	S390fs	ETH-R	WHO2023	14.12.2023 VD	ethA_S390fs	2) Assoc w R - Interim	2	Ser390fs	ethA_p.Ser390fs
4326202	TGA	T	INDEL	CDS	Rv3854c	reverse	ethA	S424fs	ETH-R	WHO2023	14.12.2023 VD	ethA_S424fs	2) Assoc w R - Interim	2	Ser424fs	ethA_p.Ser424fs
4326144	CCAGCGAG	C	INDEL	CDS	Rv3854c	reverse	ethA	S442fs	ETH-R	WHO2023	14.12.2023 VD	ethA_S442fs	2) Assoc w R - Interim	2	Ser442fs	ethA_p.Ser442fs
4326113	GTGTACGCG	G	INDEL	CDS	Rv3854c	reverse	ethA	S451fs	ETH-R	WHO2023	14.12.2023 VD	ethA_S451fs	2) Assoc w R - Interim	2	Ser451fs	ethA_p.Ser451fs
4326712	CGAGCGTGAC	C	INDEL	CDS	Rv3854c	reverse	ethA	T125fs	ETH-R	WHO2023	14.12.2023 VD	ethA_T125fs	2) Assoc w R - Interim	2	Thr125fs	ethA_p.Thr125fs
4327083	AGG	A	INDEL	CDS	Rv3854c	reverse	ethA	T130fs	ETH-R	WHO2023	14.12.2023 VD	ethA_T130fs	2) Assoc w R - Interim	2	Thr130fs	ethA_p.Thr130fs
4326919	T	TGC	INDEL	CDS	Rv3854c	reverse	ethA	T186fs	ETH-R	WHO2023	14.12.2023 VD	ethA_T186fs	2) Assoc w R - Interim	2	Thr186fs	ethA_p.Thr186fs
4326844	GGT	G	INDEL	CDS	Rv3854c	reverse	ethA	T210fs	ETH-R	WHO2023	14.12.2023 VD	ethA_T210fs	2) Assoc w R - Interim	2	Thr210fs	ethA_p.Thr210fs
4326775	CATGGT	C	INDEL	CDS	Rv3854c	reverse	ethA	T232fs	ETH-R	WHO2023	14.12.2023 VD	ethA_T232fs	2) Assoc w R - Interim	2	Thr232fs	ethA_p.Thr232fs
4326400	TGGGCACCAA	T	INDEL	CDS	Rv3854c	reverse	ethA	T236fs	ETH-R	WHO2023	14.12.2023 VD	ethA_T236fs	2) Assoc w R - Interim	2	Thr236fs	ethA_p.Thr236fs
4326533	GT	G	INDEL	CDS	Rv3854c	reverse	ethA	T314fs	ETH-R	WHO2023	14.12.2023 VD	ethA_T314fs	2) Assoc w R - Interim	2	Thr314fs	ethA_p.Thr314fs
4326334	AAAAAGCTGC	C	INDEL	CDS	Rv3854c	reverse	ethA	T316fs	ETH-R	WHO2023	14.12.2023 VD	ethA_T316fs	2) Assoc w R - Interim	2	Thr316fs	ethA_p.Thr316fs
4325972	TCTTCGACAT	T	INDEL	CDS	Rv3854c	reverse	ethA	T353fs	ETH-R	WHO2023	14.12.2023 VD	ethA_T353fs	2) Assoc w R - Interim	2	Thr353fs	ethA_p.Thr353fs
4326235	GCCAACCGTG	A	INDEL	CDS	Rv3854c	reverse	ethA	T355fs	ETH-R	WHO2023	14.12.2023 VD	ethA_T355fs	2) Assoc w R - Interim	2	Thr355fs	ethA_p.Thr355fs
4326384	T	TCA	INDEL	CDS	Rv3854c	reverse	ethA	T364fs	ETH-R	WHO2023	14.12.2023 VD	ethA_T364fs	2) Assoc w R - Interim	2	Thr364fs	ethA_p.Thr364fs
4326314	G	GT	INDEL	CDS	Rv3854c	reverse	ethA	T387fs	ETH-R	WHO2023	14.12.2023 VD	ethA_T387fs	2) Assoc w R - Interim	2	Thr387fs	ethA_p.Thr387fs
4326226	GGT	G	INDEL	CDS	Rv3854c	reverse	ethA	T416fs	ETH-R	WHO2023	14.12.2023 VD	ethA_T416fs	2) Assoc w R - Interim	2	Thr416fs	ethA_p.Thr416fs
4327342	GGT	G	INDEL	CDS	Rv3854c	reverse	ethA	T44fs	ETH-R	WHO2023	14.12.2023 VD	ethA_T44fs	2) Assoc w R - Interim	2	Thr44fs	ethA_p.Thr44fs
4327292	GT	G	INDEL	CDS	Rv3854c	reverse	ethA	T61fs	ETH-R	WHO2023	14.12.2023 VD	ethA_T61fs	2) Assoc w R - Interim	2	Thr61fs	ethA_p.Thr61fs
4327211	GT	G	INDEL	CDS	Rv3854c	reverse	ethA	T88fs	ETH-R	WHO2023	14.12.2023 VD	ethA_T88fs	2) Assoc w R - Interim	2	Thr88fs	ethA_p.Thr88fs
4326636	CGGCACGAG	T	INDEL	CDS	Rv3854c	reverse	ethA	V104fs	ETH-R	WHO2023	14.12.2023 VD	ethA_V104fs	2) Assoc w R - Interim	2	Val104fs	ethA_p.Val104fs
4327107	CTTTGGATGT	G	INDEL	CDS	Rv3854c	reverse	ethA	V118fs	ETH-R	WHO2023	14.12.2023 VD	ethA_V118fs	2) Assoc w R - Interim	2	Val118fs	ethA_p.Val118fs
4326999	CGA	C	INDEL	CDS	Rv3854c	reverse	ethA	V158fs	ETH-R	WHO2023	14.12.2023 VD	ethA_V158fs	2) Assoc w R - Interim	2	Val158fs	ethA_p.Val158fs
4326436	ACATCTTCCGC	C	INDEL	CDS	Rv3854c	reverse	ethA	V179fs	ETH-R	WHO2023	14.12.2023 VD	ethA_V179fs	2) Assoc w R - Interim	2	Val179fs	ethA_p.Val179fs
4326901	CA	C	INDEL	CDS	Rv3854c	reverse	ethA	V191fs	ETH-R	WHO2023	14.12.2023 VD	ethA_V191fs	2) Assoc w R - Interim	2	Val191fs	ethA_p.Val191fs
4326868	GA	AC	SNP	CDS	Rv3854c	reverse	ethA	V202G	ETH-R	WHO2023	14.12.2023 VD	ethA_V202G	2) Assoc w R - Interim	2	Val202Gly	ethA_p.Val202Gly
4326437	AATCGCTGGTC	A	INDEL	CDS	Rv3854c	reverse	ethA	V238fs	ETH-R	WHO2023	14.12.2023 VD	ethA_V238fs	2) Assoc w R - Interim	2	Val238fs	ethA_p.Val238fs
4326276	ATTCCGGTCGC	C	INDEL	CDS	Rv3854c	reverse	ethA	V249fs	ETH-R	WHO2023	14.12.2023 VD	ethA_V249fs	2) Assoc w R - Interim	2	Val249fs	ethA_p.Val249fs
4326587	AC	A	INDEL	CDS	Rv3854c	reverse	ethA	V296fs	ETH-R	WHO2023	14.12.2023 VD	ethA_V296fs	2) Assoc w R - Interim	2	Val296fs	ethA_p.Val296fs
4326536	AC	A	INDEL	CDS	Rv3854c	reverse	ethA	V313fs	ETH-R	WHO2023	14.12.2023 VD	ethA_V313fs	2) Assoc w R - Interim	2	Val313fs	ethA_p.Val313fs
4326324	C	GTGTAGGCCA	INDEL	CDS	Rv3854c	reverse	ethA	V384fs	ETH-R	WHO2023	14.12.2023 VD	ethA_V384fs	2) Assoc w R - Interim	2	Val384fs	ethA_p.Val384fs
4326003	GCTAAA	G	INDEL	CDS	Rv3854c	reverse	ethA	V489fs	ETH-R	WHO2023	14.12.2023 VD	ethA_V489fs	2) Assoc w R - Interim	2	Val489fs	ethA_p.Val489fs
4327220	A	AC	INDEL	CDS	Rv3854c	reverse	ethA	V85fs	ETH-R	WHO2023	14.12.2023 VD	ethA_V85fs	2) Assoc w R - Interim	2	Val85fs	ethA_p.Val85fs
4327062	GCGCTGAGC	T	INDEL	CDS	Rv3854c	reverse	ethA	W116_	ETH-R	WHO2023	14.12.2023 VD	ethA_W116_	2) Assoc w R - Interim	2	Trp116*	ethA_p.Trp116*
4326972	GC	G	INDEL	CDS	Rv3854c	reverse	ethA	W167_	ETH-R	WHO2023	14.12.2023 VD	ethA_W167_	2) Assoc w R - Interim	2	Trp167*	ethA_p.Trp167*
4326972	GC	G	INDEL	CDS	Rv3854c	reverse	ethA	W167fs	ETH-R	WHO2023	14.12.2023 VD	ethA_W167fs	2) Assoc w R - Interim	2	Trp167fs	ethA_p.Trp167fs
4326940	GGAACCGGAT	G	INDEL	CDS	Rv3854c	reverse	ethA	W21_	ETH-R	WHO2023	14.12.2023 VD	ethA_W21_	2) Assoc w R - Interim	2	Trp21*	ethA_p.Trp21*
4326940	GGAACCGGAT	G	INDEL	CDS	Rv3854c	reverse	ethA	W21fs	ETH-R	WHO2023	14.12.2023 VD	ethA_W21fs	2) Assoc w R - Interim	2	Trp21fs	ethA_p.Trp21fs
4326789	GC	G	INDEL	CDS	Rv3854c	reverse	ethA	W228_	ETH-R	WHO2023	14.12.2023 VD	ethA_W228_	2) Assoc w R - Interim	2	Trp228*	ethA_p.Trp228*
4326789	GC	G	INDEL	CDS	Rv3854c	reverse	ethA	W228fs	ETH-R	WHO2023	14.12.2023 VD	ethA_W228fs	2) Assoc w R - Interim	2	Trp228fs	ethA_p.Trp228fs
4326754	CC	TT	SNP	CDS	Rv3854c	reverse	ethA	W240_	ETH-R	WHO2023	14.12.2023 VD	ethA_W240_	2) Assoc w R - Interim	2	Trp240*	ethA_p.Trp240*
4326705	GCC	G	INDEL	CDS	Rv3854c	reverse	ethA	W256_	ETH-R	WHO2023	14.12.2023 VD	ethA_W256_	2) Assoc w R - Interim	2	Trp256*	ethA_p.Trp256*
4326705	GCC	G	INDEL	CDS	Rv3854c	reverse	ethA	W256fs	ETH-R	WHO2023	14.12.2023 VD	ethA_W256fs	2) Assoc w R - Interim	2	Trp256fs	ethA_p.Trp256fs
4326539	AACAGGTCGC	A	INDEL	CDS	Rv3854c	reverse	ethA	W289fs	ETH-R	WHO2023	14.12.2023 VD	ethA_W289fs	2) Assoc w R - Interim	2	Trp289fs	ethA_p.Trp289fs
4326109	C	T	SNP	CDS	Rv3854c	reverse	ethA	W45_	ETH-R	WHO2023	14.12.2023 VD	ethA_W45_	2) Assoc w R - Interim	2	Trp45*	ethA_p.Trp45*
4326109	C	T	SNP	CDS	Rv3854c	reverse	ethA	W455_	ETH-R	WHO2023	14.12.2023 VD	ethA_W455_	2) Assoc w R - Interim	2	Trp455*	ethA_p.Trp455*
4326959	ACCAATCGGC	T	INDEL	CDS	Rv3854c	reverse	ethA	W45fs	ETH-R	WHO2023	14.12.2023 VD	ethA_W45fs	2) Assoc w R - Interim	2	Trp45fs	ethA_p.Trp45fs
4327260	GTCCGGTCC	G	INDEL	CDS	Rv3854c	reverse	ethA	W69fs	ETH-R	WHO2023	14.12.2023 VD	ethA_W69fs	2) Assoc w R - Interim	2	Trp69fs	ethA_p.Trp69fs
4327054	G	C	SNP	CDS	Rv3854c	reverse	ethA	Y140_	ETH-R	WHO2023	14.12.2023 VD	ethA_Y140_	2) Assoc w R - Interim	2	Tyr140*	ethA_p.Tyr140*
4327045	GT	TC	SNP	CDS	Rv3854c	reverse	ethA	Y143_	ETH-R	WHO2023	14.12.2023 VD	ethA_Y143_	2) Assoc w R - Interim	2	Tyr143*	ethA_p.Tyr143*
4326635	CGACACGATG	T	INDEL	CDS	Rv3854c	reverse	ethA	Y147fs	ETH-R	WHO2023	14.12.2023 VD	ethA_Y147fs	2) Assoc w R - Interim	2	Tyr147fs	ethA_p.Tyr147fs

4326215	GTGTCGGTCAC	T	INDEL	CDS	Rv3854c	reverse	ethA	Y211fs	ETH-R	WHO2023	14.12.2023 VD	ethA_Y211fs	2) Assoc w R - Interim	2	Tyr211fs	ethA_p.Tyr211fs
4326768	TG	T	INDEL	CDS	Rv3854c	reverse	ethA	Y235fs	ETH-R	WHO2023	14.12.2023 VD	ethA_Y235fs	1) Assoc w R	1	Tyr235fs	ethA_p.Tyr235fs
4326724	GT	TC	SNP	CDS	Rv3854c	reverse	ethA	Y250_	ETH-R	WHO2023	14.12.2023 VD	ethA_Y250_	2) Assoc w R - Interim	2	Tyr250*	ethA_p.Tyr250*
4326293	TCAGCCGGCA	T	INDEL	CDS	Rv3854c	reverse	ethA	Y276fs	ETH-R	WHO2023	14.12.2023 VD	ethA_Y276fs	2) Assoc w R - Interim	2	Tyr276fs	ethA_p.Tyr276fs
4327360	TTTCCAGGATC	C	INDEL	CDS	Rv3854c	reverse	ethA	Y32fs	ETH-R	WHO2023	14.12.2023 VD	ethA_Y32fs	2) Assoc w R - Interim	2	Tyr32fs	ethA_p.Tyr32fs
4326367	GT	G	INDEL	CDS	Rv3854c	reverse	ethA	Y369fs	ETH-R	WHO2023	14.12.2023 VD	ethA_Y369fs	2) Assoc w R - Interim	2	Tyr369fs	ethA_p.Tyr369fs
4326328	G	C	SNP	CDS	Rv3854c	reverse	ethA	Y382_	ETH-R	WHO2023	14.12.2023 VD	ethA_Y382_	2) Assoc w R - Interim	2	Tyr382*	ethA_p.Tyr382*
4326317	TA	T	INDEL	CDS	Rv3854c	reverse	ethA	Y386fs	ETH-R	WHO2023	14.12.2023 VD	ethA_Y386fs	2) Assoc w R - Interim	2	Tyr386fs	ethA_p.Tyr386fs
4326250	GT	TC	SNP	CDS	Rv3854c	reverse	ethA	Y408_	ETH-R	WHO2023	14.12.2023 VD	ethA_Y408_	2) Assoc w R - Interim	2	Tyr408*	ethA_p.Tyr408*
4326160	GT	TC	SNP	CDS	Rv3854c	reverse	ethA	Y438_	ETH-R	WHO2023	14.12.2023 VD	ethA_Y438_	2) Assoc w R - Interim	2	Tyr438*	ethA_p.Tyr438*
4327293	TG	T	INDEL	CDS	Rv3854c	reverse	ethA	Y60_	ETH-R	WHO2023	14.12.2023 VD	ethA_Y60_	2) Assoc w R - Interim	2	Tyr60*	ethA_p.Tyr60*
4327293	TG	T	INDEL	CDS	Rv3854c	reverse	ethA	Y60fs	ETH-R	WHO2023	14.12.2023 VD	ethA_Y60fs	2) Assoc w R - Interim	2	Tyr60fs	ethA_p.Tyr60fs
4327222	GT	TC	SNP	CDS	Rv3854c	reverse	ethA	Y84_	ETH-R	WHO2023	14.12.2023 VD	ethA_Y84_	2) Assoc w R - Interim	2	Tyr84*	ethA_p.Tyr84*
4327198	AT	TC	SNP	CDS	Rv3854c	reverse	ethA	Y92_	ETH-R	WHO2023	14.12.2023 VD	ethA_Y92_	2) Assoc w R - Interim	2	Tyr92*	ethA_p.Tyr92*
3640145	AGTGTGCCGG	C	INDEL	PROM	Rv3261	forward	fbiA	LoF	DLM-R	WHO2023	14.12.2023 VD	fbiA_LoF	2) Assoc w R - Interim	2	LoF	fbiA_c.LoF
3641521	G	GC	INDEL	PROM	Rv3261	forward	fbiA	V329fs	DLM-R	WHO2023	14.12.2023 VD	fbiA_V329fs	2) Assoc w R - Interim	2	Val329fs	fbiA_c.Val329fs
3641538	ACC	A	INDEL	CDS	Rv3262	forward	fbiB	LoF	DLM-R	WHO2023	14.12.2023 VD	fbiB_LoF	2) Assoc w R - Interim	2	LoF	fbiB_p.LoF
3641579	GC	G	INDEL	CDS	Rv3262	forward	fbiB	V17fs	DLM-R	WHO2023	14.12.2023 VD	fbiB_V17fs	2) Assoc w R - Interim	2	Val17fs	fbiB_p.Val17fs
1304386	CTCCGGAACG	C	INDEL	PROM	Rv1173	forward	fbiC	A487fs	DLM-R	WHO2023	14.12.2023 VD	fbiC_A487fs	2) Assoc w R - Interim	2	Ala487fs	fbiC_c.Ala487fs
1304841	G	GAT	INDEL	PROM	Rv1173	forward	fbiC	A639fs	DLM-R	WHO2023	14.12.2023 VD	fbiC_A639fs	2) Assoc w R - Interim	2	Ala639fs	fbiC_c.Ala639fs
1305494	CAATCTGGCCT	C	INDEL	PROM	Rv1173	forward	fbiC	A855fs	DLM-R	WHO2023	14.12.2023 VD	fbiC_A855fs	2) Assoc w R - Interim	2	Ala855fs	fbiC_c.Ala855fs
1305476	C	TACGCCCTGC	INDEL	PROM	Rv1173	forward	fbiC	A856fs	DLM-R	WHO2023	14.12.2023 VD	fbiC_A856fs	2) Assoc w R - Interim	2	Ala856fs	fbiC_c.Ala856fs
1302695	GGTCGGAAGT	C	INDEL	PROM	Rv1173	forward	fbiC	LoF	DLM-R	WHO2023	14.12.2023 VD	fbiC_LoF	2) Assoc w R - Interim	2	LoF	fbiC_c.LoF
1304011	A	AC	INDEL	PROM	Rv1173	forward	fbiC	P362fs	DLM-R	WHO2023	14.12.2023 VD	fbiC_P362fs	2) Assoc w R - Interim	2	Pro362fs	fbiC_c.Pro362fs
1304253	G	GGC	INDEL	PROM	Rv1173	forward	fbiC	S443fs	DLM-R	WHO2023	14.12.2023 VD	fbiC_S443fs	2) Assoc w R - Interim	2	Ser443fs	fbiC_c.Ser443fs
490709	ACCATTTTCAG	A	INDEL	PROM	Rv0407	forward	fgd1	LoF	DLM-R	WHO2023	14.12.2023 VD	fgd1_LoF	2) Assoc w R - Interim	2	LoF	fgd1_c.LoF
491114	ACGAAATCGCC	T	INDEL	PROM	Rv0407	forward	fgd1	N112fs	DLM-R	WHO2023	14.12.2023 VD	fgd1_N112fs	2) Assoc w R - Interim	2	Asn112fs	fgd1_c.Asn112fs
4407845	CGG	C	INDEL	CDS	Rv3919c	reverse	gid	A119fs	SM-R	WHO2023	14.12.2023 VD	gid_A119fs	2) Assoc w R - Interim	2	Ala119fs	gid_p.Ala119fs
4407804	AG	A	INDEL	CDS	Rv3919c	reverse	gid	A133fs	SM-R	WHO2023	14.12.2023 VD	gid_A133fs	2) Assoc w R - Interim	2	Ala133fs	gid_p.Ala133fs
4407792	CCGTGACACCG	C	INDEL	CDS	Rv3919c	reverse	gid	A134fs	SM-R	WHO2023	14.12.2023 VD	gid_A134fs	2) Assoc w R - Interim	2	Ala134fs	gid_p.Ala134fs
4407787	ACCGC	A	INDEL	CDS	Rv3919c	reverse	gid	A138fs	SM-R	WHO2023	14.12.2023 VD	gid_A138fs	2) Assoc w R - Interim	2	Ala138fs	gid_p.Ala138fs
4407784	G	GC	INDEL	CDS	Rv3919c	reverse	gid	A140fs	SM-R	WHO2023	14.12.2023 VD	gid_A140fs	2) Assoc w R - Interim	2	Ala140fs	gid_p.Ala140fs
4407702	AG	A	INDEL	CDS	Rv3919c	reverse	gid	A167fs	SM-R	WHO2023	14.12.2023 VD	gid_A167fs	2) Assoc w R - Interim	2	Ala167fs	gid_p.Ala167fs
4407657	G	GC	INDEL	CDS	Rv3919c	reverse	gid	A183fs	SM-R	WHO2023	14.12.2023 VD	gid_A183fs	2) Assoc w R - Interim	2	Ala183fs	gid_p.Ala183fs
4407623	TCG	T	INDEL	CDS	Rv3919c	reverse	gid	A193fs	SM-R	WHO2023	14.12.2023 VD	gid_A193fs	2) Assoc w R - Interim	2	Ala193fs	gid_p.Ala193fs
4408147	G	GCAAGCGAC	INDEL	CDS	Rv3919c	reverse	gid	A19fs	SM-R	WHO2023	14.12.2023 VD	gid_A19fs	2) Assoc w R - Interim	2	Ala19fs	gid_p.Ala19fs
4407603	CG	TT	SNP	CDS	Rv3919c	reverse	gid	A200E	SM-R	WHO2023	14.12.2023 VD	gid_A200E	1) Assoc w R	1	Ala200Glu	gid_p.Ala200Glu
4407603	CG	C	INDEL	CDS	Rv3919c	reverse	gid	A200fs	SM-R	WHO2023	14.12.2023 VD	gid_A200fs	2) Assoc w R - Interim	2	Ala200fs	gid_p.Ala200fs
4407589	GC	G	INDEL	CDS	Rv3919c	reverse	gid	A205fs	SM-R	WHO2023	14.12.2023 VD	gid_A205fs	2) Assoc w R - Interim	2	Ala205fs	gid_p.Ala205fs
4407957	CG	C	INDEL	CDS	Rv3919c	reverse	gid	A82fs	SM-R	WHO2023	14.12.2023 VD	gid_A82fs	2) Assoc w R - Interim	2	Ala82fs	gid_p.Ala82fs
4407628	CCA	C	INDEL	CDS	Rv3919c	reverse	gid	C191fs	SM-R	WHO2023	14.12.2023 VD	gid_C191fs	2) Assoc w R - Interim	2	Cys191fs	gid_p.Cys191fs
4408047	GCA	ACG	SNP	CDS	Rv3919c	reverse	gid	C52_	SM-R	WHO2023	14.12.2023 VD	gid_C52_	2) Assoc w R - Interim	2	Cys52*	gid_p.Cys52*
4407882	A	ATCTGC	INDEL	CDS	Rv3919c	reverse	gid	D107fs	SM-R	WHO2023	14.12.2023 VD	gid_D107fs	2) Assoc w R - Interim	2	Asp107fs	gid_p.Asp107fs
4407583	ACGCAAATAG	C	INDEL	CDS	Rv3919c	reverse	gid	D185fs	SM-R	WHO2023	14.12.2023 VD	gid_D185fs	2) Assoc w R - Interim	2	Asp185fs	gid_p.Asp185fs
4408065	GT	G	INDEL	CDS	Rv3919c	reverse	gid	D46fs	SM-R	WHO2023	14.12.2023 VD	gid_D46fs	2) Assoc w R - Interim	2	Asp46fs	gid_p.Asp46fs
4408015	TC	T	INDEL	CDS	Rv3919c	reverse	gid	D63fs	SM-R	WHO2023	14.12.2023 VD	gid_D63fs	2) Assoc w R - Interim	2	Asp63fs	gid_p.Asp63fs
4408001	TA	T	INDEL	CDS	Rv3919c	reverse	gid	D67fs	SM-R	WHO2023	14.12.2023 VD	gid_D67fs	2) Assoc w R - Interim	2	Asp67fs	gid_p.Asp67fs
4408002	ATC	GTG	SNP	CDS	Rv3919c	reverse	gid	D67H	SM-R	WHO2023	14.12.2023 VD	gid_D67H	2) Assoc w R - Interim	2	Asp67His	gid_p.Asp67His
4407894	CT	GG	SNP	CDS	Rv3919c	reverse	gid	E103_	SM-R	WHO2023	14.12.2023 VD	gid_E103_	2) Assoc w R - Interim	2	Glu103*	gid_p.Glu103*
4407843	CT	C	INDEL	CDS	Rv3919c	reverse	gid	E120fs	SM-R	WHO2023	14.12.2023 VD	gid_E120fs	2) Assoc w R - Interim	2	Glu120fs	gid_p.Glu120fs
4407839	AC	A	INDEL	CDS	Rv3919c	reverse	gid	E121_	SM-R	WHO2023	14.12.2023 VD	gid_E121_	2) Assoc w R - Interim	2	Glu121*	gid_p.Glu121*
4407839	AC	A	INDEL	CDS	Rv3919c	reverse	gid	E121fs	SM-R	WHO2023	14.12.2023 VD	gid_E121fs	2) Assoc w R - Interim	2	Glu121fs	gid_p.Glu121fs
4407708	CTC	TCA	SNP	CDS	Rv3919c	reverse	gid	E165_	SM-R	WHO2023	14.12.2023 VD	gid_E165_	2) Assoc w R - Interim	2	Glu165*	gid_p.Glu165*
4407693	TTC	CTT	SNP	CDS	Rv3919c	reverse	gid	E170_	SM-R	WHO2023	14.12.2023 VD	gid_E170_	2) Assoc w R - Interim	2	Glu170*	gid_p.Glu170*
4407684	CTC	TCA	SNP	CDS	Rv3919c	reverse	gid	E173_	SM-R	WHO2023	14.12.2023 VD	gid_E173_	2) Assoc w R - Interim	2	Glu173*	gid_p.Glu173*
4408105	CGC	GG	INDEL	CDS	Rv3919c	reverse	gid	E32fs	SM-R	WHO2023	14.12.2023 VD	gid_E32fs	2) Assoc w R - Interim	2	Glu32fs	gid_p.Glu32fs
4408083	TTC	CTA	SNP	CDS	Rv3919c	reverse	gid	E40_	SM-R	WHO2023	14.12.2023 VD	gid_E40_	2) Assoc w R - Interim	2	Glu40*	gid_p.Glu40*
4408022	GT	G	INDEL	CDS	Rv3919c	reverse	gid	E60_	SM-R	WHO2023	14.12.2023 VD	gid_E60_	2) Assoc w R - Interim	2	Glu60*	gid_p.Glu60*
4408022	GT	G	INDEL	CDS	Rv3919c	reverse	gid	E60fs	SM-R	WHO2023	14.12.2023 VD	gid_E60fs	2) Assoc w R - Interim	2	Glu60fs	gid_p.Glu60fs
4407599	CATTTCGTCAA	C	INDEL	CDS	Rv3919c	reverse	gid	E92fs	SM-R	WHO2023	14.12.2023 VD	gid_E92fs	2) Assoc w R - Interim	2	Glu92fs	gid_p.Glu92fs
4407906	CT	GC	SNP	CDS	Rv3919c	reverse	gid	E99_	SM-R	WHO2023	14.12.2023 VD	gid_E99_	1) Assoc w R	1	Glu99*	gid_p.Glu99*
4407906	C	CT	INDEL	CDS	Rv3919c	reverse	gid	F100fs	SM-R	WHO2023	14.12.2023 VD	gid_F100fs	2) Assoc w R - Interim	2	Phe100fs	gid_p.Phe100fs
4407876	GC	G	INDEL	CDS	Rv3919c	reverse	gid	G109fs	SM-R	WHO2023	14.12.2023 VD	gid_G109fs	2) Assoc w R - Interim	2	Gly109fs	gid_p.Gly109fs
4407536	ACTTCGTCGTG	T	INDEL	CDS	Rv3919c	reverse	gid	G117fs	SM-R	WHO2023	14.12.2023 VD	gid_G117fs	2) Assoc w R - Interim	2	Gly117fs	gid_p.Gly117fs
4407815	CGC	GG	INDEL	CDS	Rv3919c	reverse	gid	G129fs	SM-R	WHO2023	14.12.2023 VD	gid_G129fs	2) Assoc w R - Interim	2	Gly129fs	gid_p.Gly129fs
4407813	GCC	ACG	SNP	CDS	Rv3919c	reverse	gid	G13_	SM-R	WHO2023	14.12.2023 VD	gid_G13_	2) Assoc w R - Interim	2	Gly13*	gid_p.Gly13*
4407711	GC	G	INDEL	CDS	Rv3919c	reverse	gid	G164fs	SM-R	WHO2023	14.12.2023 VD	gid_G164fs	2) Assoc w R - Interim	2	Gly164fs	gid_p.Gly164fs
4407770	CTATCGCCAAT	A	INDEL	CDS	Rv3919c	reverse	gid	G17fs	SM-R	WHO2023	14.12.2023 VD	gid_G17fs	2) Assoc w R - Interim	2	Gly17fs	gid_p.Gly17fs
4407657	GC	G	INDEL	CDS	Rv3919c	reverse	gid	G182fs	SM-R	WHO2023	14.12.2023 VD	gid_G182fs	2) Assoc w R - Interim	2	Gly182fs	gid_p.Gly182fs
4407627	GCC	G	INDEL	CDS	Rv3919c	reverse	gid	G192fs	SM-R	WHO2023	14.12.2023 VD	gid_G192fs	2) Assoc w R - Interim	2	Gly192fs	gid_p.Gly192fs
4408119	TCC	ACG	SNP	CDS	Rv3919c	reverse	gid	G28_	SM-R	WHO2023	14.12.2023 VD	gid_G28_	2) Assoc w R - Interim	2	Gly28*	gid_p.Gly28*
4407630	GGCCACGCCCA	A	INDEL	CDS	Rv3919c	reverse	gid	G30fs	SM-R	WHO2023	14.12.2023 VD	gid_G30fs	2) Assoc w R - Interim	2	Gly30fs	gid_p.Gly30fs
4407567	CTGCACCCAGG	G	INDEL	CDS	Rv3919c	reverse	gid	G37_	SM-R	WHO2023	14.12.2023 VD	gid_G37_	2) Assoc w R - Interim	2	Gly37*	gid_p.Gly37*
4407567	CTGCACCCAGG	G	INDEL	CDS	Rv3919c	reverse	gid	G37fs	SM-R	WHO2023	14.12.2023 VD	gid_G37fs	2) Assoc w R - Interim	2	Gly37fs	gid_p.Gly37fs
4407999	G	GA	INDEL	CDS	Rv3919c	reverse	gid	G69fs	SM-R	WHO2023	14.12.2023 VD	gid_G69fs	2) Assoc w R - Interim	2	Gly69fs	gid_p.Gly69fs
4407983	ACCCGGCTC	A	INDEL	CDS	Rv3919c	reverse	gid	G71_	SM-R	WHO2023	14.12.2023 VD	gid_G71_	2) Assoc w R - Interim	2	Gly71*	gid_p.Gly71*
4407983	ACCCGGCTC	A	INDEL	CDS	Rv3919c	reverse	gid	G71fs	SM-R	WHO2023	14.12.2023 VD	gid_G71fs	2) Assoc w R - Interim	2	Gly71fs	gid_p.Gly71fs
4407984	CC	TT	SNP	CDS	Rv3919c	reverse	gid	G73E	SM-R	WHO2023	14.12.2023 VD	gid_G73E	1) Assoc w R	1	Gly73Glu	gid_p.Gly73Glu
4407975	GC	G	INDEL	CDS	Rv3919c	reverse	gid	G76fs	SM-R	WHO2023	14.12.2023 VD	gid_G76fs	2) Assoc w R - Interim	2	Gly76fs	gid_p.Gly76fs
4407698	CGTGA	C	INDEL	CDS	Rv3919c	reverse	gid	H168fs	SM-R	WHO2023	14.12.2023 VD	gid_H168fs	2) Assoc w R - Interim	2	His168fs	gid_p.His168fs
4407547	ACGCAAATAGT	G	INDEL	CDS	Rv3919c	reverse	gid	H174fs	SM-R	WHO2023	14.12.2023 VD	gid_H174fs	2) Assoc w R - Interim	2	His174fs	gid_p.His174fs
4407622	GACTCCTCGGC	T	INDEL	CDS	Rv3919c	reverse	gid	H48fs	SM-R	WHO2023	14.12.2023 VD	gid_H48fs	2) Assoc w R - Interim	2	His48fs	gid_p.His48fs
4408059	A	T	SNP	CDS	Rv3919c	reverse	gid	H48Q	SM-R	WHO2023	14.12.2023 VD	gid_H48Q	1) Assoc w R	1	His48Gln	gid_p.His48Gln
4407860	CG	C	INDEL	CDS	Rv3919c	reverse	gid	I114fs	SM-R	WHO2023	14.12.2023 VD	gid_I114fs	2) Assoc w R - Interim	2	Ile114fs	gid_p.Ile114fs
4407806	CCCGGCTCCGC	C	INDEL	CDS	Rv3919c	reverse	gid	I11fs	SM-R	WHO2023	14.12.2023 VD	gid_I11fs	2) Assoc w R - Interim	2	Ile11fs	gid_p.Ile11fs

4407665	CGA	C	INDEL	CDS	Rv3919c	reverse	gid	I179fs	SM-R	WHO2023	14.12.2023 VD	gid_I179fs	2) Assoc w R - Interim	2	Ile179fs	gid_p.Ile179fs
4407708	CACCCAGGACT	C	INDEL	CDS	Rv3919c	reverse	gid	I81fs	SM-R	WHO2023	14.12.2023 VD	gid_I81fs	2) Assoc w R - Interim	2	Ile81fs	gid_p.Ile81fs
4407771	CTT	TCA	SNP	CDS	Rv3919c	reverse	gid	K144_	SM-R	WHO2023	14.12.2023 VD	gid_K144_	2) Assoc w R - Interim	2	Lys144*	gid_p.Lys144*
4407761	AT	A	INDEL	CDS	Rv3919c	reverse	gid	K147fs	SM-R	WHO2023	14.12.2023 VD	gid_K147fs	2) Assoc w R - Interim	2	Lys147fs	gid_p.Lys147fs
4407714	TTT	CTA	SNP	CDS	Rv3919c	reverse	gid	K163_	SM-R	WHO2023	14.12.2023 VD	gid_K163_	2) Assoc w R - Interim	2	Lys163*	gid_p.Lys163*
4407879	CA	C	INDEL	CDS	Rv3919c	reverse	gid	L108fs	SM-R	WHO2023	14.12.2023 VD	gid_L108fs	2) Assoc w R - Interim	2	Leu108fs	gid_p.Leu108fs
4407747	CA	C	INDEL	CDS	Rv3919c	reverse	gid	L152fs	SM-R	WHO2023	14.12.2023 VD	gid_L152fs	2) Assoc w R - Interim	2	Leu152fs	gid_p.Leu152fs
4407748	TCCGGCCGCGG	A	INDEL	CDS	Rv3919c	reverse	gid	L16fs	SM-R	WHO2023	14.12.2023 VD	gid_L16fs	2) Assoc w R - Interim	2	Leu16fs	gid_p.Leu16fs
4407637	CAGATCTGTCA	A	INDEL	CDS	Rv3919c	reverse	gid	L26_	SM-R	WHO2023	14.12.2023 VD	gid_L26_	2) Assoc w R - Interim	2	Leu26*	gid_p.Leu26*
4407637	CAGATCTGTCA	A	INDEL	CDS	Rv3919c	reverse	gid	L26fs	SM-R	WHO2023	14.12.2023 VD	gid_L26fs	2) Assoc w R - Interim	2	Leu26fs	gid_p.Leu26fs
4408098	CA	C	INDEL	CDS	Rv3919c	reverse	gid	L35fs	SM-R	WHO2023	14.12.2023 VD	gid_L35fs	2) Assoc w R - Interim	2	Leu35fs	gid_p.Leu35fs
4407622	GGACTCCTCG	T	INDEL	CDS	Rv3919c	reverse	gid	L50fs	SM-R	WHO2023	14.12.2023 VD	gid_L50fs	2) Assoc w R - Interim	2	Leu50fs	gid_p.Leu50fs
4408027	A	GCTCACCGATC	INDEL	CDS	Rv3919c	reverse	gid	L59fs	SM-R	WHO2023	14.12.2023 VD	gid_L59fs	2) Assoc w R - Interim	2	Leu59fs	gid_p.Leu59fs
4407949	TCGCCAATGG	T	INDEL	CDS	Rv3919c	reverse	gid	L74_	SM-R	WHO2023	14.12.2023 VD	gid_L74_	2) Assoc w R - Interim	2	Leu74*	gid_p.Leu74*
4407949	TCGCCAATGG	T	INDEL	CDS	Rv3919c	reverse	gid	L74fs	SM-R	WHO2023	14.12.2023 VD	gid_L74fs	2) Assoc w R - Interim	2	Leu74fs	gid_p.Leu74fs
4407921	TACCTGGAGGT	T	INDEL	CDS	Rv3919c	reverse	gid	L79_	SM-R	WHO2023	14.12.2023 VD	gid_L79_	2) Assoc w R - Interim	2	Leu79*	gid_p.Leu79*
4407921	TACCTGGAGGT	T	INDEL	CDS	Rv3919c	reverse	gid	L79fs	SM-R	WHO2023	14.12.2023 VD	gid_L79fs	2) Assoc w R - Interim	2	Leu79fs	gid_p.Leu79fs
4407944	GGA	G	INDEL	CDS	Rv3919c	reverse	gid	L86fs	SM-R	WHO2023	14.12.2023 VD	gid_L86fs	2) Assoc w R - Interim	2	Leu86fs	gid_p.Leu86fs
4407932	GGA	G	INDEL	CDS	Rv3919c	reverse	gid	L90fs	SM-R	WHO2023	14.12.2023 VD	gid_L90fs	2) Assoc w R - Interim	2	Leu90fs	gid_p.Leu90fs
4407906	GGCGCAGTAGC	C	INDEL	CDS	Rv3919c	reverse	gid	L91fs	SM-R	WHO2023	14.12.2023 VD	gid_L91fs	2) Assoc w R - Interim	2	Leu91fs	gid_p.Leu91fs
4407918	CA	C	INDEL	CDS	Rv3919c	reverse	gid	L95fs	SM-R	WHO2023	14.12.2023 VD	gid_L95fs	2) Assoc w R - Interim	2	Leu95fs	gid_p.Leu95fs
4407529	C	T	SNP	CDS	Rv3919c	reverse	gid	LoF	SM-R	WHO2023	14.12.2023 VD	gid_LoF	1) Assoc w R	1	LoF	gid_p.LoF
4407589	TCTAGGAGAAC	G	INDEL	CDS	Rv3919c	reverse	gid	M1?	SM-R	WHO2023	14.12.2023 VD	gid_M1?	2) Assoc w R - Interim	2	Met1?	gid_p.Met1?
4407891	CAT	C	INDEL	CDS	Rv3919c	reverse	gid	M104fs	SM-R	WHO2023	14.12.2023 VD	gid_M104fs	2) Assoc w R - Interim	2	Met104fs	gid_p.Met104fs
4407724	AGCATTCGC	A	INDEL	CDS	Rv3919c	reverse	gid	M159fs	SM-R	WHO2023	14.12.2023 VD	gid_M159fs	2) Assoc w R - Interim	2	Met159fs	gid_p.Met159fs
4407672	C	CT	INDEL	CDS	Rv3919c	reverse	gid	M178fs	SM-R	WHO2023	14.12.2023 VD	gid_M178fs	2) Assoc w R - Interim	2	Met178fs	gid_p.Met178fs
4407584	CGGTCGCGGG	G	INDEL	CDS	Rv3919c	reverse	gid	N194fs	SM-R	WHO2023	14.12.2023 VD	gid_N194fs	2) Assoc w R - Interim	2	Asn194fs	gid_p.Asn194fs
4407822	GCTCCGCTACCC	T	INDEL	CDS	Rv3919c	reverse	gid	P14fs	SM-R	WHO2023	14.12.2023 VD	gid_P14fs	2) Assoc w R - Interim	2	Pro14fs	gid_p.Pro14fs
4407750	CG	C	INDEL	CDS	Rv3919c	reverse	gid	P151fs	SM-R	WHO2023	14.12.2023 VD	gid_P151fs	2) Assoc w R - Interim	2	Pro151fs	gid_p.Pro151fs
4407741	GTTCTAGGAGA	C	INDEL	CDS	Rv3919c	reverse	gid	P29fs	SM-R	WHO2023	14.12.2023 VD	gid_P29fs	2) Assoc w R - Interim	2	Pro29fs	gid_p.Pro29fs
4408090	G	GGTAT	INDEL	CDS	Rv3919c	reverse	gid	P38fs	SM-R	WHO2023	14.12.2023 VD	gid_P38fs	2) Assoc w R - Interim	2	Pro38fs	gid_p.Pro38fs
4407599	TGTCCAACGC	C	INDEL	CDS	Rv3919c	reverse	gid	P84fs	SM-R	WHO2023	14.12.2023 VD	gid_P84fs	2) Assoc w R - Interim	2	Pro84fs	gid_p.Pro84fs
4407926	G	GTTCTA	INDEL	CDS	Rv3919c	reverse	gid	P93fs	SM-R	WHO2023	14.12.2023 VD	gid_P93fs	2) Assoc w R - Interim	2	Pro93fs	gid_p.Pro93fs
4407828	CTG	TCA	SNP	CDS	Rv3919c	reverse	gid	Q125_	SM-R	WHO2023	14.12.2023 VD	gid_Q125_	1) Assoc w R	1	Gln125*	gid_p.Gln125*
4407828	CT	C	INDEL	CDS	Rv3919c	reverse	gid	Q125fs	SM-R	WHO2023	14.12.2023 VD	gid_Q125fs	2) Assoc w R - Interim	2	Gln125fs	gid_p.Gln125fs
4407822	TTG	CTC	SNP	CDS	Rv3919c	reverse	gid	Q127_	SM-R	WHO2023	14.12.2023 VD	gid_Q127_	2) Assoc w R - Interim	2	Gln127*	gid_p.Gln127*
4407570	GATCT	G	INDEL	CDS	Rv3919c	reverse	gid	Q210fs	SM-R	WHO2023	14.12.2023 VD	gid_Q210fs	2) Assoc w R - Interim	2	Gln210fs	gid_p.Gln210fs
4407761	GCGCGCCCCCG	A	INDEL	CDS	Rv3919c	reverse	gid	Q87fs	SM-R	WHO2023	14.12.2023 VD	gid_Q87fs	2) Assoc w R - Interim	2	Gln87fs	gid_p.Gln87fs
4407893	TCTCTCGA	T	INDEL	CDS	Rv3919c	reverse	gid	R102_	SM-R	WHO2023	14.12.2023 VD	gid_R102_	2) Assoc w R - Interim	2	Arg102*	gid_p.Arg102*
4407893	TCTCTCGA	T	INDEL	CDS	Rv3919c	reverse	gid	R102fs	SM-R	WHO2023	14.12.2023 VD	gid_R102fs	2) Assoc w R - Interim	2	Arg102fs	gid_p.Arg102fs
4407855	CC	G	INDEL	CDS	Rv3919c	reverse	gid	R116fs	SM-R	WHO2023	14.12.2023 VD	gid_R116fs	2) Assoc w R - Interim	2	Arg116fs	gid_p.Arg116fs
4407839	CTCCTCGGCG	A	INDEL	CDS	Rv3919c	reverse	gid	R118fs	SM-R	WHO2023	14.12.2023 VD	gid_R118fs	2) Assoc w R - Interim	2	Arg118fs	gid_p.Arg118fs
4407729	TC	AG	SNP	CDS	Rv3919c	reverse	gid	R158_	SM-R	WHO2023	14.12.2023 VD	gid_R158_	2) Assoc w R - Interim	2	Arg158*	gid_p.Arg158*
4407731	G	GC	INDEL	CDS	Rv3919c	reverse	gid	R158fs	SM-R	WHO2023	14.12.2023 VD	gid_R158fs	2) Assoc w R - Interim	2	Arg158fs	gid_p.Arg158fs
4408160	GC	G	INDEL	CDS	Rv3919c	reverse	gid	R15fs	SM-R	WHO2023	14.12.2023 VD	gid_R15fs	2) Assoc w R - Interim	2	Arg15fs	gid_p.Arg15fs
4407677	GC	G	INDEL	CDS	Rv3919c	reverse	gid	R176fs	SM-R	WHO2023	14.12.2023 VD	gid_R176fs	2) Assoc w R - Interim	2	Arg176fs	gid_p.Arg176fs
4407582	ACGTC	A	INDEL	CDS	Rv3919c	reverse	gid	R206_	SM-R	WHO2023	14.12.2023 VD	gid_R206_	2) Assoc w R - Interim	2	Arg206*	gid_p.Arg206*
4407582	ACGTC	A	INDEL	CDS	Rv3919c	reverse	gid	R206fs	SM-R	WHO2023	14.12.2023 VD	gid_R206fs	2) Assoc w R - Interim	2	Arg206fs	gid_p.Arg206fs
4407564	TCG	CTA	SNP	CDS	Rv3919c	reverse	gid	R213_	SM-R	WHO2023	14.12.2023 VD	gid_R213_	2) Assoc w R - Interim	2	Arg213*	gid_p.Arg213*
4408141	C	CG	INDEL	CDS	Rv3919c	reverse	gid	R21fs	SM-R	WHO2023	14.12.2023 VD	gid_R21fs	2) Assoc w R - Interim	2	Arg21fs	gid_p.Arg21fs
4407630	CAACGGCCACC	A	INDEL	CDS	Rv3919c	reverse	gid	R33fs	SM-R	WHO2023	14.12.2023 VD	gid_R33fs	2) Assoc w R - Interim	2	Arg33fs	gid_p.Arg33fs
4408086	GC	G	INDEL	CDS	Rv3919c	reverse	gid	R39fs	SM-R	WHO2023	14.12.2023 VD	gid_R39fs	1) Assoc w R	1	Arg39fs	gid_p.Arg39fs
4407734	AAACTCGGTGG	C	INDEL	CDS	Rv3919c	reverse	gid	R43fs	SM-R	WHO2023	14.12.2023 VD	gid_R43fs	2) Assoc w R - Interim	2	Arg43fs	gid_p.Arg43fs
4408002	ATCCACGACCC	A	INDEL	CDS	Rv3919c	reverse	gid	R64fs	SM-R	WHO2023	14.12.2023 VD	gid_R64fs	2) Assoc w R - Interim	2	Arg64fs	gid_p.Arg64fs
4407599	TCCAACGCGGC	C	INDEL	CDS	Rv3919c	reverse	gid	R83fs	SM-R	WHO2023	14.12.2023 VD	gid_R83fs	2) Assoc w R - Interim	2	Arg83fs	gid_p.Arg83fs
4407914	G	GGC	INDEL	CDS	Rv3919c	reverse	gid	R97fs	SM-R	WHO2023	14.12.2023 VD	gid_R97fs	2) Assoc w R - Interim	2	Arg97fs	gid_p.Arg97fs
4407839	A	AC	INDEL	CDS	Rv3919c	reverse	gid	S122fs	SM-R	WHO2023	14.12.2023 VD	gid_S122fs	2) Assoc w R - Interim	2	Ser122fs	gid_p.Ser122fs
4407795	TG	CT	SNP	CDS	Rv3919c	reverse	gid	S136_	SM-R	WHO2023	14.12.2023 VD	gid_S136_	2) Assoc w R - Interim	2	Ser136*	gid_p.Ser136*
4407796	GA	G	INDEL	CDS	Rv3919c	reverse	gid	S136fs	SM-R	WHO2023	14.12.2023 VD	gid_S136fs	2) Assoc w R - Interim	2	Ser136fs	gid_p.Ser136fs
4407753	CATGCT	C	INDEL	CDS	Rv3919c	reverse	gid	S149fs	SM-R	WHO2023	14.12.2023 VD	gid_S149fs	2) Assoc w R - Interim	2	Ser149fs	gid_p.Ser149fs
4407660	CGA	TAG	SNP	CDS	Rv3919c	reverse	gid	S181_	SM-R	WHO2023	14.12.2023 VD	gid_S181_	2) Assoc w R - Interim	2	Ser181*	gid_p.Ser181*
4407543	A	AC	INDEL	CDS	Rv3919c	reverse	gid	S220fs	SM-R	WHO2023	14.12.2023 VD	gid_S220fs	2) Assoc w R - Interim	2	Ser220fs	gid_p.Ser220fs
4407993	GC	AT	SNP	CDS	Rv3919c	reverse	gid	S70N	SM-R	WHO2023	14.12.2023 VD	gid_S70N	1) Assoc w R	1	Ser70Asn	gid_p.Ser70Asn
4407885	TG	T	INDEL	CDS	Rv3919c	reverse	gid	T106fs	SM-R	WHO2023	14.12.2023 VD	gid_T106fs	2) Assoc w R - Interim	2	Thr106fs	gid_p.Thr106fs
4407599	CGG	C	INDEL	CDS	Rv3919c	reverse	gid	T201fs	SM-R	WHO2023	14.12.2023 VD	gid_T201fs	2) Assoc w R - Interim	2	Thr201fs	gid_p.Thr201fs
4407582	CGTATCAACG	A	INDEL	CDS	Rv3919c	reverse	gid	T98fs	SM-R	WHO2023	14.12.2023 VD	gid_T98fs	2) Assoc w R - Interim	2	Thr98fs	gid_p.Thr98fs
4407888	CA	TT	SNP	CDS	Rv3919c	reverse	gid	V105E	SM-R	WHO2023	14.12.2023 VD	gid_V105E	1) Assoc w R	1	Val105Glu	gid_p.Val105Glu
4407887	TCA	T	INDEL	CDS	Rv3919c	reverse	gid	V105fs	SM-R	WHO2023	14.12.2023 VD	gid_V105fs	2) Assoc w R - Interim	2	Val105fs	gid_p.Val105fs
4407788	TCCTGCACCCA	C	INDEL	CDS	Rv3919c	reverse	gid	V110fs	SM-R	WHO2023	14.12.2023 VD	gid_V110fs	2) Assoc w R - Interim	2	Val110fs	gid_p.Val110fs
4407713	CGGCCACCGCC	C	INDEL	CDS	Rv3919c	reverse	gid	V115fs	SM-R	WHO2023	14.12.2023 VD	gid_V115fs	2) Assoc w R - Interim	2	Val115fs	gid_p.Val115fs
4407831	CA	C	INDEL	CDS	Rv3919c	reverse	gid	V124fs	SM-R	WHO2023	14.12.2023 VD	gid_V124fs	2) Assoc w R - Interim	2	Val124fs	gid_p.Val124fs
4407789	CGCCCGTGACA	C	INDEL	CDS	Rv3919c	reverse	gid	V135fs	SM-R	WHO2023	14.12.2023 VD	gid_V135fs	2) Assoc w R - Interim	2	Val135fs	gid_p.Val135fs
4407652	AC	A	INDEL	CDS	Rv3919c	reverse	gid	V184fs	SM-R	WHO2023	14.12.2023 VD	gid_V184fs	2) Assoc w R - Interim	2	Val184fs	gid_p.Val184fs
4407639	CA	C	INDEL	CDS	Rv3919c	reverse	gid	V188fs	SM-R	WHO2023	14.12.2023 VD	gid_V188fs	2) Assoc w R - Interim	2	Val188fs	gid_p.Val188fs
4407636	CA	C	INDEL	CDS	Rv3919c	reverse	gid	V189fs	SM-R	WHO2023	14.12.2023 VD	gid_V189fs	2) Assoc w R - Interim	2	Val189fs	gid_p.Val189fs
4407596	CCA	C	INDEL	CDS	Rv3919c	reverse	gid	V202fs	SM-R	WHO2023	14.12.2023 VD	gid_V202fs	2) Assoc w R - Interim	2	Val202fs	gid_p.Val202fs
4407705	AGAAACTCGGT	C	INDEL	CDS	Rv3919c	reverse	gid	V36fs	SM-R	WHO2023	14.12.2023 VD	gid_V36fs	2) Assoc w R - Interim	2	Val36fs	gid_p.Val36fs
4408082	C	CT	INDEL	CDS	Rv3919c	reverse	gid	V41fs	SM-R	WHO2023	14.12.2023 VD	gid_V41fs	2) Assoc w R - Interim	2	Val41fs	gid_p.Val41fs
4407649	TCGGCGCGCCC	T	INDEL	CDS	Rv3919c	reverse	gid	V54fs	SM-R	WHO2023	14.12.2023 VD	gid_V54fs	2) Assoc w R - Interim	2	Val54fs	gid_p.Val54fs
4407993	ACCGATATCCA	G	INDEL	CDS	Rv3919c	reverse	gid	V65fs	SM-R	WHO2023	14.12.2023 VD	gid_V65fs	2) Assoc w R - Interim	2	Val65fs	gid_p.Val65fs
4407999	GATATCCA	G	INDEL	CDS	Rv3919c	reverse	gid	V66fs	SM-R	WHO2023	14.12.2023 VD	gid_V66fs	2) Assoc w R - Interim	2	Val66fs	gid_p.Val66fs
4407546	CTCCATTTCGT	C	INDEL	CDS	Rv3919c	reverse	gid	V77fs	SM-R	WHO2023	14.12.2023 VD	gid_V77fs	2) Assoc w R - Interim	2	Val77fs	gid_p.Val77fs
4407937	ACTAC	A	INDEL	CDS	Rv3919c	reverse	gid	V88fs	SM-R	WHO2023	14.12.2023 VD	gid_V88fs	2) Assoc w R - Interim	2	Val88fs	gid_p.Val88fs
4407834	C	A	SNP	CDS	Rv3919c	reverse	gid	W123_	SM-R	WHO2023	14.12.2023 VD	gid_W123_	2) Assoc w R - Interim	2	Trp123*	gid_p.Trp123*
4407692	CGAGCATTCGC	C	INDEL	CDS	Rv3919c	reverse	gid	W148_	SM-R	WHO2023	14.12.2023 VD	gid_W148_	2) Assoc w R - Interim	2	Trp148*	gid_p.Trp148*

4407692	GAGCATTCGC	C	INDEL	CDS	Rv3919c	reverse	gid	W148fs	SM-R	WHO2023	14.12.2023 VD	gid_W148fs	2) Assoc w R - Interim	2	Trp148fs	gid_p.Trp148fs
4408068	CCA	ACC	SNP	CDS	Rv3919c	reverse	gid	W45_	SM-R	WHO2023	14.12.2023 VD	gid_W45_	2) Assoc w R - Interim	2	Trp45*	gid_p.Trp45*
4408137	GT	CG	SNP	CDS	Rv3919c	reverse	gid	Y22_	SM-R	WHO2023	14.12.2023 VD	gid_Y22_	2) Assoc w R - Interim	2	Tyr22*	gid_p.Tyr22*
6735	A	C	SNP	CDS	Rv0005	forward	gyrB	N499T	LFX-R, MFX-R	WHO2023	14.12.2023 VD	gyrB_N499T	1) Assoc w R	1	Asn499Thr	gyrB_p.Asn499Thr
6579	CC	TT	SNP	PROM	Rv0005	forward	gyrB	S447F	LFX-R;MFX-R	WHO2023	14.12.2023 VD	gyrB_S447F	2) Assoc w R - Interim	2	Ser447Phe	gyrB_c.Ser447Phe
1674048	G	A	SNP	PROM	Rv1484	forward	inhA	-154G>A	INH-R, ETH-R	WHO2023	14.12.2023 VD	inhA_-154G>A	1) Assoc w R	1	-154G>A	inhA_c.-154G>A
1673432	T	A	SNP	PROM	Rv1484	forward	inhA	-770T>A	INH-R, ETH-R	WHO2023	14.12.2023 VD	inhA_-770T>A	2) Assoc w R - Interim	2	-770T>A	inhA_c.-770T>A
1673432	T	C	SNP	PROM	Rv1484	forward	inhA	-770T>C	INH-R, ETH-R	WHO2023	14.12.2023 VD	inhA_-770T>C	2) Assoc w R - Interim	2	-770T>C	inhA_c.-770T>C
1673432	T	G	SNP	PROM	Rv1484	forward	inhA	-770T>G	INH-R, ETH-R	WHO2023	14.12.2023 VD	inhA_-770T>G	2) Assoc w R - Interim	2	-770T>G	inhA_c.-770T>G
1673423	GAC	TAT	SNP	PROM	Rv1484	forward	inhA	-777C>T	INH-R, ETH-R	WHO2023	14.12.2023 VD	inhA_-777C>T	1) Assoc w R	1	-777C>T	inhA_c.-777C>T
1673424	A	G	SNP	PROM	Rv1484	forward	inhA	-778A>G	INH-R, ETH-R	WHO2023	14.12.2023 VD	inhA_-778A>G	2) Assoc w R - Interim	2	-778A>G	inhA_c.-778A>G
1673423	G	T	SNP	PROM	Rv1484	forward	inhA	-779G>T	INH-R, ETH-R	WHO2023	14.12.2023 VD	inhA_-779G>T	2) Assoc w R - Interim	2	-779G>T	inhA_c.-779G>T
2156064	AG	A	INDEL	CDS	Rv1908c	reverse	katG	A16fs	INH-R	WHO2023	14.12.2023 VD	katG_A16fs	2) Assoc w R - Interim	2	Ala16fs	katG_p.Ala16fs
2155015	GACCAGATCG	T	INDEL	CDS	Rv1908c	reverse	katG	A202fs	INH-R	WHO2023	14.12.2023 VD	katG_A202fs	2) Assoc w R - Interim	2	Ala202fs	katG_p.Ala202fs
2154679	GC	G	INDEL	CDS	Rv1908c	reverse	katG	A478fs	INH-R	WHO2023	14.12.2023 VD	katG_A478fs	2) Assoc w R - Interim	2	Ala478fs	katG_p.Ala478fs
2154122	GTACTCGGCC	T	INDEL	CDS	Rv1908c	reverse	katG	A551fs	INH-R	WHO2023	14.12.2023 VD	katG_A551fs	2) Assoc w R - Interim	2	Ala551fs	katG_p.Ala551fs
2154431	GGCCAGCC	G	INDEL	CDS	Rv1908c	reverse	katG	A559fs	INH-R	WHO2023	14.12.2023 VD	katG_A559fs	2) Assoc w R - Interim	2	Ala559fs	katG_p.Ala559fs
2154359	C	CA	INDEL	CDS	Rv1908c	reverse	katG	A585fs	INH-R	WHO2023	14.12.2023 VD	katG_A585fs	2) Assoc w R - Interim	2	Ala585fs	katG_p.Ala585fs
2155599	GCA	CCC	SNP	CDS	Rv1908c	reverse	katG	C171_	INH-R	WHO2023	14.12.2023 VD	katG_C171_	2) Assoc w R - Interim	2	Cys171*	katG_p.Cys171*
2155538	ACGCCATCCG	C	INDEL	CDS	Rv1908c	reverse	katG	C20fs	INH-R	WHO2023	14.12.2023 VD	katG_C20fs	2) Assoc w R - Interim	2	Cys20fs	katG_p.Cys20fs
2155626	C	CG	INDEL	CDS	Rv1908c	reverse	katG	D163fs	INH-R	WHO2023	14.12.2023 VD	katG_D163fs	2) Assoc w R - Interim	2	Asp163fs	katG_p.Asp163fs
2155003	TGCCATACGA	C	INDEL	CDS	Rv1908c	reverse	katG	D240fs	INH-R	WHO2023	14.12.2023 VD	katG_D240fs	2) Assoc w R - Interim	2	Asp240fs	katG_p.Asp240fs
2154383	GCTTCGCCG	C	INDEL	CDS	Rv1908c	reverse	katG	D329fs	INH-R	WHO2023	14.12.2023 VD	katG_D329fs	2) Assoc w R - Interim	2	Asp329fs	katG_p.Asp329fs
2154969	G	GT	INDEL	CDS	Rv1908c	reverse	katG	D381fs	INH-R	WHO2023	14.12.2023 VD	katG_D381fs	2) Assoc w R - Interim	2	Asp381fs	katG_p.Asp381fs
2154573	A	AT	INDEL	CDS	Rv1908c	reverse	katG	D513fs	INH-R	WHO2023	14.12.2023 VD	katG_D513fs	2) Assoc w R - Interim	2	Asp513fs	katG_p.Asp513fs
2155562	CCGCGGCCG	C	INDEL	CDS	Rv1908c	reverse	katG	D56fs	INH-R	WHO2023	14.12.2023 VD	katG_D56fs	2) Assoc w R - Interim	2	Asp56fs	katG_p.Asp56fs
2154135	ACGAAGAAG	C	INDEL	CDS	Rv1908c	reverse	katG	D656fs	INH-R	WHO2023	14.12.2023 VD	katG_D656fs	2) Assoc w R - Interim	2	Asp656fs	katG_p.Asp656fs
2154028	T	TCCACG	INDEL	CDS	Rv1908c	reverse	katG	D695fs	INH-R	WHO2023	14.12.2023 VD	katG_D695fs	2) Assoc w R - Interim	2	Asp695fs	katG_p.Asp695fs
2153926	TC	T	INDEL	CDS	Rv1908c	reverse	katG	D729fs	INH-R	WHO2023	14.12.2023 VD	katG_D729fs	2) Assoc w R - Interim	2	Asp729fs	katG_p.Asp729fs
#NV	#NV	#NV	#NV	CDS	Rv1908c	reverse	katG	deletion	INH-R	WHO2023	14.12.2023 VD	katG_deletion	2) Assoc w R - Interim	2	deletion	katG_p.deletion
2156076	GGTGGTTTC	G	INDEL	CDS	Rv1908c	reverse	katG	E10fs	INH-R	WHO2023	14.12.2023 VD	katG_E10fs	2) Assoc w R - Interim	2	Glu10fs	katG_p.Glu10fs
2155489	T	TGTTCGGG	INDEL	CDS	Rv1908c	reverse	katG	E208fs	INH-R	WHO2023	14.12.2023 VD	katG_E208fs	2) Assoc w R - Interim	2	Glu208fs	katG_p.Glu208fs
2155087	T	TC	INDEL	CDS	Rv1908c	reverse	katG	E342fs	INH-R	WHO2023	14.12.2023 VD	katG_E342fs	2) Assoc w R - Interim	2	Glu342fs	katG_p.Glu342fs
2156095	GGGTGTTGC	G	INDEL	CDS	Rv1908c	reverse	katG	E3fs	INH-R	WHO2023	14.12.2023 VD	katG_E3fs	2) Assoc w R - Interim	2	Glu3fs	katG_p.Glu3fs
2153960	GGAGGCCTC	G	INDEL	CDS	Rv1908c	reverse	katG	E582fs	INH-R	WHO2023	14.12.2023 VD	katG_E582fs	2) Assoc w R - Interim	2	Glu582fs	katG_p.Glu582fs
2154348	CT	GC	SNP	CDS	Rv1908c	reverse	katG	E588_	INH-R	WHO2023	14.12.2023 VD	katG_E588_	2) Assoc w R - Interim	2	Glu588*	katG_p.Glu588*
2154291	CT	AC	SNP	CDS	Rv1908c	reverse	katG	E607_	INH-R	WHO2023	14.12.2023 VD	katG_E607_	2) Assoc w R - Interim	2	Glu607*	katG_p.Glu607*
2154244	T	TGC	INDEL	CDS	Rv1908c	reverse	katG	E623fs	INH-R	WHO2023	14.12.2023 VD	katG_E623fs	2) Assoc w R - Interim	2	Glu623fs	katG_p.Glu623fs
2154169	TC	T	INDEL	CDS	Rv1908c	reverse	katG	E648fs	INH-R	WHO2023	14.12.2023 VD	katG_E648fs	2) Assoc w R - Interim	2	Glu648fs	katG_p.Glu648fs
2154162	G	GT	INDEL	CDS	Rv1908c	reverse	katG	E651fs	INH-R	WHO2023	14.12.2023 VD	katG_E651fs	2) Assoc w R - Interim	2	Glu651fs	katG_p.Glu651fs
2154106	T	TC	INDEL	CDS	Rv1908c	reverse	katG	E669fs	INH-R	WHO2023	14.12.2023 VD	katG_E669fs	2) Assoc w R - Interim	2	Glu669fs	katG_p.Glu669fs
2155295	CG	C	INDEL	CDS	Rv1908c	reverse	katG	F272fs	INH-R	WHO2023	14.12.2023 VD	katG_F272fs	2) Assoc w R - Interim	2	Phe272fs	katG_p.Phe272fs
2154793	ATCGGCCGGG	T	INDEL	CDS	Rv1908c	reverse	katG	G124fs	INH-R	WHO2023	14.12.2023 VD	katG_G124fs	2) Assoc w R - Interim	2	Gly124fs	katG_p.Gly124fs
2155065	GGCCGGGCC	G	INDEL	CDS	Rv1908c	reverse	katG	G212fs	INH-R	WHO2023	14.12.2023 VD	katG_G212fs	2) Assoc w R - Interim	2	Gly212fs	katG_p.Gly212fs
2155308	GC	G	INDEL	CDS	Rv1908c	reverse	katG	G268fs	INH-R	WHO2023	14.12.2023 VD	katG_G268fs	2) Assoc w R - Interim	2	Gly268fs	katG_p.Gly268fs
2155274	GGC	G	INDEL	CDS	Rv1908c	reverse	katG	G279fs	INH-R	WHO2023	14.12.2023 VD	katG_G279fs	2) Assoc w R - Interim	2	Gly279fs	katG_p.Gly279fs
2156013	G	CCGCCCTCG	INDEL	CDS	Rv1908c	reverse	katG	G34fs	INH-R	WHO2023	14.12.2023 VD	katG_G34fs	2) Assoc w R - Interim	2	Gly34fs	katG_p.Gly34fs
2155038	GC	G	INDEL	CDS	Rv1908c	reverse	katG	G358fs	INH-R	WHO2023	14.12.2023 VD	katG_G358fs	2) Assoc w R - Interim	2	Gly358fs	katG_p.Gly358fs
2154664	AGGTATCTCG	A	INDEL	CDS	Rv1908c	reverse	katG	G369fs	INH-R	WHO2023	14.12.2023 VD	katG_G369fs	2) Assoc w R - Interim	2	Gly369fs	katG_p.Gly369fs
2154311	C	CT	INDEL	CDS	Rv1908c	reverse	katG	G601fs	INH-R	WHO2023	14.12.2023 VD	katG_G601fs	2) Assoc w R - Interim	2	Gly601fs	katG_p.Gly601fs
2156038	T	TGACCC	INDEL	CDS	Rv1908c	reverse	katG	H25fs	INH-R	WHO2023	14.12.2023 VD	katG_H25fs	2) Assoc w R - Interim	2	His25fs	katG_p.His25fs
2154912	G	GT	INDEL	CDS	Rv1908c	reverse	katG	H400fs	INH-R	WHO2023	14.12.2023 VD	katG_H400fs	2) Assoc w R - Interim	2	His400fs	katG_p.His400fs
2154064	AGGGGCACT	C	INDEL	CDS	Rv1908c	reverse	katG	H561fs	INH-R	WHO2023	14.12.2023 VD	katG_H561fs	2) Assoc w R - Interim	2	His561fs	katG_p.His561fs
2154758	CGTGATCCG	C	INDEL	CDS	Rv1908c	reverse	katG	I335fs	INH-R	WHO2023	14.12.2023 VD	katG_I335fs	2) Assoc w R - Interim	2	Ile335fs	katG_p.Ile335fs
2154934	A	ATG	INDEL	CDS	Rv1908c	reverse	katG	I393fs	INH-R	WHO2023	14.12.2023 VD	katG_I393fs	2) Assoc w R - Interim	2	Ile393fs	katG_p.Ile393fs
2154367	CGAATGACC	T	INDEL	CDS	Rv1908c	reverse	katG	I455fs	INH-R	WHO2023	14.12.2023 VD	katG_I455fs	2) Assoc w R - Interim	2	Ile455fs	katG_p.Ile455fs
2154504	GA	G	INDEL	CDS	Rv1908c	reverse	katG	I536fs	INH-R	WHO2023	14.12.2023 VD	katG_I536fs	2) Assoc w R - Interim	2	Ile536fs	katG_p.Ile536fs
2154423	GA	G	INDEL	CDS	Rv1908c	reverse	katG	I563fs	INH-R	WHO2023	14.12.2023 VD	katG_I563fs	2) Assoc w R - Interim	2	Ile563fs	katG_p.Ile563fs
2155538	CGGCCCGTAG	C	INDEL	CDS	Rv1908c	reverse	katG	I8fs	INH-R	WHO2023	14.12.2023 VD	katG_I8fs	2) Assoc w R - Interim	2	Ile8fs	katG_p.Ile8fs
2155638	CTTCTT	C	INDEL	CDS	Rv1908c	reverse	katG	K157fs	INH-R	WHO2023	14.12.2023 VD	katG_K157fs	2) Assoc w R - Interim	2	Lys157fs	katG_p.Lys157fs
2155575	CT	C	INDEL	CDS	Rv1908c	reverse	katG	K179fs	INH-R	WHO2023	14.12.2023 VD	katG_K179fs	2) Assoc w R - Interim	2	Lys179fs	katG_p.Lys179fs
2155511	CCT	C	INDEL	CDS	Rv1908c	reverse	katG	K200fs	INH-R	WHO2023	14.12.2023 VD	katG_K200fs	2) Assoc w R - Interim	2	Lys200fs	katG_p.Lys200fs
2154805	AGGGTCTGCT	A	INDEL	CDS	Rv1908c	reverse	katG	K433fs	INH-R	WHO2023	14.12.2023 VD	katG_K433fs	2) Assoc w R - Interim	2	Lys433fs	katG_p.Lys433fs
2154737	TA	T	INDEL	CDS	Rv1908c	reverse	katG	K459fs	INH-R	WHO2023	14.12.2023 VD	katG_K459fs	2) Assoc w R - Interim	2	Lys459fs	katG_p.Lys459fs
2155976	T	TA	INDEL	CDS	Rv1908c	reverse	katG	K46fs	INH-R	WHO2023	14.12.2023 VD	katG_K46fs	2) Assoc w R - Interim	2	Lys46fs	katG_p.Lys46fs
2154441	CT	C	INDEL	CDS	Rv1908c	reverse	katG	K557fs	INH-R	WHO2023	14.12.2023 VD	katG_K557fs	2) Assoc w R - Interim	2	Lys557fs	katG_p.Lys557fs
2154342	CT	C	INDEL	CDS	Rv1908c	reverse	katG	K590fs	INH-R	WHO2023	14.12.2023 VD	katG_K590fs	2) Assoc w R - Interim	2	Lys590fs	katG_p.Lys590fs
2155582	CATCGATTCC	C	INDEL	CDS	Rv1908c	reverse	katG	L173fs	INH-R	WHO2023	14.12.2023 VD	katG_L173fs	2) Assoc w R - Interim	2	Leu173fs	katG_p.Leu173fs
2154321	CCGCGCTTGT	G	INDEL	CDS	Rv1908c	reverse	katG	L382fs	INH-R	WHO2023	14.12.2023 VD	katG_L382fs	2) Assoc w R - Interim	2	Leu382fs	katG_p.Leu382fs
2154025	AG	A	INDEL	CDS	Rv1908c	reverse	katG	L696fs	INH-R	WHO2023	14.12.2023 VD	katG_L696fs	2) Assoc w R - Interim	2	Leu696fs	katG_p.Leu696fs
2152935	GTGGTCTGC	C	INDEL	CDS	Rv1908c	reverse	katG	LoF	INH-R	WHO2023	14.12.2023 VD	katG_LoF	1) Assoc w R	1	LoF	katG_p.LoF
2155557	CACCACGGC	G	INDEL	CDS	Rv1908c	reverse	katG	M1?	INH-R	WHO2023	14.12.2023 VD	katG_M1?	1) Assoc w R	1	Met1?	katG_p.Met1?
2156033	TC	T	INDEL	CDS	Rv1908c	reverse	katG	M26fs	INH-R	WHO2023	14.12.2023 VD	katG_M26fs	2) Assoc w R - Interim	2	Met26fs	katG_p.Met26fs
2154240	CA	C	INDEL	CDS	Rv1908c	reverse	katG	M624fs	INH-R	WHO2023	14.12.2023 VD	katG_M624fs	2) Assoc w R - Interim	2	Met624fs	katG_p.Met624fs
2155654	AGGTGGTCAT	T	INDEL	CDS	Rv1908c	reverse	katG	N18fs	INH-R	WHO2023	14.12.2023 VD	katG_N18fs	2) Assoc w R - Interim	2	Asn18fs	katG_p.Asn18fs
2155403	CG	C	INDEL	CDS	Rv1908c	reverse	katG	N236fs	INH-R	WHO2023	14.12.2023 VD	katG_N236fs	2) Assoc w R - Interim	2	Asn236fs	katG_p.Asn236fs
2156007	G	GT	INDEL	CDS	Rv1908c	reverse	katG	N35fs	INH-R	WHO2023	14.12.2023 VD	katG_N35fs	2) Assoc w R - Interim	2	Asn35fs	katG_p.Asn35fs
2155341	CTTCTTCTTGA	C	INDEL	CDS	Rv1908c	reverse	katG	N51fs	INH-R	WHO2023	14.12.2023 VD	katG_N51fs	2) Assoc w R - Interim	2	Asn51fs	katG_p.Asn51fs
2154032	GTTCGCCTTG	C	INDEL	CDS	Rv1908c	reverse	katG	N535fs	INH-R	WHO2023	14.12.2023 VD	katG_N535fs	2) Assoc w R - Interim	2	Asn535fs	katG_p.Asn535fs
2155137	CG	C	INDEL	CDS	Rv1908c	reverse	katG	P325fs	INH-R	WHO2023	14.12.2023 VD	katG_P325fs	2) Assoc w R - Interim	2	Pro325fs	katG_p.Pro325fs
2154342	ACCTCCCACCC	C	INDEL	CDS	Rv1908c	reverse	katG	P422fs	INH-R	WHO2023	14.12.2023 VD	katG_P422fs	2) Assoc w R - Interim	2	Pro422fs	katG_p.Pro422fs
2154350	CGTTGACCTC	C	INDEL	CDS	Rv1908c	reverse	katG	P429fs	INH-R	WHO2023	14.12.2023 VD	katG_P429fs	2) Assoc w R - Interim	2	Pro429fs	katG_p.Pro429fs
2153916	TCGGAGGCC	C	INDEL	CDS	Rv1908c	reverse	katG	P569fs	INH-R	WHO2023	14.12.2023 VD	katG_P569fs	2) Assoc w R - Interim	2	Pro569fs	katG_p.Pro569fs
2154246	AG	A	INDEL	CDS	Rv1908c	reverse	katG	P622fs	INH-R	WHO2023	14.12.2023 VD	katG_P622fs	2) Assoc w R - Interim	2	Pro622fs	katG_p.Pro622fs
2154192	G	GC	INDEL	CDS	Rv1908c	reverse	katG	P642fs	INH-R	WHO2023	14.12.2023 VD	katG_P642fs	2) Assoc w R - Interim	2	Pro642fs	katG_p.Pro642fs

2155529	CAGCGGCCCGT	C	INDEL	CDS	Rv1908c	reverse	katG	P6fs	INH-R	WHO2023	14.12.2023 VD	katG_P6fs	2) Assoc w R - Interim	2	Pro6fs	katG_p.Pro6fs
2156093	G	GT	INDEL	CDS	Rv1908c	reverse	katG	P7fs	INH-R	WHO2023	14.12.2023 VD	katG_P7fs	2) Assoc w R - Interim	2	Pro7fs	katG_p.Pro7fs
2155056	T	A	SNP	CDS	Rv1908c	reverse	katG	Q352_	INH-R	WHO2023	14.12.2023 VD	katG_Q352_	2) Assoc w R - Interim	2	Gln352*	katG_p.Gln352*
2154537	C	CA	INDEL	CDS	Rv1908c	reverse	katG	Q525fs	INH-R	WHO2023	14.12.2023 VD	katG_Q525fs	2) Assoc w R - Interim	2	Gln525fs	katG_p.Gln525fs
2154374	CGGTTT	C	INDEL	CDS	Rv1908c	reverse	katG	Q578fs	INH-R	WHO2023	14.12.2023 VD	katG_Q578fs	2) Assoc w R - Interim	2	Gln578fs	katG_p.Gln578fs
2154625	C	CG	INDEL	CDS	Rv1908c	reverse	katG	R496fs	INH-R	WHO2023	14.12.2023 VD	katG_R496fs	2) Assoc w R - Interim	2	Arg496fs	katG_p.Arg496fs
2155587	CG	TC	SNP	CDS	Rv1908c	reverse	katG	S175_	INH-R	WHO2023	14.12.2023 VD	katG_S175_	2) Assoc w R - Interim	2	Ser175*	katG_p.Ser175*
2155206	GC	G	INDEL	CDS	Rv1908c	reverse	katG	S302fs	INH-R	WHO2023	14.12.2023 VD	katG_S302fs	2) Assoc w R - Interim	2	Ser302fs	katG_p.Ser302fs
2155167	GCT	ACG	SNP	CDS	Rv1908c	reverse	katG	S315R	INH-R	WHO2023	14.12.2023 VD	katG_S315R	1) Assoc w R	1	Ser315Arg	katG_p.Ser315Arg
2154496	G	GAC	INDEL	CDS	Rv1908c	reverse	katG	S539fs	INH-R	WHO2023	14.12.2023 VD	katG_S539fs	2) Assoc w R - Interim	2	Ser539fs	katG_p.Ser539fs
2154163	G	GAGGCC	INDEL	CDS	Rv1908c	reverse	katG	S650fs	INH-R	WHO2023	14.12.2023 VD	katG_S650fs	2) Assoc w R - Interim	2	Ser650fs	katG_p.Ser650fs
2154157	G	GC	INDEL	CDS	Rv1908c	reverse	katG	S652fs	INH-R	WHO2023	14.12.2023 VD	katG_S652fs	2) Assoc w R - Interim	2	Ser652fs	katG_p.Ser652fs
2154099	CG	TT	SNP	CDS	Rv1908c	reverse	katG	S671_	INH-R	WHO2023	14.12.2023 VD	katG_S671_	2) Assoc w R - Interim	2	Ser671*	katG_p.Ser671*
2155505	TG	T	INDEL	CDS	Rv1908c	reverse	katG	T203fs	INH-R	WHO2023	14.12.2023 VD	katG_T203fs	2) Assoc w R - Interim	2	Thr203fs	katG_p.Thr203fs
2154556	GCTGGCAATCT	C	INDEL	CDS	Rv1908c	reverse	katG	T394fs	INH-R	WHO2023	14.12.2023 VD	katG_T394fs	2) Assoc w R - Interim	2	Thr394fs	katG_p.Thr394fs
2155610	CGAAAA	C	INDEL	CDS	Rv1908c	reverse	katG	V166fs	INH-R	WHO2023	14.12.2023 VD	katG_V166fs	2) Assoc w R - Interim	2	Val166fs	katG_p.Val166fs
2155635	GGCCACCACGG	G	INDEL	CDS	Rv1908c	reverse	katG	V22fs	INH-R	WHO2023	14.12.2023 VD	katG_V22fs	2) Assoc w R - Interim	2	Val22fs	katG_p.Val22fs
2155671	CCACGGCTGCC	C	INDEL	CDS	Rv1908c	reverse	katG	V30fs	INH-R	WHO2023	14.12.2023 VD	katG_V30fs	2) Assoc w R - Interim	2	Val30fs	katG_p.Val30fs
2154591	TTGTACCAGGC	G	INDEL	CDS	Rv1908c	reverse	katG	V319fs	INH-R	WHO2023	14.12.2023 VD	katG_V319fs	2) Assoc w R - Interim	2	Val319fs	katG_p.Val319fs
2154955	A	ACC	INDEL	CDS	Rv1908c	reverse	katG	V386fs	INH-R	WHO2023	14.12.2023 VD	katG_V386fs	2) Assoc w R - Interim	2	Val386fs	katG_p.Val386fs
2154385	CCCGACTTGTG	T	INDEL	CDS	Rv1908c	reverse	katG	V431fs	INH-R	WHO2023	14.12.2023 VD	katG_V431fs	2) Assoc w R - Interim	2	Val431fs	katG_p.Val431fs
2154778	A	AC	INDEL	CDS	Rv1908c	reverse	katG	V445fs	INH-R	WHO2023	14.12.2023 VD	katG_V445fs	2) Assoc w R - Interim	2	Val445fs	katG_p.Val445fs
2154559	ATGAC	A	INDEL	CDS	Rv1908c	reverse	katG	V517fs	INH-R	WHO2023	14.12.2023 VD	katG_V517fs	2) Assoc w R - Interim	2	Val517fs	katG_p.Val517fs
2154499	A	AC	INDEL	CDS	Rv1908c	reverse	katG	V538fs	INH-R	WHO2023	14.12.2023 VD	katG_V538fs	2) Assoc w R - Interim	2	Val538fs	katG_p.Val538fs
2154356	C	CG	INDEL	CDS	Rv1908c	reverse	katG	V586fs	INH-R	WHO2023	14.12.2023 VD	katG_V586fs	2) Assoc w R - Interim	2	Val586fs	katG_p.Val586fs
2155279	AACGTCTTGAA	G	INDEL	CDS	Rv1908c	reverse	katG	V83fs	INH-R	WHO2023	14.12.2023 VD	katG_V83fs	2) Assoc w R - Interim	2	Val83fs	katG_p.Val83fs
2155791	C	T	SNP	CDS	Rv1908c	reverse	katG	W107_	INH-R	WHO2023	14.12.2023 VD	katG_W107_	2) Assoc w R - Interim	2	Trp107*	katG_p.Trp107*
2155518	C	T	SNP	CDS	Rv1908c	reverse	katG	W198_	INH-R	WHO2023	14.12.2023 VD	katG_W198_	2) Assoc w R - Interim	2	Trp198*	katG_p.Trp198*
2155519	C	CACAA	INDEL	CDS	Rv1908c	reverse	katG	W198fs	INH-R	WHO2023	14.12.2023 VD	katG_W198fs	2) Assoc w R - Interim	2	Trp198fs	katG_p.Trp198fs
2155500	C	T	SNP	CDS	Rv1908c	reverse	katG	W204_	INH-R	WHO2023	14.12.2023 VD	katG_W204_	2) Assoc w R - Interim	2	Trp204*	katG_p.Trp204*
2155149	C	T	SNP	CDS	Rv1908c	reverse	katG	W321_	INH-R	WHO2023	14.12.2023 VD	katG_W321_	2) Assoc w R - Interim	2	Trp321*	katG_p.Trp321*
2155054	TATTGCCA	T	INDEL	CDS	Rv1908c	reverse	katG	W351_	INH-R	WHO2023	14.12.2023 VD	katG_W351_	2) Assoc w R - Interim	2	Trp351*	katG_p.Trp351*
2155054	TATTGCCA	T	INDEL	CDS	Rv1908c	reverse	katG	W351fs	INH-R	WHO2023	14.12.2023 VD	katG_W351fs	2) Assoc w R - Interim	2	Trp351fs	katG_p.Trp351fs
2154921	CC	TT	SNP	CDS	Rv1908c	reverse	katG	W39_	INH-R	WHO2023	14.12.2023 VD	katG_W39_	2) Assoc w R - Interim	2	Trp39*	katG_p.Trp39*
2154921	CC	TT	SNP	CDS	Rv1908c	reverse	katG	W397_	INH-R	WHO2023	14.12.2023 VD	katG_W397_	2) Assoc w R - Interim	2	Trp397*	katG_p.Trp397*
2155677	CGGCGGGCCAC	G	INDEL	CDS	Rv1908c	reverse	katG	W39fs	INH-R	WHO2023	14.12.2023 VD	katG_W39fs	2) Assoc w R - Interim	2	Trp39fs	katG_p.Trp39fs
2154798	C	A	SNP	CDS	Rv1908c	reverse	katG	W438_	INH-R	WHO2023	14.12.2023 VD	katG_W438_	2) Assoc w R - Interim	2	Trp438*	katG_p.Trp438*
2154681	CC	TT	SNP	CDS	Rv1908c	reverse	katG	W477_	INH-R	WHO2023	14.12.2023 VD	katG_W477_	2) Assoc w R - Interim	2	Trp477*	katG_p.Trp477*
2154599	AC	A	INDEL	CDS	Rv1908c	reverse	katG	W505fs	INH-R	WHO2023	14.12.2023 VD	katG_W505fs	2) Assoc w R - Interim	2	Trp505fs	katG_p.Trp505fs
2154109	C	CA	INDEL	CDS	Rv1908c	reverse	katG	W668fs	INH-R	WHO2023	14.12.2023 VD	katG_W668fs	2) Assoc w R - Interim	2	Trp668fs	katG_p.Trp668fs
2154045	C	CCA	INDEL	CDS	Rv1908c	reverse	katG	W689fs	INH-R	WHO2023	14.12.2023 VD	katG_W689fs	2) Assoc w R - Interim	2	Trp689fs	katG_p.Trp689fs
2153928	CC	AT	SNP	CDS	Rv1908c	reverse	katG	W728_	INH-R	WHO2023	14.12.2023 VD	katG_W728_	2) Assoc w R - Interim	2	Trp728*	katG_p.Trp728*
2155773	G	C	SNP	CDS	Rv1908c	reverse	katG	Y113_	INH-R	WHO2023	14.12.2023 VD	katG_Y113_	2) Assoc w R - Interim	2	Tyr113*	katG_p.Tyr113*
2155647	GTA	ATC	SNP	CDS	Rv1908c	reverse	katG	Y155_	INH-R	WHO2023	14.12.2023 VD	katG_Y155_	2) Assoc w R - Interim	2	Tyr155*	katG_p.Tyr155*
2154988	CGGGGCCGACC	G	INDEL	CDS	Rv1908c	reverse	katG	Y197fs	INH-R	WHO2023	14.12.2023 VD	katG_Y197fs	2) Assoc w R - Interim	2	Tyr197fs	katG_p.Tyr197fs
2156028	G	T	SNP	CDS	Rv1908c	reverse	katG	Y28_	INH-R	WHO2023	14.12.2023 VD	katG_Y28_	2) Assoc w R - Interim	2	Tyr28*	katG_p.Tyr28*
2154509	ACAGCAGGGTC	T	INDEL	CDS	Rv1908c	reverse	katG	Y339fs	INH-R	WHO2023	14.12.2023 VD	katG_Y339fs	2) Assoc w R - Interim	2	Tyr339fs	katG_p.Tyr339fs
2154873	GTA	ATG	SNP	CDS	Rv1908c	reverse	katG	Y413_	INH-R	WHO2023	14.12.2023 VD	katG_Y413_	2) Assoc w R - Interim	2	Tyr413*	katG_p.Tyr413*
2155819	TAGTGGCCG	T	INDEL	CDS	Rv1908c	reverse	katG	Y95fs	INH-R	WHO2023	14.12.2023 VD	katG_Y95fs	2) Assoc w R - Interim	2	Tyr95fs	katG_p.Tyr95fs
2859346	TGTCCCAGTCC	G	INDEL	CDS	Rv2535c	reverse	pepQ	D232fs	BDQ-R, CFZ-R	WHO2023	14.12.2023 VD	pepQ_D232fs	2) Assoc w R - Interim	2	Asp232fs	pepQ_p.Asp232fs
2859703	GCTAGTCGCAC	A	INDEL	CDS	Rv2535c	reverse	pepQ	D51fs	BDQ-R, CFZ-R	WHO2023	14.12.2023 VD	pepQ_D51fs	2) Assoc w R - Interim	2	Asp51fs	pepQ_p.Asp51fs
2859566	CGATCGTCTCC	C	INDEL	CDS	Rv2535c	reverse	pepQ	H100fs	BDQ-R, CFZ-R	WHO2023	14.12.2023 VD	pepQ_H100fs	2) Assoc w R - Interim	2	His100fs	pepQ_p.His100fs
2859467	CCAGT	C	INDEL	CDS	Rv2535c	reverse	pepQ	L317fs	BDQ-R, CFZ-R	WHO2023	14.12.2023 VD	pepQ_L317fs	2) Assoc w R - Interim	2	Leu317fs	pepQ_p.Leu317fs
2859292	TTGTGACCCCGA	T	INDEL	CDS	Rv2535c	reverse	pepQ	LoF	BDQ-R, CFZ-R	WHO2023	14.12.2023 VD	pepQ_LoF	2) Assoc w R - Interim	2	LoF	pepQ_p.LoF
2860404	CT	GC	SNP	CDS	Rv2535c	reverse	pepQ	Q5_	BDQ-R	WHO2023	14.12.2023 VD	pepQ_Q5_	2) Assoc w R - Interim	2	Gln5*	pepQ_p.Gln5*
2859383	GCCACTACCAA	C	INDEL	CDS	Rv2535c	reverse	pepQ	T341fs	BDQ-R	WHO2023	14.12.2023 VD	pepQ_T341fs	2) Assoc w R - Interim	2	Thr341fs	pepQ_p.Thr341fs
2859601	A	AC	INDEL	CDS	Rv2535c	reverse	pepQ	V273fs	BDQ-R, CFZ-R	WHO2023	14.12.2023 VD	pepQ_V273fs	2) Assoc w R - Interim	2	Val273fs	pepQ_p.Val273fs
2289252	T	A	SNP	CDS	Rv2043c	reverse	pncA	-11A>T	PZA-R	WHO2023	14.12.2023 VD	pncA_-11A>T	2) Assoc w R - Interim	2	-11A>T	pncA_p.-11A>T
2289253	A	G	SNP	CDS	Rv2043c	reverse	pncA	-12T>C	PZA-R	WHO2023	14.12.2023 VD	pncA_-12T>C	1) Assoc w R	1	-12T>C	pncA_p.-12T>C
2289245	TC	T	INDEL	CDS	Rv2043c	reverse	pncA	-5delG	PZA-R	WHO2023	14.12.2023 VD	pncA_-5delG	1) Assoc w R	1	-5delG	pncA_p.-5delG
2289248	A	G	SNP	CDS	Rv2043c	reverse	pncA	-7T>C	PZA-R	WHO2023	14.12.2023 VD	pncA_-7T>C	2) Assoc w R - Interim	2	-7T>C	pncA_p.-7T>C
2288936	CG	C	INDEL	CDS	Rv2043c	reverse	pncA	A102fs	PZA-R	WHO2023	14.12.2023 VD	pncA_A102fs	2) Assoc w R - Interim	2	Ala102fs	pncA_p.Ala102fs
2288814	G	GC	INDEL	CDS	Rv2043c	reverse	pncA	A143fs	PZA-R	WHO2023	14.12.2023 VD	pncA_A143fs	2) Assoc w R - Interim	2	Ala143fs	pncA_p.Ala143fs
2288777	CACCCTGGTGG	C	INDEL	CDS	Rv2043c	reverse	pncA	A152fs	PZA-R	WHO2023	14.12.2023 VD	pncA_A152fs	1) Assoc w R	1	Ala152fs	pncA_p.Ala152fs
2288758	CCG	C	INDEL	CDS	Rv2043c	reverse	pncA	A161fs	PZA-R	WHO2023	14.12.2023 VD	pncA_A161fs	2) Assoc w R - Interim	2	Ala161fs	pncA_p.Ala161fs
2288748	G	GC	INDEL	CDS	Rv2043c	reverse	pncA	A165fs	PZA-R	WHO2023	14.12.2023 VD	pncA_A165fs	2) Assoc w R - Interim	2	Ala165fs	pncA_p.Ala165fs
2288703	ACGCTGG	A	INDEL	CDS	Rv2043c	reverse	pncA	A178_S179del	PZA-R	WHO2023	14.12.2023 VD	pncA_A178_S179del	2) Assoc w R - Interim	2	Ala178_Ser179del	pncA_p.Ala178_Ser179del
2288709	GC	G	INDEL	CDS	Rv2043c	reverse	pncA	A178fs	PZA-R	WHO2023	14.12.2023 VD	pncA_A178fs	2) Assoc w R - Interim	2	Ala178fs	pncA_p.Ala178fs
2288831	CGCGCCGGGAC	A	INDEL	CDS	Rv2043c	reverse	pncA	A20fs	PZA-R	WHO2023	14.12.2023 VD	pncA_A20fs	2) Assoc w R - Interim	2	Ala20fs	pncA_p.Ala20fs
2289164	CG	C	INDEL	CDS	Rv2043c	reverse	pncA	A26fs	PZA-R	WHO2023	14.12.2023 VD	pncA_A26fs	2) Assoc w R - Interim	2	Ala26fs	pncA_p.Ala26fs
2289159	G	GCCAT	INDEL	CDS	Rv2043c	reverse	pncA	A28fs	PZA-R	WHO2023	14.12.2023 VD	pncA_A28fs	2) Assoc w R - Interim	2	Ala28fs	pncA_p.Ala28fs
2289132	TCGGC	T	INDEL	CDS	Rv2043c	reverse	pncA	A36fs	PZA-R	WHO2023	14.12.2023 VD	pncA_A36fs	2) Assoc w R - Interim	2	Ala36fs	pncA_p.Ala36fs
2289109	GTGATGGTAG	C	INDEL	CDS	Rv2043c	reverse	pncA	A39fs	PZA-R	WHO2023	14.12.2023 VD	pncA_A39fs	2) Assoc w R - Interim	2	Ala39fs	pncA_p.Ala39fs
2289234	G	GC	INDEL	CDS	Rv2043c	reverse	pncA	A3fs	PZA-R	WHO2023	14.12.2023 VD	pncA_A3fs	2) Assoc w R - Interim	2	Ala3fs	pncA_p.Ala3fs
2289007	C	CG	INDEL	CDS	Rv2043c	reverse	pncA	A79fs	PZA-R	WHO2023	14.12.2023 VD	pncA_A79fs	2) Assoc w R - Interim	2	Ala79fs	pncA_p.Ala79fs
2288939	TCCGGTGTAGG	T	INDEL	CDS	Rv2043c	reverse	pncA	A98fs	PZA-R	WHO2023	14.12.2023 VD	pncA_A98fs	2) Assoc w R - Interim	2	Ala98fs	pncA_p.Ala98fs
2288850	CACATCGACCTC	A	INDEL	CDS	Rv2043c	reverse	pncA	D126_V130del	PZA-R	WHO2023	14.12.2023 VD	pncA_D126_V130del	2) Assoc w R - Interim	2	Asp126_Val130del	pncA_p.Asp126_Val130del
2288851	ACATCGACCTC	C	INDEL	CDS	Rv2043c	reverse	pncA	D126fs	PZA-R	WHO2023	14.12.2023 VD	pncA_D126fs	2) Assoc w R - Interim	2	Asp126fs	pncA_p.Asp126fs
2288847	CCGACCACAT	C	INDEL	CDS	Rv2043c	reverse	pncA	D129_V131del	PZA-R	WHO2023	14.12.2023 VD	pncA_D129_V131del	1) Assoc w R	1	Asp129_Val131del	pncA_p.Asp129_Val131del
2288846	ACCGACCACAT	A	INDEL	CDS	Rv2043c	reverse	pncA	D129fs	PZA-R	WHO2023	14.12.2023 VD	pncA_D129fs	1) Assoc w R	1	Asp129fs	pncA_p.Asp129fs
2288817	GGGAGTACCG	G	INDEL	CDS	Rv2043c	reverse	pncA	D12fs	PZA-R	WHO2023	14.12.2023 VD	pncA_D12fs	2) Assoc w R - Interim	2	Asp12fs	pncA_p.Asp12fs
2288776	TACCGCGTCCT	G	INDEL	CDS	Rv2043c	reverse	pncA	D136fs	PZA-R	WHO2023	14.12.2023 VD	pncA_D136fs	1) Assoc w R	1	Asp136fs	pncA_p.Asp136fs
2288768	G	GT	INDEL	CDS	Rv2043c	reverse	pncA	D158fs	PZA-R	WHO2023	14.12.2023 VD	pncA_D158fs	2) Assoc w R - Interim	2	Asp158fs	pncA_p.Asp158fs
2288741	GGTAT	G	INDEL	CDS	Rv2043c	reverse	pncA	D166fs	PZA-R	WHO2023	14.12.2023 VD	pncA_D166fs	2) Assoc w R - Interim	2	Asp166fs	pncA_p.Asp166fs
2289121	AGT	A	INDEL	CDS	Rv2043c	reverse	pncA	D40fs	PZA-R	WHO2023	14.12.2023 VD	pncA_D40fs	2) Assoc w R - Interim	2	Asp40fs	pncA_p.Asp40fs

2289085	GATGTGGAA	C	INDEL	CDS	Rv2043c	reverse	pncA	D49fs	PZA-R	WHO2023	14.12.2023 VD	pncA_D49fs	2) Assoc w R - Interim	2	Asp49fs	pncA_p.Asp49fs
2289071	GTGGT	G	INDEL	CDS	Rv2043c	reverse	pncA	D56fs	PZA-R	WHO2023	14.12.2023 VD	pncA_D56fs	2) Assoc w R - Interim	2	Asp56fs	pncA_p.Asp56fs
2289041	ACGAGGAAT	C	INDEL	CDS	Rv2043c	reverse	pncA	D63_S67delinsE	PZA-R	WHO2023	14.12.2023 VD	pncA_D63_S67delinsE	2) Assoc w R - Interim	2	Asp63_Ser67delins	pncA_p.Asp63_Ser67delinsGlu
2289049	ATAGTCCGG	A	INDEL	CDS	Rv2043c	reverse	pncA	D63fs	PZA-R	WHO2023	14.12.2023 VD	pncA_D63fs	1) Assoc w R	1	Asp63fs	pncA_p.Asp63fs
2289002	G	GT	INDEL	CDS	Rv2043c	reverse	pncA	D80fs	PZA-R	WHO2023	14.12.2023 VD	pncA_D80fs	2) Assoc w R - Interim	2	Asp80fs	pncA_p.Asp80fs
2288682	CAATGATCGG	C	INDEL	CDS	Rv2043c	reverse	pncA	D86fs	PZA-R	WHO2023	14.12.2023 VD	pncA_D86fs	1) Assoc w R	1	Asp86fs	pncA_p.Asp86fs
2289219	T	TC	INDEL	CDS	Rv2043c	reverse	pncA	D8fs	PZA-R	WHO2023	14.12.2023 VD	pncA_D8fs	2) Assoc w R - Interim	2	Asp8fs	pncA_p.Asp8fs
#NV	#NV	#NV	#NV	CDS	Rv2043c	reverse	pncA	deletion	PZA-R	WHO2023	14.12.2023 VD	pncA_deletion	2) Assoc w R - Interim	2	deletion	pncA_p.deletion
2288853	ACATCGACCT	A	INDEL	CDS	Rv2043c	reverse	pncA	E127_D129del	PZA-R	WHO2023	14.12.2023 VD	pncA_E127_D129del	1) Assoc w R	1	Glu127_Asp129del	pncA_p.Glu127_Asp129del
2288806	CGTCCT	C	INDEL	CDS	Rv2043c	reverse	pncA	E144fs	PZA-R	WHO2023	14.12.2023 VD	pncA_E144fs	1) Assoc w R	1	Glu144fs	pncA_p.Glu144fs
2289186	GCGAGCCACC	A	INDEL	CDS	Rv2043c	reverse	pncA	E15fs	PZA-R	WHO2023	14.12.2023 VD	pncA_E15fs	2) Assoc w R - Interim	2	Glu15fs	pncA_p.Glu15fs
2288723	CT	C	INDEL	CDS	Rv2043c	reverse	pncA	E173fs	PZA-R	WHO2023	14.12.2023 VD	pncA_E173fs	1) Assoc w R	1	Glu173fs	pncA_p.Glu173fs
2288696	CAACT	C	INDEL	CDS	Rv2043c	reverse	pncA	E181fs	PZA-R	WHO2023	14.12.2023 VD	pncA_E181fs	2) Assoc w R - Interim	2	Glu181fs	pncA_p.Glu181fs
2288776	CCCTTGTAGA	G	INDEL	CDS	Rv2043c	reverse	pncA	E37fs	PZA-R	WHO2023	14.12.2023 VD	pncA_E37fs	2) Assoc w R - Interim	2	Glu37fs	pncA_p.Glu37fs
2288923	CGA	GG	INDEL	CDS	Rv2043c	reverse	pncA	F106fs	PZA-R	WHO2023	14.12.2023 VD	pncA_F106fs	1) Assoc w R	1	Phe106fs	pncA_p.Phe106fs
2289203	GA	G	INDEL	CDS	Rv2043c	reverse	pncA	F13fs	PZA-R	WHO2023	14.12.2023 VD	pncA_F13fs	2) Assoc w R - Interim	2	Phe13fs	pncA_p.Phe13fs
2288999	GA	G	INDEL	CDS	Rv2043c	reverse	pncA	F81fs	PZA-R	WHO2023	14.12.2023 VD	pncA_F81fs	2) Assoc w R - Interim	2	Phe81fs	pncA_p.Phe81fs
2288842	TGGCGTGCCG	C	INDEL	CDS	Rv2043c	reverse	pncA	F94fs	PZA-R	WHO2023	14.12.2023 VD	pncA_F94fs	2) Assoc w R - Interim	2	Phe94fs	pncA_p.Phe94fs
2288714	GCACCCTGGT	G	INDEL	CDS	Rv2043c	reverse	pncA	G132fs	PZA-R	WHO2023	14.12.2023 VD	pncA_G132fs	2) Assoc w R - Interim	2	Gly132fs	pncA_p.Gly132fs
2288793	C	CT	INDEL	CDS	Rv2043c	reverse	pncA	G150fs	PZA-R	WHO2023	14.12.2023 VD	pncA_G150fs	2) Assoc w R - Interim	2	Gly150fs	pncA_p.Gly150fs
2288808	AAGTCCGCGC	T	INDEL	CDS	Rv2043c	reverse	pncA	G17fs	PZA-R	WHO2023	14.12.2023 VD	pncA_G17fs	2) Assoc w R - Interim	2	Gly17fs	pncA_p.Gly17fs
2288711	GGTGTAGGC	G	INDEL	CDS	Rv2043c	reverse	pncA	G23fs	PZA-R	WHO2023	14.12.2023 VD	pncA_G23fs	2) Assoc w R - Interim	2	Gly23fs	pncA_p.Gly23fs
2289170	GC	G	INDEL	CDS	Rv2043c	reverse	pncA	G24fs	PZA-R	WHO2023	14.12.2023 VD	pncA_G24fs	2) Assoc w R - Interim	2	Gly24fs	pncA_p.Gly24fs
2289076	CACC	AA	INDEL	CDS	Rv2043c	reverse	pncA	G55fs	PZA-R	WHO2023	14.12.2023 VD	pncA_G55fs	2) Assoc w R - Interim	2	Gly55fs	pncA_p.Gly55fs
2289008	GC	G	INDEL	CDS	Rv2043c	reverse	pncA	G78fs	PZA-R	WHO2023	14.12.2023 VD	pncA_G78fs	2) Assoc w R - Interim	2	Gly78fs	pncA_p.Gly78fs
2288951	AC	A	INDEL	CDS	Rv2043c	reverse	pncA	G97fs	PZA-R	WHO2023	14.12.2023 VD	pncA_G97fs	2) Assoc w R - Interim	2	Gly97fs	pncA_p.Gly97fs
2289114	TGA	T	INDEL	CDS	Rv2043c	reverse	pncA	H42fs	PZA-R	WHO2023	14.12.2023 VD	pncA_H42fs	2) Assoc w R - Interim	2	His42fs	pncA_p.His42fs
2289112	CGT	C	INDEL	CDS	Rv2043c	reverse	pncA	H43fs	PZA-R	WHO2023	14.12.2023 VD	pncA_H43fs	2) Assoc w R - Interim	2	His43fs	pncA_p.His43fs
2289010	AGGAATAGT	C	INDEL	CDS	Rv2043c	reverse	pncA	H51fs	PZA-R	WHO2023	14.12.2023 VD	pncA_H51fs	2) Assoc w R - Interim	2	His51fs	pncA_p.His51fs
2288920	GGGATGGAA	C	INDEL	CDS	Rv2043c	reverse	pncA	H57fs	PZA-R	WHO2023	14.12.2023 VD	pncA_H57fs	2) Assoc w R - Interim	2	His57fs	pncA_p.His57fs
2288682	CGCTGTCAGG	C	INDEL	CDS	Rv2043c	reverse	pncA	I133fs	PZA-R	WHO2023	14.12.2023 VD	pncA_I133fs	2) Assoc w R - Interim	2	Ile133fs	pncA_p.Ile133fs
2289225	A	AT	INDEL	CDS	Rv2043c	reverse	pncA	I6fs	PZA-R	WHO2023	14.12.2023 VD	pncA_I6fs	2) Assoc w R - Interim	2	Ile6fs	pncA_p.Ile6fs
2289036	GAGAAGTGG	G	INDEL	CDS	Rv2043c	reverse	pncA	K48fs	PZA-R	WHO2023	14.12.2023 VD	pncA_K48fs	2) Assoc w R - Interim	2	Lys48fs	pncA_p.Lys48fs
2288954	CT	C	INDEL	CDS	Rv2043c	reverse	pncA	K96fs	PZA-R	WHO2023	14.12.2023 VD	pncA_K96fs	2) Assoc w R - Interim	2	Lys96fs	pncA_p.Lys96fs
2288892	A	AG	INDEL	CDS	Rv2043c	reverse	pncA	L117fs	PZA-R	WHO2023	14.12.2023 VD	pncA_L117fs	2) Assoc w R - Interim	2	Leu117fs	pncA_p.Leu117fs
2288777	CCCTGGTGGC	C	INDEL	CDS	Rv2043c	reverse	pncA	L151fs	PZA-R	WHO2023	14.12.2023 VD	pncA_L151fs	2) Assoc w R - Interim	2	Leu151fs	pncA_p.Leu151fs
2288774	CA	C	INDEL	CDS	Rv2043c	reverse	pncA	L156fs	PZA-R	WHO2023	14.12.2023 VD	pncA_L156fs	1) Assoc w R	1	Leu156fs	pncA_p.Leu156fs
2288765	CA	C	INDEL	CDS	Rv2043c	reverse	pncA	L159fs	PZA-R	WHO2023	14.12.2023 VD	pncA_L159fs	2) Assoc w R - Interim	2	Leu159fs	pncA_p.Leu159fs
2288723	CTCCAG	C	INDEL	CDS	Rv2043c	reverse	pncA	L172fs	PZA-R	WHO2023	14.12.2023 VD	pncA_L172fs	2) Assoc w R - Interim	2	Leu172fs	pncA_p.Leu172fs
2289230	CA	C	INDEL	CDS	Rv2043c	reverse	pncA	L4fs	PZA-R	WHO2023	14.12.2023 VD	pncA_L4fs	2) Assoc w R - Interim	2	Leu4fs	pncA_p.Leu4fs
2288988	A	CTGGGATGT	INDEL	CDS	Rv2043c	reverse	pncA	L85fs	PZA-R	WHO2023	14.12.2023 VD	pncA_L85fs	2) Assoc w R - Interim	2	Leu85fs	pncA_p.Leu85fs
2288683	AGGAG	A	INDEL	CDS	Rv2043c	reverse	pncA	LoF	PZA-R	WHO2023	14.12.2023 VD	pncA_LoF	1) Assoc w R	1	LoF	pncA_p.LoF
2288689	CGCGGGCCA	T	INDEL	CDS	Rv2043c	reverse	pncA	M1?	PZA-R	WHO2023	14.12.2023 VD	pncA_M1?	1) Assoc w R	1	Met1?	pncA_p.Met1?
2288717	CA	C	INDEL	CDS	Rv2043c	reverse	pncA	M175fs	PZA-R	WHO2023	14.12.2023 VD	pncA_M175fs	2) Assoc w R - Interim	2	Met175fs	pncA_p.Met175fs
2288906	GT	G	INDEL	CDS	Rv2043c	reverse	pncA	N112fs	PZA-R	WHO2023	14.12.2023 VD	pncA_N112fs	2) Assoc w R - Interim	2	Asn112fs	pncA_p.Asn112fs
2288788	CCAAGCCAT	C	INDEL	CDS	Rv2043c	reverse	pncA	N149fs	PZA-R	WHO2023	14.12.2023 VD	pncA_N149fs	2) Assoc w R - Interim	2	Asn149fs	pncA_p.Asn149fs
2288888	TTCAGCAGT	A	INDEL	CDS	Rv2043c	reverse	pncA	P115fs	PZA-R	WHO2023	14.12.2023 VD	pncA_P115fs	2) Assoc w R - Interim	2	Pro115fs	pncA_p.Pro115fs
2288995	G	GATGGA	INDEL	CDS	Rv2043c	reverse	pncA	P83fs	PZA-R	WHO2023	14.12.2023 VD	pncA_P83fs	2) Assoc w R - Interim	2	Pro83fs	pncA_p.Pro83fs
2288877	TG	T	INDEL	CDS	Rv2043c	reverse	pncA	Q122fs	PZA-R	WHO2023	14.12.2023 VD	pncA_Q122fs	2) Assoc w R - Interim	2	Gln122fs	pncA_p.Gln122fs
2288818	TCTGGC	T	INDEL	CDS	Rv2043c	reverse	pncA	R140fs	PZA-R	WHO2023	14.12.2023 VD	pncA_R140fs	2) Assoc w R - Interim	2	Arg140fs	pncA_p.Arg140fs
2288772	ACCAGCACC	A	INDEL	CDS	Rv2043c	reverse	pncA	R154fs	PZA-R	WHO2023	14.12.2023 VD	pncA_R154fs	2) Assoc w R - Interim	2	Arg154fs	pncA_p.Arg154fs
2288682	TCCTTCGAAG	C	INDEL	CDS	Rv2043c	reverse	pncA	R29fs	PZA-R	WHO2023	14.12.2023 VD	pncA_R29fs	2) Assoc w R - Interim	2	Arg29fs	pncA_p.Arg29fs
2288918	CTTCGAAGC	T	INDEL	CDS	Rv2043c	reverse	pncA	S104_G108delinsR	PZA-R	WHO2023	14.12.2023 VD	pncA_S104_G108delinsR	2) Assoc w R - Interim	2	Ser104_Gly108deli	pncA_p.Ser104_Gly108delinsArg
2288750	CGAC	GTGGG	INDEL	CDS	Rv2043c	reverse	pncA	S164fs	PZA-R	WHO2023	14.12.2023 VD	pncA_S164fs	2) Assoc w R - Interim	2	Ser164fs	pncA_p.Ser164fs
2289180	CCGCCAGCGA	A	INDEL	CDS	Rv2043c	reverse	pncA	S18fs	PZA-R	WHO2023	14.12.2023 VD	pncA_S18fs	2) Assoc w R - Interim	2	Ser18fs	pncA_p.Ser18fs
2289140	GTAGTCGCT	G	INDEL	CDS	Rv2043c	reverse	pncA	S32fs	PZA-R	WHO2023	14.12.2023 VD	pncA_S32fs	2) Assoc w R - Interim	2	Ser32fs	pncA_p.Ser32fs
2289044	CGAGG	C	INDEL	CDS	Rv2043c	reverse	pncA	S65fs	PZA-R	WHO2023	14.12.2023 VD	pncA_S65fs	2) Assoc w R - Interim	2	Ser65fs	pncA_p.Ser65fs
2289020	GCT	G	INDEL	CDS	Rv2043c	reverse	pncA	S74fs	PZA-R	WHO2023	14.12.2023 VD	pncA_S74fs	2) Assoc w R - Interim	2	Ser74fs	pncA_p.Ser74fs
2288989	GAC	G	INDEL	CDS	Rv2043c	reverse	pncA	S84fs	PZA-R	WHO2023	14.12.2023 VD	pncA_S84fs	1) Assoc w R	1	Ser84fs	pncA_p.Ser84fs
2288900	CGT	C	INDEL	CDS	Rv2043c	reverse	pncA	T114fs	PZA-R	WHO2023	14.12.2023 VD	pncA_T114fs	2) Assoc w R - Interim	2	Thr114fs	pncA_p.Thr114fs
2288774	AGCACCCTGG	C	INDEL	CDS	Rv2043c	reverse	pncA	T153fs	PZA-R	WHO2023	14.12.2023 VD	pncA_T153fs	1) Assoc w R	1	Thr153fs	pncA_p.Thr153fs
2288712	G	GA	INDEL	CDS	Rv2043c	reverse	pncA	T177fs	PZA-R	WHO2023	14.12.2023 VD	pncA_T177fs	2) Assoc w R - Interim	2	Thr177fs	pncA_p.Thr177fs
2289172	CACCGGTT	C	INDEL	CDS	Rv2043c	reverse	pncA	T22fs	PZA-R	WHO2023	14.12.2023 VD	pncA_T22fs	2) Assoc w R - Interim	2	Thr22fs	pncA_p.Thr22fs
2287882	AACACCGTCA	C	INDEL	CDS	Rv2043c	reverse	pncA	T61fs	PZA-R	WHO2023	14.12.2023 VD	pncA_T61fs	1) Assoc w R	1	Thr61fs	pncA_p.Thr61fs
2288765	AATTCAGCAG	C	INDEL	CDS	Rv2043c	reverse	pncA	T76fs	PZA-R	WHO2023	14.12.2023 VD	pncA_T76fs	2) Assoc w R - Interim	2	Thr76fs	pncA_p.Thr76fs
2288979	GACGT	G	INDEL	CDS	Rv2043c	reverse	pncA	T87fs	PZA-R	WHO2023	14.12.2023 VD	pncA_T87fs	2) Assoc w R - Interim	2	Thr87fs	pncA_p.Thr87fs
2288680	ATCA	TTCG	SNP	CDS	Rv2043c	reverse	pncA	Ter187Rext_?	PZA-R	WHO2023	14.12.2023 VD	pncA_Ter187Rext_?	2) Assoc w R - Interim	2	Ter187Argext*?	pncA_p.Ter187Argext*?
2288681	T	C	SNP	CDS	Rv2043c	reverse	pncA	Ter187Wext_?	PZA-R	WHO2023	14.12.2023 VD	pncA_Ter187Wext_?	2) Assoc w R - Interim	2	Ter187Trpext*?	pncA_p.Ter187Trpext*?
2288852	TCGACCTCAT	C	INDEL	CDS	Rv2043c	reverse	pncA	V125fs	PZA-R	WHO2023	14.12.2023 VD	pncA_V125fs	2) Assoc w R - Interim	2	Val125fs	pncA_p.Val125fs
2288852	CACATCGA	C	INDEL	CDS	Rv2043c	reverse	pncA	V128fs	PZA-R	WHO2023	14.12.2023 VD	pncA_V128fs	1) Assoc w R	1	Val128fs	pncA_p.Val128fs
2288851	CCA	C	INDEL	CDS	Rv2043c	reverse	pncA	V130fs	PZA-R	WHO2023	14.12.2023 VD	pncA_V130fs	1) Assoc w R	1	Val130fs	pncA_p.Val130fs
2288848	CGA	C	INDEL	CDS	Rv2043c	reverse	pncA	V131fs	PZA-R	WHO2023	14.12.2023 VD	pncA_V131fs	1) Assoc w R	1	Val131fs	pncA_p.Val131fs
2288777	CA	C	INDEL	CDS	Rv2043c	reverse	pncA	V155fs	PZA-R	WHO2023	14.12.2023 VD	pncA_V155fs	2) Assoc w R - Interim	2	Val155fs	pncA_p.Val155fs
2288742	CACACCCGCT	G	INDEL	CDS	Rv2043c	reverse	pncA	V157fs	PZA-R	WHO2023	14.12.2023 VD	pncA_V157fs	2) Assoc w R - Interim	2	Val157fs	pncA_p.Val157fs
2288753	CA	C	INDEL	CDS	Rv2043c	reverse	pncA	V163fs	PZA-R	WHO2023	14.12.2023 VD	pncA_V163fs	2) Assoc w R - Interim	2	Val163fs	pncA_p.Val163fs
2288822	AAGTCCGCG	G	INDEL	CDS	Rv2043c	reverse	pncA	V21fs	PZA-R	WHO2023	14.12.2023 VD	pncA_V21fs	2) Assoc w R - Interim	2	Val21fs	pncA_p.Val21fs
2289107	CA	C	INDEL	CDS	Rv2043c	reverse	pncA	V45fs	PZA-R	WHO2023	14.12.2023 VD	pncA_V45fs	2) Assoc w R - Interim	2	Val45fs	pncA_p.Val45fs
2288874	AGGAATAGTC	C	INDEL	CDS	Rv2043c	reverse	pncA	V7fs	PZA-R	WHO2023	14.12.2023 VD	pncA_V7fs	2) Assoc w R - Interim	2	Val7fs	pncA_p.Val7fs
2288885	C	CTG	INDEL	CDS	Rv2043c	reverse	pncA	W119fs	PZA-R	WHO2023	14.12.2023 VD	pncA_W119fs	2) Assoc w R - Interim	2	Trp119fs	pncA_p.Trp119fs
2288931	CTG	C	INDEL	CDS	Rv2043c	reverse	pncA	Y103fs	PZA-R	WHO2023	14.12.2023 VD	pncA_Y103fs	2) Assoc w R - Interim	2	Tyr103fs	pncA_p.Tyr103fs
2289044	CGAGGAATA	C	INDEL	CDS	Rv2043c	reverse	pncA	Y64fs	PZA-R	WHO2023	14.12.2023 VD	pncA_Y64fs	2) Assoc w R - Interim	2	Tyr64fs	pncA_p.Tyr64fs
2288955	TTG	T	INDEL	CDS	Rv2043c	reverse	pncA	Y95fs	PZA-R	WHO2023	14.12.2023 VD	pncA_Y95fs	2) Assoc w R - Interim	2	Tyr95fs	pncA_p.Tyr95fs
2288938	TCCGGTGTA	C	INDEL	CDS	Rv2043c	reverse	pncA	Y99fs	PZA-R	WHO2023	14.12.2023 VD	pncA_Y99fs	2) Assoc w R - Interim	2	Tyr99fs	pncA_p.Tyr99fs
761107	TGGACCA	T	INDEL	PROM	Rv0667	forward	rpoB	D435_Q436del	RIF-R	WHO2023	14.12.2023 VD	rpoB_D435_Q436del	2) Assoc w R - Interim	2	Asp435_Gln436de	rpoB_c.Asp435_Gln436del
761109	GACC	G	INDEL	PROM	Rv0667	forward	rpoB	D435_Q436delinsE	RIF-R	WHO2023	14.12.2023 VD	rpoB_D435_Q436delinsE	2) Assoc w R - Interim	2	Asp435_Gln436de	rpoB_c.Asp435_Gln436delinsGlu

761108	GGAC	G	INDEL	PROM	Rv0667	forward	rpoB	D435del	RIF-R	WHO2023	14.12.2023 VD	rpoB_D435del	2) Assoc w R - Interim	2	Asp435del	rpoB_c.Asp435del
761077	TCTTCGG	T	INDEL	PROM	Rv0667	forward	rpoB	F425_G426del	RIF-R	WHO2023	14.12.2023 VD	rpoB_F425_G426del	2) Assoc w R - Interim	2	Phe425_Gly426del	rpoB_c.Phe425_Gly426del
761102	ATTCATGGAC	A	INDEL	PROM	Rv0667	forward	rpoB	F433_D435del	RIF-R	WHO2023	14.12.2023 VD	rpoB_F433_D435del	2) Assoc w R - Interim	2	Phe433_Asp435de	rpoB_c.Phe433_Asp435del
761098	CCAATTCATGG	G	INDEL	PROM	Rv0667	forward	rpoB	F433_Q436del	RIF-R	WHO2023	14.12.2023 VD	rpoB_F433_Q436del	2) Assoc w R - Interim	2	Phe433_Gln436de	rpoB_c.Phe433_Gln436del
761102	A	ATTC	INDEL	PROM	Rv0667	forward	rpoB	F433dup	RIF-R	WHO2023	14.12.2023 VD	rpoB_F433dup	1) Assoc w R	1	Phe433dup	rpoB_c.Phe433dup
761080	TCGGCAC	T	INDEL	PROM	Rv0667	forward	rpoB	G426_T427del	RIF-R	WHO2023	14.12.2023 VD	rpoB_G426_T427del	2) Assoc w R - Interim	2	Gly426_Thr427del	rpoB_c.Gly426_Thr427del
761137	CCCACAA	C	INDEL	PROM	Rv0667	forward	rpoB	H445_K446del	RIF-R	WHO2023	14.12.2023 VD	rpoB_H445_K446del	2) Assoc w R - Interim	2	His445_Lys446del	rpoB_c.His445_Lys446del
761139	CACA	C	INDEL	PROM	Rv0667	forward	rpoB	H445_K446delinsQ	RIF-R	WHO2023	14.12.2023 VD	rpoB_H445_K446delinsQ	2) Assoc w R - Interim	2	His445_Lys446deli	rpoB_c.His445_Lys446delinsGln
761087	CAGCCAGCTG	C	INDEL	PROM	Rv0667	forward	rpoB	L430_Q432del	RIF-R	WHO2023	14.12.2023 VD	rpoB_L430_Q432del	2) Assoc w R - Interim	2	Leu430_Gln432del	rpoB_c.Leu430_Gln432del
761095	T	TGAG	INDEL	PROM	Rv0667	forward	rpoB	L430_S431insR	RIF-R	WHO2023	14.12.2023 VD	rpoB_L430_S431insR	2) Assoc w R - Interim	2	Leu430_Ser431ins	rpoB_c.Leu430_Ser431insArg
761133	TTGACCCACA	CCCC	INDEL	PROM	Rv0667	forward	rpoB	L443_K446delinsPC	RIF-R	WHO2023	14.12.2023 VD	rpoB_L443_K446delinsPC	2) Assoc w R - Interim	2	Leu443_Lys446del	rpoB_c.Leu443_Lys446delinsProGln
761133	TTG	AGT	SNP	PROM	Rv0667	forward	rpoB	L443S	RIF-R	WHO2023	14.12.2023 VD	rpoB_L443S	2) Assoc w R - Interim	2	Leu443Ser	rpoB_c.Leu443Ser
761134	T	G	SNP	PROM	Rv0667	forward	rpoB	L443W	RIF-R	WHO2023	14.12.2023 VD	rpoB_L443W	2) Assoc w R - Interim	2	Leu443Trp	rpoB_c.Leu443Trp
761104	TCATGGA	T	INDEL	PROM	Rv0667	forward	rpoB	M434_D435del	RIF-R	WHO2023	14.12.2023 VD	rpoB_M434_D435del	2) Assoc w R - Interim	2	Met434_Asp435de	rpoB_c.Met434_Asp435del
761106	A	ATGG	INDEL	PROM	Rv0667	forward	rpoB	M434_D435insV	RIF-R	WHO2023	14.12.2023 VD	rpoB_M434_D435insV	2) Assoc w R - Interim	2	Met434_Asp435in	rpoB_c.Met434_Asp435insVal
761107	TGGACCAGAA	T	INDEL	PROM	Rv0667	forward	rpoB	M434_N437delinsI	RIF-R	WHO2023	14.12.2023 VD	rpoB_M434_N437delinsI	2) Assoc w R - Interim	2	Met434_Asn437de	rpoB_c.Met434_Asn437delinsIle
761106	AT	CG	SNP	PROM	Rv0667	forward	rpoB	M434R	RIF-R	WHO2023	14.12.2023 VD	rpoB_M434R	2) Assoc w R - Interim	2	Met434Arg	rpoB_c.Met434Arg
761107	TG	CA	SNP	PROM	Rv0667	forward	rpoB	M434T	RIF-R	WHO2023	14.12.2023 VD	rpoB_M434T	2) Assoc w R - Interim	2	Met434Thr	rpoB_c.Met434Thr
761114	GAACAAC	G	INDEL	PROM	Rv0667	forward	rpoB	N437_N438del	RIF-R	WHO2023	14.12.2023 VD	rpoB_N437_N438del	2) Assoc w R - Interim	2	Asn437_Asn438de	rpoB_c.Asn437_Asn438del
761114	GAAC	G	INDEL	PROM	Rv0667	forward	rpoB	N438del	RIF-R	WHO2023	14.12.2023 VD	rpoB_N438del	2) Assoc w R - Interim	2	Asn438del	rpoB_c.Asn438del
761118	AAC	CAT	SNP	PROM	Rv0667	forward	rpoB	N438H	RIF-R	WHO2023	14.12.2023 VD	rpoB_N438H	2) Assoc w R - Interim	2	Asn438His	rpoB_c.Asn438His
761121	CCG	GCC	SNP	PROM	Rv0667	forward	rpoB	P439A	RIF-R	WHO2023	14.12.2023 VD	rpoB_P439A	2) Assoc w R - Interim	2	Pro439Ala	rpoB_c.Pro439Ala
761100	CAATTCATGG	C	INDEL	PROM	Rv0667	forward	rpoB	Q432_D435delinsH	RIF-R	WHO2023	14.12.2023 VD	rpoB_Q432_D435delinsH	1) Assoc w R	1	n432_Asp435delins	rpoB_c.Gln432_Asp435delinsHis
761098	GCCAATT	G	INDEL	PROM	Rv0667	forward	rpoB	Q432_F433del	RIF-R	WHO2023	14.12.2023 VD	rpoB_Q432_F433del	2) Assoc w R - Interim	2	Gln432_Phe433de	rpoB_c.Gln432_Phe433del
761099	CCAATTCATG	C	INDEL	PROM	Rv0667	forward	rpoB	Q432_M434del	RIF-R	WHO2023	14.12.2023 VD	rpoB_Q432_M434del	2) Assoc w R - Interim	2	Gln432_Met434de	rpoB_c.Gln432_Met434del
761100	CAATTCA	C	INDEL	PROM	Rv0667	forward	rpoB	Q432_M434delinsL	RIF-R	WHO2023	14.12.2023 VD	rpoB_Q432_M434delinsL	2) Assoc w R - Interim	2	Gln432_Met434de	rpoB_c.Gln432_Met434delinsLeu
761099	CCAA	C	INDEL	PROM	Rv0667	forward	rpoB	Q432del	RIF-R	WHO2023	14.12.2023 VD	rpoB_Q432del	2) Assoc w R - Interim	2	Gln432del	rpoB_c.Gln432del
761109	GACCAGA	G	INDEL	PROM	Rv0667	forward	rpoB	Q436_N437del	RIF-R	WHO2023	14.12.2023 VD	rpoB_Q436_N437del	2) Assoc w R - Interim	2	Gln436_Asn437de	rpoB_c.Gln436_Asn437del
761111	CCAG	C	INDEL	PROM	Rv0667	forward	rpoB	Q436del	RIF-R	WHO2023	14.12.2023 VD	rpoB_Q436del	2) Assoc w R - Interim	2	Gln436del	rpoB_c.Gln436del
761112	CA	AG	SNP	PROM	Rv0667	forward	rpoB	Q436R	RIF-R	WHO2023	14.12.2023 VD	rpoB_Q436R	2) Assoc w R - Interim	2	Gln436Arg	rpoB_c.Gln436Arg
761148	CGA	TTG	SNP	PROM	Rv0667	forward	rpoB	R448L	RIF-R	WHO2023	14.12.2023 VD	rpoB_R448L	2) Assoc w R - Interim	2	Arg448Leu	rpoB_c.Arg448Leu
761094	TGAGCCAATTC	C	INDEL	PROM	Rv0667	forward	rpoB	S431_M434del	RIF-R	WHO2023	14.12.2023 VD	rpoB_S431_M434del	2) Assoc w R - Interim	2	Ser431_Met434de	rpoB_c.Ser431_Met434del
761098	G	GCCA	INDEL	PROM	Rv0667	forward	rpoB	S431_Q432insH	RIF-R	WHO2023	14.12.2023 VD	rpoB_S431_Q432insH	2) Assoc w R - Interim	2	Ser431_Gln432ins	rpoB_c.Ser431_Gln432insHis
761097	A	AGCC	INDEL	PROM	Rv0667	forward	rpoB	S431_Q432insR	RIF-R	WHO2023	14.12.2023 VD	rpoB_S431_Q432insR	2) Assoc w R - Interim	2	Ser431_Gln432ins	rpoB_c.Ser431_Gln432insArg
761098	G	A	SNP	PROM	Rv0667	forward	rpoB	S431N	RIF-R	WHO2023	14.12.2023 VD	rpoB_S431N	2) Assoc w R - Interim	2	Ser431Asn	rpoB_c.Ser431Asn
761127	TC	AA	SNP	PROM	Rv0667	forward	rpoB	S441K	RIF-R	WHO2023	14.12.2023 VD	rpoB_S441K	2) Assoc w R - Interim	2	Ser441Lys	rpoB_c.Ser441Lys
761128	C	G	SNP	PROM	Rv0667	forward	rpoB	S441W	RIF-R	WHO2023	14.12.2023 VD	rpoB_S441W	2) Assoc w R - Interim	2	Ser441Trp	rpoB_c.Ser441Trp
761150	ACTGTCGGCG	A	INDEL	PROM	Rv0667	forward	rpoB	S450_L452del	RIF-R	WHO2023	14.12.2023 VD	rpoB_S450_L452del	2) Assoc w R - Interim	2	Ser450_Leu452del	rpoB_c.Ser450_Leu452del
761083	GCACCAGCCA	G	INDEL	PROM	Rv0667	forward	rpoB	T427_Q429del	RIF-R	WHO2023	14.12.2023 VD	rpoB_T427_Q429del	2) Assoc w R - Interim	2	Thr427_Gln429del	rpoB_c.Thr427_Gln429del
761085	ACCAGCC	A	INDEL	PROM	Rv0667	forward	rpoB	T427_Q429delinsK	RIF-R	WHO2023	14.12.2023 VD	rpoB_T427_Q429delinsK	2) Assoc w R - Interim	2	Thr427_Gln429del	rpoB_c.Thr427_Gln429delinsLys
761082	GGCACCA	G	INDEL	PROM	Rv0667	forward	rpoB	T427_S428del	RIF-R	WHO2023	14.12.2023 VD	rpoB_T427_S428del	2) Assoc w R - Interim	2	Thr427_Ser428del	rpoB_c.Thr427_Ser428del
761085	ACC	CCG	SNP	PROM	Rv0667	forward	rpoB	T427P	RIF-R	WHO2023	14.12.2023 VD	rpoB_T427P	2) Assoc w R - Interim	2	Thr427Pro	rpoB_c.Thr427Pro
761134	T	TGAC	INDEL	PROM	Rv0667	forward	rpoB	T444dup	RIF-R	WHO2023	14.12.2023 VD	rpoB_T444dup	1) Assoc w R	1	Thr444dup	rpoB_c.Thr444dup
761134	TGA	GGC	SNP	PROM	Rv0667	forward	rpoB	T444P	RIF-R	WHO2023	14.12.2023 VD	rpoB_T444P	2) Assoc w R - Interim	2	Thr444Pro	rpoB_c.Thr444Pro
1475926	A	AT	INDEL	RNA	Rvnr02	forward	rrl	2269_2270insT	LZD-R	WHO2023	14.12.2023 VD	rrl_2269_2270insT	2) Assoc w R - Interim	2	2269_2270insT	rrl_n.2269_2270insT
1475927	G	T	SNP	RNA	Rvnr02	forward	rrl	2270G>T	LZD-R	WHO2023	14.12.2023 VD	rrl_2270G>T	2) Assoc w R - Interim	2	2270G>T	rrl_n.2270G>T
1476346	A	T	SNP	RNA	Rvnr02	forward	rrl	2689A>T	LZD-R	WHO2023	14.12.2023 VD	rrl_2689A>T	2) Assoc w R - Interim	2	2689A>T	rrl_n.2689A>T
779314	CG	C	INDEL	PROM	Rv0678	forward	Rv0678	A110fs	BDQ-R, CFZ-R	WHO2023	14.12.2023 VD	Rv0678_A110fs	2) Assoc w R - Interim	2	Ala110fs	Rv0678_c.Ala110fs
779417	TG	T	INDEL	PROM	Rv0678	forward	Rv0678	A144fs	BDQ-R, CFZ-R	WHO2023	14.12.2023 VD	Rv0678_A144fs	2) Assoc w R - Interim	2	Ala144fs	Rv0678_c.Ala144fs
779171	C	CG	INDEL	PROM	Rv0678	forward	Rv0678	A62fs	BDQ-R, CFZ-R	WHO2023	14.12.2023 VD	Rv0678_A62fs	2) Assoc w R - Interim	2	Ala62fs	Rv0678_c.Ala62fs
779120	TGG	T	INDEL	PROM	Rv0678	forward	Rv0678	C46fs	BDQ-R, CFZ-R	WHO2023	14.12.2023 VD	Rv0678_C46fs	2) Assoc w R - Interim	2	Cys46fs	Rv0678_c.Cys46fs
779333	AGGACCTG	A	INDEL	PROM	Rv0678	forward	Rv0678	D116fs	BDQ-R, CFZ-R	WHO2023	14.12.2023 VD	Rv0678_D116fs	2) Assoc w R - Interim	2	Asp116fs	Rv0678_c.Asp116fs
779407	CG	C	INDEL	PROM	Rv0678	forward	Rv0678	D141fs	BDQ-R, CFZ-R	WHO2023	14.12.2023 VD	Rv0678_D141fs	2) Assoc w R - Interim	2	Asp141fs	Rv0678_c.Asp141fs
779479	G	GAC	INDEL	PROM	Rv0678	forward	Rv0678	D165fs	BDQ-R, CFZ-R	WHO2023	14.12.2023 VD	Rv0678_D165fs	2) Assoc w R - Interim	2	Asp165fs	Rv0678_c.Asp165fs
779121	G	GGT	INDEL	PROM	Rv0678	forward	Rv0678	D47fs	BDQ-R, CFZ-R	WHO2023	14.12.2023 VD	Rv0678_D47fs	1) Assoc w R	1	Asp47fs	Rv0678_c.Asp47fs
779003	A	ACGGGGTG	INDEL	PROM	Rv0678	forward	Rv0678	D8fs	BDQ-R, CFZ-R	WHO2023	14.12.2023 VD	Rv0678_D8fs	2) Assoc w R - Interim	2	Asp8fs	Rv0678_c.Asp8fs
779401	GAG	TAA	SNP	PROM	Rv0678	forward	Rv0678	E138_	BDQ-R, CFZ-R	WHO2023	14.12.2023 VD	Rv0678_E138_	2) Assoc w R - Interim	2	Glu138*	Rv0678_c.Glu138*
779428	GAG	TAA	SNP	PROM	Rv0678	forward	Rv0678	E147_	BDQ-R	WHO2023	14.12.2023 VD	Rv0678_E147_	2) Assoc w R - Interim	2	Glu147*	Rv0678_c.Glu147*
779130	TC	T	INDEL	PROM	Rv0678	forward	Rv0678	E49fs	BDQ-R, CFZ-R	WHO2023	14.12.2023 VD	Rv0678_E49fs	1) Assoc w R	1	Glu49fs	Rv0678_c.Glu49fs
779150	A	AG	INDEL	PROM	Rv0678	forward	Rv0678	E55fs	BDQ-R	WHO2023	14.12.2023 VD	Rv0678_E55fs	2) Assoc w R - Interim	2	Glu55fs	Rv0678_c.Glu55fs
779018	TG	T	INDEL	PROM	Rv0678	forward	Rv0678	G11fs	BDQ-R, CFZ-R	WHO2023	14.12.2023 VD	Rv0678_G11fs	2) Assoc w R - Interim	2	Gly11fs	Rv0678_c.Gly11fs
779108	TG	T	INDEL	PROM	Rv0678	forward	Rv0678	G41fs	BDQ-R, CFZ-R	WHO2023	14.12.2023 VD	Rv0678_G41fs	2) Assoc w R - Interim	2	Gly41fs	Rv0678_c.Gly41fs
779034	C	CA	INDEL	PROM	Rv0678	forward	Rv0678	I16fs	BDQ-R, CFZ-R	WHO2023	14.12.2023 VD	Rv0678_I16fs	2) Assoc w R - Interim	2	Ile16fs	Rv0678_c.Ile16fs
779181	C	CG	INDEL	PROM	Rv0678	forward	Rv0678	I67fs	BDQ-R, CFZ-R	WHO2023	14.12.2023 VD	Rv0678_I67fs	1) Assoc w R	1	Ile67fs	Rv0678_c.Ile67fs
779223	G	GT	INDEL	PROM	Rv0678	forward	Rv0678	I80fs	BDQ-R, CFZ-R	WHO2023	14.12.2023 VD	Rv0678_I80fs	2) Assoc w R - Interim	2	Ile80fs	Rv0678_c.Ile80fs
779336	A	ACC	INDEL	PROM	Rv0678	forward	Rv0678	L117fs	BDQ-R	WHO2023	14.12.2023 VD	Rv0678_L117fs	2) Assoc w R - Interim	2	Leu117fs	Rv0678_c.Leu117fs
779358	G	GGC	INDEL	PROM	Rv0678	forward	Rv0678	L125fs	BDQ-R, CFZ-R	WHO2023	14.12.2023 VD	Rv0678_L125fs	2) Assoc w R - Interim	2	Leu125fs	Rv0678_c.Leu125fs
779411	AT	A	INDEL	PROM	Rv0678	forward	Rv0678	L142fs	BDQ-R, CFZ-R	WHO2023	14.12.2023 VD	Rv0678_L142fs	2) Assoc w R - Interim	2	Leu142fs	Rv0678_c.Leu142fs
779411	A	ATC	INDEL	PROM	Rv0678	forward	Rv0678	L143fs	BDQ-R	WHO2023	14.12.2023 VD	Rv0678_L143fs	2) Assoc w R - Interim	2	Leu143fs	Rv0678_c.Leu143fs
779081	GT	G	INDEL	PROM	Rv0678	forward	Rv0678	L32fs	BDQ-R, CFZ-R	WHO2023	14.12.2023 VD	Rv0678_L32fs	2) Assoc w R - Interim	2	Leu32fs	Rv0678_c.Leu32fs
779083	TTG	AGC	SNP	PROM	Rv0678	forward	Rv0678	L32S	BDQ-R, CFZ-R	WHO2023	14.12.2023 VD	Rv0678_L32S	2) Assoc w R - Interim	2	Leu32Ser	Rv0678_c.Leu32Ser
779113	T	TG	INDEL	PROM	Rv0678	forward	Rv0678	L43fs	BDQ-R	WHO2023	14.12.2023 VD	Rv0678_L43fs	2) Assoc w R - Interim	2	Leu43fs	Rv0678_c.Leu43fs
779116	CTGCTGGTGTG	C	INDEL	PROM	Rv0678	forward	Rv0678	L44fs	BDQ-R, CFZ-R	WHO2023	14.12.2023 VD	Rv0678_L44fs	2) Assoc w R - Interim	2	Leu44fs	Rv0678_c.Leu44fs
779154	GAACGCATCC	A	INDEL	PROM	Rv0678	forward	Rv0678	L56fs	BDQ-R, CFZ-R	WHO2023	14.12.2023 VD	Rv0678_L56fs	2) Assoc w R - Interim	2	Leu56fs	Rv0678_c.Leu56fs
779231	A	AGC	INDEL	PROM	Rv0678	forward	Rv0678	L83fs	BDQ-R	WHO2023	14.12.2023 VD	Rv0678_L83fs	2) Assoc w R - Interim	2	Leu83fs	Rv0678_c.Leu83fs
778993	AGC	TAG	SNP	PROM	Rv0678	forward	Rv0678	LoF	BDQ-R, CFZ-R	WHO2023	14.12.2023 VD	Rv0678_LoF	1) Assoc w R	1	LoF	Rv0678_c.LoF
778990	G	GT	INDEL	PROM	Rv0678	forward	Rv0678	M1?	BDQ-R, CFZ-R	WHO2023	14.12.2023 VD	Rv0678_M1?	2) Assoc w R - Interim	2	Met1?	Rv0678_c.Met1?
779014	C	CAG	INDEL	PROM	Rv0678	forward	Rv0678	M10fs	BDQ-R, CFZ-R	WHO2023	14.12.2023 VD	Rv0678_M10fs	2) Assoc w R - Interim	2	Met10fs	Rv0678_c.Met10fs
779317	G	GC	INDEL	PROM	Rv0678	forward	Rv0678	M111fs	BDQ-R, CFZ-R	WHO2023	14.12.2023 VD	Rv0678_M111fs	2) Assoc w R - Interim	2	Met111fs	Rv0678_c.Met111fs
779414	T	TTTGGCATAT	INDEL	PROM	Rv0678	forward	Rv0678	M146fs	BDQ-R	WHO2023	14.12.2023 VD	Rv0678_M146fs	2) Assoc w R - Interim	2	Met146fs	Rv0678_c.Met146fs
779430	G	GT	INDEL	PROM	Rv0678	forward	Rv0678	N148fs	BDQ-R	WHO2023	14.12.2023 VD	Rv0678_N148fs	2) Assoc w R - Interim	2	Asn148fs	Rv0678_c.Asn148fs
779194	A	AC	INDEL	PROM	Rv0678	forward	Rv0678	N70fs	BDQ-R, CFZ-R	WHO2023	14.12.2023 VD	Rv0678_N70fs	2) Assoc w R - Interim	2	Asn70fs	Rv0678_c.Asn70fs
779275	CG	C	INDEL	PROM	Rv0678	forward	Rv0678	N98fs	BDQ-R, CFZ-R	WHO2023	14.12.2023 VD	Rv0678_N98fs	2) Assoc w R - Interim	2	Asn98fs	Rv0678_c.Asn98fs
779370	GCCCCGCCGC	C	INDEL	PROM	Rv0678	forward	Rv0678	P129fs	BDQ-R, CFZ-R	WHO2023	14.12.2023 VD	Rv0678_P129fs	2) Assoc w R - Interim	2	Pro129fs	Rv0678_c.Pro129fs
779127	T	TGATC	INDEL	PROM	Rv0678	forward	Rv0678	P48fs	BDQ-R, CFZ-R	WHO2023	14.12.2023 VD	Rv0678_P48fs	2) Assoc w R - Interim	2	Pro48fs	Rv0678_c.Pro48fs

779332	C	T	SNP	PROM	Rv0678	forward	Rv0678	Q115_	BDQ-R, CFZ-R	WHO2023	14.12.2023 VD	Rv0678_Q115_	2) Assoc w R - Interim	2	Gln115*	Rv0678_c.Gln115*
779459	ACAGC	A	INDEL	PROM	Rv0678	forward	Rv0678	Q159fs	BDQ-R	WHO2023	14.12.2023 VD	Rv0678_Q159fs	2) Assoc w R - Interim	2	Gln159fs	Rv0678_c.Gln159fs
779137	C	CG	INDEL	PROM	Rv0678	forward	Rv0678	Q51fs	BDQ-R	WHO2023	14.12.2023 VD	Rv0678_Q51fs	2) Assoc w R - Interim	2	Gln51fs	Rv0678_c.Gln51fs
779215	CAA	AAG	SNP	PROM	Rv0678	forward	Rv0678	Q76_	BDQ-R, CFZ-R	WHO2023	14.12.2023 VD	Rv0678_Q76_	2) Assoc w R - Interim	2	Gln76*	Rv0678_c.Gln76*
779313	CCGGG	C	INDEL	PROM	Rv0678	forward	Rv0678	R109fs	BDQ-R, CFZ-R	WHO2023	14.12.2023 VD	Rv0678_R109fs	2) Assoc w R - Interim	2	Arg109fs	Rv0678_c.Arg109fs
779353	C	CT	INDEL	PROM	Rv0678	forward	Rv0678	R123fs	BDQ-R	WHO2023	14.12.2023 VD	Rv0678_R123fs	2) Assoc w R - Interim	2	Arg123fs	Rv0678_c.Arg123fs
779381	A	AG	INDEL	PROM	Rv0678	forward	Rv0678	R132fs	BDQ-R, CFZ-R	WHO2023	14.12.2023 VD	Rv0678_R132fs	2) Assoc w R - Interim	2	Arg132fs	Rv0678_c.Arg132fs
779389	C	T	SNP	PROM	Rv0678	forward	Rv0678	R134_	BDQ-R	WHO2023	14.12.2023 VD	Rv0678_R134_	2) Assoc w R - Interim	2	Arg134*	Rv0678_c.Arg134*
779450	T	TG	INDEL	PROM	Rv0678	forward	Rv0678	R156_	BDQ-R, CFZ-R	WHO2023	14.12.2023 VD	Rv0678_R156_	2) Assoc w R - Interim	2	Arg156*	Rv0678_c.Arg156*
779450	T	TG	INDEL	PROM	Rv0678	forward	Rv0678	R156fs	BDQ-R, CFZ-R	WHO2023	14.12.2023 VD	Rv0678_R156fs	2) Assoc w R - Interim	2	Arg156fs	Rv0678_c.Arg156fs
779067	T	TTTCGAGTCCA	INDEL	PROM	Rv0678	forward	Rv0678	R30fs	BDQ-R, CFZ-R	WHO2023	14.12.2023 VD	Rv0678_R30fs	2) Assoc w R - Interim	2	Arg30fs	Rv0678_c.Arg30fs
779199	T	TGC	INDEL	PROM	Rv0678	forward	Rv0678	R72fs	BDQ-R, CFZ-R	WHO2023	14.12.2023 VD	Rv0678_R72fs	2) Assoc w R - Interim	2	Arg72fs	Rv0678_c.Arg72fs
779254	C	CT	INDEL	PROM	Rv0678	forward	Rv0678	R89fs	BDQ-R	WHO2023	14.12.2023 VD	Rv0678_R89fs	2) Assoc w R - Interim	2	Arg89fs	Rv0678_c.Arg89fs
779189	T	TCA	INDEL	PROM	Rv0678	forward	Rv0678	S68fs	BDQ-R, CFZ-R	WHO2023	14.12.2023 VD	Rv0678_S68fs	2) Assoc w R - Interim	2	Ser68fs	Rv0678_c.Ser68fs
779188	A	ATC	INDEL	PROM	Rv0678	forward	Rv0678	T69fs	BDQ-R	WHO2023	14.12.2023 VD	Rv0678_T69fs	2) Assoc w R - Interim	2	Thr69fs	Rv0678_c.Thr69fs
779004	CG	C	INDEL	PROM	Rv0678	forward	Rv0678	V7fs	BDQ-R, CFZ-R	WHO2023	14.12.2023 VD	Rv0678_V7fs	2) Assoc w R - Interim	2	Val7fs	Rv0678_c.Val7fs
779110	iCCCAACGCTT	G	INDEL	PROM	Rv0678	forward	Rv0678	W42fs	BDQ-R	WHO2023	14.12.2023 VD	Rv0678_W42fs	2) Assoc w R - Interim	2	Trp42fs	Rv0678_c.Trp42fs
779413	C	CTGTTGGCA	INDEL	PROM	Rv0678	forward	Rv0678	Y145_	BDQ-R, CFZ-R	WHO2023	14.12.2023 VD	Rv0678_Y145_	2) Assoc w R - Interim	2	Tyr145*	Rv0678_c.Tyr145*
779413	C	CTGTTGGCA	INDEL	PROM	Rv0678	forward	Rv0678	Y145fs	BDQ-R	WHO2023	14.12.2023 VD	Rv0678_Y145fs	2) Assoc w R - Interim	2	Tyr145fs	Rv0678_c.Tyr145fs
779453	G	GGC	INDEL	PROM	Rv0678	forward	Rv0678	Y157fs	BDQ-R, CFZ-R	WHO2023	14.12.2023 VD	Rv0678_Y157fs	2) Assoc w R - Interim	2	Tyr157fs	Rv0678_c.Tyr157fs
779261	C	TCTCTGATCTG	INDEL	PROM	Rv0678	forward	Rv0678	Y92_	BDQ-R	WHO2023	14.12.2023 VD	Rv0678_Y92_	2) Assoc w R - Interim	2	Tyr92*	Rv0678_c.Tyr92*
779261	C	TCTCTGATCTG	INDEL	PROM	Rv0678	forward	Rv0678	Y92fs	BDQ-R, CFZ-R	WHO2023	14.12.2023 VD	Rv0678_Y92fs	2) Assoc w R - Interim	2	Tyr92fs	Rv0678_c.Tyr92fs
3339001	iCTGCGGCGGC	C	INDEL	#NV	#NV	#NV	Rv2983	LoF	DLM-R	WHO2023	14.12.2023 VD	Rv2983_LoF	2) Assoc w R - Interim	2	LoF	#NV
3339687	CCCCCGCCGAC	C	INDEL	#NV	#NV	#NV	Rv2983	T191fs	DLM-R	WHO2023	14.12.2023 VD	Rv2983_T191fs	2) Assoc w R - Interim	2	Thr191fs	#NV
1918248	T	TG	INDEL	CDS	Rv1694	forward	tlyA	A105fs	CAP-R	WHO2023	14.12.2023 VD	tlyA_A105fs	2) Assoc w R - Interim	2	Ala105fs	tlyA_p.Ala105fs
1918252	GC	G	INDEL	CDS	Rv1694	forward	tlyA	A106fs	CAP-R	WHO2023	14.12.2023 VD	tlyA_A106fs	2) Assoc w R - Interim	2	Ala106fs	tlyA_p.Ala106fs
1918263	G	GGT	INDEL	CDS	Rv1694	forward	tlyA	A110fs	CAP-R	WHO2023	14.12.2023 VD	tlyA_A110fs	2) Assoc w R - Interim	2	Ala110fs	tlyA_p.Ala110fs
1918267	G	GC	INDEL	CDS	Rv1694	forward	tlyA	A111fs	CAP-R	WHO2023	14.12.2023 VD	tlyA_A111fs	2) Assoc w R - Interim	2	Ala111fs	tlyA_p.Ala111fs
1918551	T	TG	INDEL	CDS	Rv1694	forward	tlyA	A205fs	CAP-R	WHO2023	14.12.2023 VD	tlyA_A205fs	2) Assoc w R - Interim	2	Ala205fs	tlyA_p.Ala205fs
1918577	G	GGC	INDEL	CDS	Rv1694	forward	tlyA	A215fs	CAP-R	WHO2023	14.12.2023 VD	tlyA_A215fs	2) Assoc w R - Interim	2	Ala215fs	tlyA_p.Ala215fs
1918062	G	GC	INDEL	CDS	Rv1694	forward	tlyA	A43fs	CAP-R	WHO2023	14.12.2023 VD	tlyA_A43fs	2) Assoc w R - Interim	2	Ala43fs	tlyA_p.Ala43fs
1918163	AG	A	INDEL	CDS	Rv1694	forward	tlyA	A76fs	CAP-R	WHO2023	14.12.2023 VD	tlyA_A76fs	2) Assoc w R - Interim	2	Ala76fs	tlyA_p.Ala76fs
1918192	CTCACACCGGA	C	INDEL	CDS	Rv1694	forward	tlyA	C86fs	CAP-R	WHO2023	14.12.2023 VD	tlyA_C86fs	2) Assoc w R - Interim	2	Cys86fs	tlyA_p.Cys86fs
1918012	GAG	TAA	SNP	CDS	Rv1694	forward	tlyA	E25_	CAP-R	WHO2023	14.12.2023 VD	tlyA_E25_	2) Assoc w R - Interim	2	Glu25*	tlyA_p.Glu25*
1918164	G	GGC	INDEL	CDS	Rv1694	forward	tlyA	F77fs	CAP-R	WHO2023	14.12.2023 VD	tlyA_F77fs	2) Assoc w R - Interim	2	Phe77fs	tlyA_p.Phe77fs
1918342	A	AACGC	INDEL	CDS	Rv1694	forward	tlyA	G138fs	CAP-R	WHO2023	14.12.2023 VD	tlyA_G138fs	2) Assoc w R - Interim	2	Gly138fs	tlyA_p.Gly138fs
1918034	CGCGCCTGGG	T	INDEL	CDS	Rv1694	forward	tlyA	G36fs	CAP-R	WHO2023	14.12.2023 VD	tlyA_G36fs	2) Assoc w R - Interim	2	Gly36fs	tlyA_p.Gly36fs
1918129	TCG	T	INDEL	CDS	Rv1694	forward	tlyA	G66_	CAP-R	WHO2023	14.12.2023 VD	tlyA_G66_	2) Assoc w R - Interim	2	Gly66*	tlyA_p.Gly66*
1918129	TCG	T	INDEL	CDS	Rv1694	forward	tlyA	G66fs	CAP-R	WHO2023	14.12.2023 VD	tlyA_G66fs	2) Assoc w R - Interim	2	Gly66fs	tlyA_p.Gly66fs
1918527	G	GGTGGT	INDEL	CDS	Rv1694	forward	tlyA	H199fs	CAP-R	WHO2023	14.12.2023 VD	tlyA_H199fs	2) Assoc w R - Interim	2	His199fs	tlyA_p.His199fs
1918135	GGA	G	INDEL	CDS	Rv1694	forward	tlyA	H68fs	CAP-R	WHO2023	14.12.2023 VD	tlyA_H68fs	2) Assoc w R - Interim	2	His68fs	tlyA_p.His68fs
1918367	CG	C	INDEL	CDS	Rv1694	forward	tlyA	I144fs	CAP-R	WHO2023	14.12.2023 VD	tlyA_I144fs	2) Assoc w R - Interim	2	Ile144fs	tlyA_p.Ile144fs
1918015	T	TTG	INDEL	CDS	Rv1694	forward	tlyA	I27fs	CAP-R	WHO2023	14.12.2023 VD	tlyA_I27fs	2) Assoc w R - Interim	2	Ile27fs	tlyA_p.Ile27fs
1918034	T	TGC	INDEL	CDS	Rv1694	forward	tlyA	I34fs	CAP-R	WHO2023	14.12.2023 VD	tlyA_I34fs	2) Assoc w R - Interim	2	Ile34fs	tlyA_p.Ile34fs
1918499	T	TG	INDEL	CDS	Rv1694	forward	tlyA	K189fs	CAP-R	WHO2023	14.12.2023 VD	tlyA_K189fs	2) Assoc w R - Interim	2	Lys189fs	tlyA_p.Lys189fs
1918612	G	GTC	INDEL	CDS	Rv1694	forward	tlyA	K226fs	CAP-R	WHO2023	14.12.2023 VD	tlyA_K226fs	2) Assoc w R - Interim	2	Lys226fs	tlyA_p.Lys226fs
1918026	C	CG	INDEL	CDS	Rv1694	forward	tlyA	K31fs	CAP-R	WHO2023	14.12.2023 VD	tlyA_K31fs	2) Assoc w R - Interim	2	Lys31fs	tlyA_p.Lys31fs
1918325	T	TGGTCC	INDEL	CDS	Rv1694	forward	tlyA	L131fs	CAP-R	WHO2023	14.12.2023 VD	tlyA_L131fs	2) Assoc w R - Interim	2	Leu131fs	tlyA_p.Leu131fs
1918350	T	TG	INDEL	CDS	Rv1694	forward	tlyA	L139fs	CAP-R	WHO2023	14.12.2023 VD	tlyA_L139fs	2) Assoc w R - Interim	2	Leu139fs	tlyA_p.Leu139fs
1918563	G	GC	INDEL	CDS	Rv1694	forward	tlyA	L209fs	CAP-R	WHO2023	14.12.2023 VD	tlyA_L209fs	2) Assoc w R - Interim	2	Leu209fs	tlyA_p.Leu209fs
1918157	C	CG	INDEL	CDS	Rv1694	forward	tlyA	L74fs	CAP-R	WHO2023	14.12.2023 VD	tlyA_L74fs	2) Assoc w R - Interim	2	Leu74fs	tlyA_p.Leu74fs
1917943	GC	TA	SNP	CDS	Rv1694	forward	tlyA	LoF	CAP-R	WHO2023	14.12.2023 VD	tlyA_LoF	1) Assoc w R	1	LoF	tlyA_p.LoF
1917933	GCCGGCGCTG	G	INDEL	CDS	Rv1694	forward	tlyA	M1?	CAP-R	WHO2023	14.12.2023 VD	tlyA_M1?	2) Assoc w R - Interim	2	Met1?	tlyA_p.Met1?
1918310	A	ACGAT	INDEL	CDS	Rv1694	forward	tlyA	P126fs	CAP-R	WHO2023	14.12.2023 VD	tlyA_P126fs	2) Assoc w R - Interim	2	Pro126fs	tlyA_p.Pro126fs
1918634	GC	G	INDEL	CDS	Rv1694	forward	tlyA	P233fs	CAP-R	WHO2023	14.12.2023 VD	tlyA_P233fs	2) Assoc w R - Interim	2	Pro233fs	tlyA_p.Pro233fs
1918288	CAG	TAA	SNP	CDS	Rv1694	forward	tlyA	Q117_	CAP-R	WHO2023	14.12.2023 VD	tlyA_Q117_	2) Assoc w R - Interim	2	Gln117*	tlyA_p.Gln117*
1918488	GC	G	INDEL	CDS	Rv1694	forward	tlyA	Q184_	CAP-R	WHO2023	14.12.2023 VD	tlyA_Q184_	2) Assoc w R - Interim	2	Gln184*	tlyA_p.Gln184*
1918488	GC	G	INDEL	CDS	Rv1694	forward	tlyA	Q184fs	CAP-R	WHO2023	14.12.2023 VD	tlyA_Q184fs	2) Assoc w R - Interim	2	Gln184fs	tlyA_p.Gln184fs
1918508	G	GTC	INDEL	CDS	Rv1694	forward	tlyA	Q191fs	CAP-R	WHO2023	14.12.2023 VD	tlyA_Q191fs	2) Assoc w R - Interim	2	Gln191fs	tlyA_p.Gln191fs
1918000	CAA	TAG	SNP	CDS	Rv1694	forward	tlyA	Q21_	CAP-R	WHO2023	14.12.2023 VD	tlyA_Q21_	2) Assoc w R - Interim	2	Gln21*	tlyA_p.Gln21*
1918003	CAG	TGA	SNP	CDS	Rv1694	forward	tlyA	Q22_	CAP-R	WHO2023	14.12.2023 VD	tlyA_Q22_	2) Assoc w R - Interim	2	Gln22*	tlyA_p.Gln22*
1918332	C	CGA	INDEL	CDS	Rv1694	forward	tlyA	R133fs	CAP-R	WHO2023	14.12.2023 VD	tlyA_R133fs	1) Assoc w R	1	Arg133fs	tlyA_p.Arg133fs
1917991	CGA	TAG	SNP	CDS	Rv1694	forward	tlyA	R18_	CAP-R	WHO2023	14.12.2023 VD	tlyA_R18_	2) Assoc w R - Interim	2	Arg18*	tlyA_p.Arg18*
1918571	TCG	T	INDEL	CDS	Rv1694	forward	tlyA	R213fs	CAP-R	WHO2023	14.12.2023 VD	tlyA_R213fs	2) Assoc w R - Interim	2	Arg213fs	tlyA_p.Arg213fs
1917946	C	T	SNP	CDS	Rv1694	forward	tlyA	R3_	CAP-R	WHO2023	14.12.2023 VD	tlyA_R3_	2) Assoc w R - Interim	2	Arg3*	tlyA_p.Arg3*
1918113	T	TGA	INDEL	CDS	Rv1694	forward	tlyA	R60fs	CAP-R	WHO2023	14.12.2023 VD	tlyA_R60fs	2) Assoc w R - Interim	2	Arg60fs	tlyA_p.Arg60fs
1917944	ACGTGCCCGC	C	INDEL	CDS	Rv1694	forward	tlyA	R6fs	CAP-R	WHO2023	14.12.2023 VD	tlyA_R6fs	2) Assoc w R - Interim	2	Arg6fs	tlyA_p.Arg6fs
1918403	TGTC	T	INDEL	CDS	Rv1694	forward	tlyA	S156_	CAP-R	WHO2023	14.12.2023 VD	tlyA_S156_	2) Assoc w R - Interim	2	Ser156*	tlyA_p.Ser156*
1918415	CG	GA	SNP	CDS	Rv1694	forward	tlyA	S159_	CAP-R	WHO2023	14.12.2023 VD	tlyA_S159_	2) Assoc w R - Interim	2	Ser159*	tlyA_p.Ser159*
1918450	G	GC	INDEL	CDS	Rv1694	forward	tlyA	S172fs	CAP-R	WHO2023	14.12.2023 VD	tlyA_S172fs	2) Assoc w R - Interim	2	Ser172fs	tlyA_p.Ser172fs
1918551	T	TGC	INDEL	CDS	Rv1694	forward	tlyA	S207fs	CAP-R	WHO2023	14.12.2023 VD	tlyA_S207fs	2) Assoc w R - Interim	2	Ser207fs	tlyA_p.Ser207fs
1918617	GGCCA	G	INDEL	CDS	Rv1694	forward	tlyA	S228fs	CAP-R	WHO2023	14.12.2023 VD	tlyA_S228fs	2) Assoc w R - Interim	2	Ser228fs	tlyA_p.Ser228fs
1918629	G	GC	INDEL	CDS	Rv1694	forward	tlyA	S234fs	CAP-R	WHO2023	14.12.2023 VD	tlyA_S234fs	2) Assoc w R - Interim	2	Ser234fs	tlyA_p.Ser234fs
1918690	T	TTG	INDEL	CDS	Rv1694	forward	tlyA	S252fs	CAP-R	WHO2023	14.12.2023 VD	tlyA_S252fs	2) Assoc w R - Interim	2	Ser252fs	tlyA_p.Ser252fs
1918205	C	CGG	INDEL	CDS	Rv1694	forward	tlyA	S92fs	CAP-R	WHO2023	14.12.2023 VD	tlyA_S92fs	2) Assoc w R - Interim	2	Ser92fs	tlyA_p.Ser92fs
1918331	T	TCGAG	INDEL	CDS	Rv1694	forward	tlyA	T134fs	CAP-R	WHO2023	14.12.2023 VD	tlyA_T134fs	2) Assoc w R - Interim	2	Thr134fs	tlyA_p.Thr134fs
1918421	CT	C	INDEL	CDS	Rv1694	forward	tlyA	T162fs	CAP-R	WHO2023	14.12.2023 VD	tlyA_T162fs	2) Assoc w R - Interim	2	Thr162fs	tlyA_p.Thr162fs
1918667	T	TGC	INDEL	CDS	Rv1694	forward	tlyA	T245fs	CAP-R	WHO2023	14.12.2023 VD	tlyA_T245fs	2) Assoc w R - Interim	2	Thr245fs	tlyA_p.Thr245fs
1918081	G	GAC	INDEL	CDS	Rv1694	forward	tlyA	T50fs	CAP-R	WHO2023	14.12.2023 VD	tlyA_T50fs	2) Assoc w R - Interim	2	Thr50fs	tlyA_p.Thr50fs
1918323	G	GGT	INDEL	CDS	Rv1694	forward	tlyA	V130fs	CAP-R	WHO2023	14.12.2023 VD	tlyA_V130fs	2) Assoc w R - Interim	2	Val130fs	tlyA_p.Val130fs
1918480	GT	G	INDEL	CDS	Rv1694	forward	tlyA	V181fs	CAP-R	WHO2023	14.12.2023 VD	tlyA_V181fs	2) Assoc w R - Interim	2	Val181fs	tlyA_p.Val181fs
1918524	T	TGG	INDEL	CDS	Rv1694	forward	tlyA	V197fs	CAP-R	WHO2023	14.12.2023 VD	tlyA_V197fs	2) Assoc w R - Interim	2	Val197fs	tlyA_p.Val197fs
1918521	C	CGGTGG	INDEL	CDS	Rv1694	forward	tlyA	V198fs	CAP-R	WHO2023	14.12.2023 VD	tlyA_V198fs	2) Assoc w R - Interim	2	Val198fs	tlyA_p.Val198fs
1918603	A	AGC	INDEL	CDS	Rv1694	forward	tlyA	V223fs	CAP-R	WHO2023	14.12.2023 VD	tlyA_V223fs	2) Assoc w R - Interim	2	Val223fs	tlyA_p.Val223fs
1918074	C	CG	INDEL	CDS	Rv1694	forward	tlyA	V46fs	CAP-R	WHO2023	14.12.2023 VD	tlyA_V46fs	2) Assoc w R - Interim	2	Val46fs	tlyA_p.Val46fs

1918096	A	AC	INDEL	CDS	Rv1694	forward	tlyA	V54fs	CAP-R	WHO2023	14.12.2023	VD	tlyA_V54fs	2) Assoc w R - Interim	2	Val54fs	tlyA_p.Val54fs
1918592	T	TC	INDEL	CDS	Rv1694	forward	tlyA	W220fs	CAP-R	WHO2023	14.12.2023	VD	tlyA_W220fs	2) Assoc w R - Interim	2	Trp220fs	tlyA_p.Trp220fs
1918122	C	CT	INDEL	CDS	Rv1694	forward	tlyA	W62fs	CAP-R	WHO2023	14.12.2023	VD	tlyA_W62fs	2) Assoc w R - Interim	2	Trp62fs	tlyA_p.Trp62fs
1918647	T	TGTCGA	INDEL	CDS	Rv1694	forward	tlyA	Y239fs	CAP-R	WHO2023	14.12.2023	VD	tlyA_Y239fs	2) Assoc w R - Interim	2	Tyr239fs	tlyA_p.Tyr239fs
