# Supplementary material for: Clinical epidemiology, genetic diversity, and drug susceptibility patterns by whole genome sequencing of Mycobacterium tuberculosis complex isolates in Gabon from 2012 to 2022
Source: IJID Reg. 2024 Nov 28;14:100501. doi: 10.1016/j.ijregi.2024.100501 (PMC11718294; doi:10.1016/j.ijregi.2024.100501)
Supplement: Supplementary file 2 — Supplementary Table S2. Cluster by lineage. [file mmc2.pdf]

	Count of Lineage_Coll_name		
**-**	**3**		
ungrouped	3		
**Beijing**	**6**		
group_8	6		
**Cameroon**	**116**		
group_10	2		
group_11	3		
group_13	2		
group_15	3	grouped=	91
group_18	5		
group_19	4		
group_2	30		
group_23	3		
group_24	2		
group_27	6		
group_29	3		
group_3	2		
group_33	2		
group_34	4		
group_4	16		
group_50	2		
group_52	2		
ungrouped	25		
**Euro-American**	**50**		
group_31	12		
group_38	3	grouped=	17
group_51	2		
ungrouped	33		
**Haarlem**	**74**		
group_1	14		
group_16	27		
group_28	2		
group_30	2	grouped=	56
group_35	2		
group_41	3		
group_45	2		
group_49	4		
ungrouped	18		
**LAM**	**68**		
group_12	2		
group_14	2		
group_20	8		
group_36	2		
group_39	2	grouped=	40
group_40	3		
group_43	2		
group_5	17		

group_9	2		
ungrouped	28		
**mainly T**	**58**		
group_17	2		
group_21	3		
group_26	5		
group_32	2	grouped=	44
group_37	30		
group_44	2		
ungrouped	14		
**S-type**	**3**		
group_42	2	grouped=	2
ungrouped	1		
**Uganda**	**2**		
ungrouped	2		
**West-Africa 1**	**46**		
group_22	3		
group_25	2		
group_46	3		
group_47	3	grouped=	20
group_48	2		
group_6	2		
group_7	5		
ungrouped	26		
**West-Africa 2**	**3**		
ungrouped	3		
**X-type**	**1**		
ungrouped	1		
**Gesamtergebnis**	**430**		
